# Emissions of water-soluble polymers from household products to the environment: a prioritization study

**DOI:** 10.1093/etojnl/vgae030

**Published:** 2025-01-06

**Authors:** Hattie Brunning, J Brett Sallach, Alistair Boxall

**Affiliations:** Department of Environment and Geography, University of York, York, United Kingdom; Department of Environment and Geography, University of York, York, United Kingdom; Department of Environment and Geography, University of York, York, United Kingdom

**Keywords:** ecological risk assessment, environmental modeling, personal care products, contaminants of emerging concern, polyquaterniums

## Abstract

Water-soluble polymers (WSPs) are widely used in household products, including cleaning and personal care products. However, unlike insoluble plastic polymers, the environmental risks of WSPs are poorly understood. This study was performed to identify polymers in household use and characterize their emissions to the environment and key data gaps for prioritization. An inventory of polymers was developed and these were broadly grouped based on structure. Information from patents was combined with literature data to estimate down-the-drain emissions for each polymer. For the polymers with the highest emissions, predicted environmental concentrations for surface water and soil were estimated. A total of 339 individual polymers were identified and categorized into 26 groups. The polymers with the highest down-the-drain emissions were sodium laureth sulfate (1.6–3.4 g capita^−1 ^day^−1^), styrene/acrylates copolymer (0.1–0.8 g capita^−1 ^day^−1^), and monoethanolamine-laureth sulfate (0.4–0.8 g capita^−1 ^day^−1^). An analysis of available fate and ecotoxicity data for 30 key high-emission polymers indicated that several are lacking in data. In particular, no data were found for styrene/acrylates copolymer and copolymer of polyethylene glycol/vinyl acetate, and the environmental fate of polyquaterniums and polyol ethoxylate esters has been understudied, particularly in light of their hazard potential. However, a lack of reporting of key polymer properties hinders analysis. We recommend increased transparency in reporting of polymer identities moving forward as well as experimental work determining fate, removal, and hazard of the prioritized high-emission polymers that are lacking in data.

## Introduction

The environmental impact of polymers is an area of increasing interest to scientific and regulatory communities, given the widespread use, emissions, and potential persistence of these substances (e.g., European Centre for Ecotoxicology and Toxicology of Chemicals (ECETOC), 2019; Huppertsberg et al., 2020; Koelmans et al., 2017). In particular, the presence and impact of macro, meso, micro, and nano plastics in the environment has been the focus of substantial research (e.g., Burns & Boxall, 2018; Derraik, 2002; Koelmans et al., 2017; Thompson et al., 2009). However, the environmental risks of nonplastic polymers, such as water-soluble polymers (WSPs), have been relatively overlooked (Arp & Knutsen, 2020; Huppertsberg et al., 2020).

Until now, polymers have been exempt from many regulatory schemes for low molecular weight (MW) chemicals. Polymers were excluded under Registration, Evaluation, Authorisation and Restriction of Chemicals (REACH; European Parliament and Council, 2006) due to the wide range of polymers on the market and the assumption that they are likely low concern due to their high MW (European Chemicals Agency (ECHA), 2023). However, a fundamental aim of REACH is to operate under the precautionary principle, with decision-making based on potential to cause significant harm despite scientific uncertainty (Hansen et al., 2007). Recent progress towards incorporation of “polymers requiring registration” into REACH remains limited by several outdated assumptions regarding polymer properties (Groh et al., 2023), with more recent scientific data indicating the need for further study and environmental risk assessment (ERA) based on both potential hazards and environmental exposure of polymers.

The assumption that polymers with MW > 1,000 g mol^−1^ are generally biologically inert has been called into question (Groh et al., 2023). Uptake of polyethylene glycol (PEG) of up to 4,000 and 8,000 g mol^−1^ has been observed in fish embryos and tadpoles, respectively (Nascimento et al., 2021; Pelka et al., 2017), and toxic effects have been observed for PEG and various cationic WSPs despite low or absent cellular uptake (Connors et al., 2023; Nascimento et al., 2021). This is despite the fact that PEG has historically been classified as nontoxic (Duis et al., 2021). Toxicity of PEG and polyvinyl alcohol (PVOH) to fish and frogs has also been observed (Zicarelli et al., 2024), although several other studies have again found PVOH to be nontoxic (Alonso-López et al., 2021; McDonough et al., 2024; Nigro et al., 2022). Cationic polymers have been extensively studied due to their toxicity towards algae, fish, and invertebrates (e.g., Costa et al., 2014; Cumming et al., 2008; U.S. Environmental Protection Agency (USEPA), 1997; Connors et al., 2023; Hansen et al., 2023; Rawlings et al., 2022).

**Figure 1.**
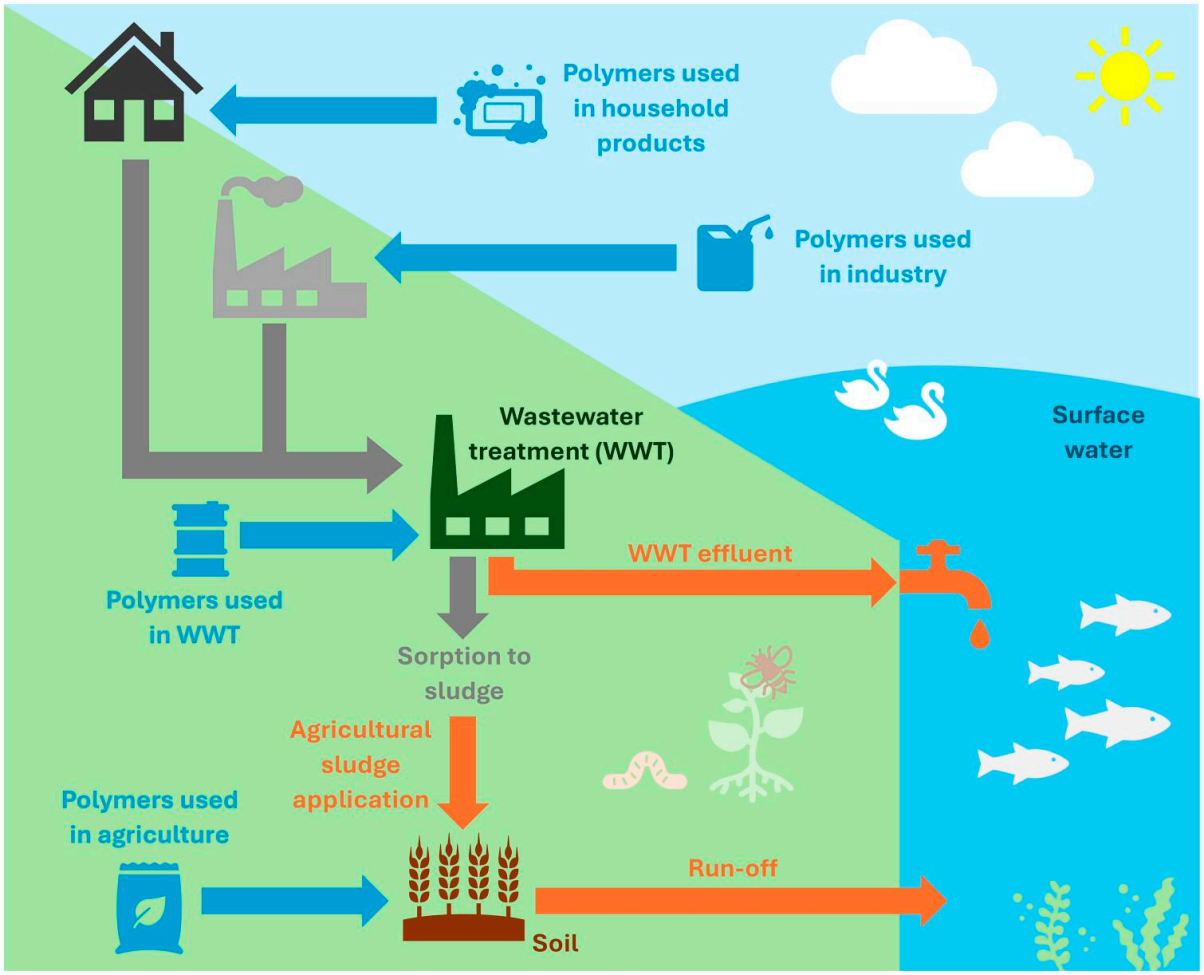
Summary of routes of exposure of the environment to water-soluble polymers (WSPs), with inputs from key applications of WSPs (blue) and direct routes of emission (orange) to surface water and soil.

There are several emission pathways of WSPs to the environment, given their widespread use in agriculture, wastewater treatment (WWT), and household products (Arp & Knutsen, 2020), with millions of tonnes of WSPs used in Europe annually (Huppertsberg et al., 2020). Water-soluble polymers in household products include ethoxylated compounds as surfactants in handwash (Cowan-Ellsberry et al., 2014), polycarboxylates as anti-redeposition agents in detergents (DeLeo et al., 2020; Soap and Detergent Association (SDA), 1996); and polyquaterniums as antistatic agents in hair products (Johnson et al., 2016), along with a range of other polymers with various functions. These polymers may be released down-the-drain to WWT and subsequently to surface waters or soil (Figure 1). Several WSPs have been measured in wastewater effluent, sewage sludge, and environmental waters up to the mg L^−1^ and µg g^−1^ range, including PEG, poly(vinylpyrrolidone), poly(N-vinylcaprolactam), poly(ethyleneimine), and cationic polyacrylamide (Antić et al., 2011; Jovancicevic & Schwarzbauer, 2023; Pauelsen et al., 2023; Vidović et al., 2022, 2023, 2024), showing considerable levels of WSPs have been released to the environment for over a decade, some of which are poorly biodegradable.

However, environmental concentration data for most WSPs are lacking (Duis et al., 2021; Huppertsberg et al., 2020). Characterization of emissions and environmental concentration is essential to determine exposure for ERA, and may also be useful in prioritizing WSPs for further data collection prior to full ERA (Groh et al., 2023). Pecquet et al. (2019) found that of 65 polymers identified in household cleaning products, 18 had insufficient data available to conduct an ERA. The authors recommended further prioritization based on usage volumes and concentration in products (among other criteria). The fate and effects of WSPs in cosmetic products (PEG, acrylic acid homo- and co-polymers, and polyquaterniums) were evaluated by Duis et al. (2021), and the authors highlighted a lack of exposure data limiting conclusive ERA, with insufficient analytical methods for polymer monitoring and a scarcity of usage volume data impeding determination of both measured and predicted environmental concentrations (MECs and PECs; Duis et al., 2021). Given the diversity and abundance of WSPs in current use, conducting even preliminary ERAs for all individual polymers is challenging, and it is essential to develop methods to estimate exposure when usage and emissions data are not directly available as input parameters for modeling.

In this study, PECs for surface water and soil of high emission polymers from household products were estimated based on a combination of publicly available product ingredients information, literature data, and emissions modeling, with application of various assumptions and chemical groupings to account for insufficient data. Biodegradability and ecotoxicity data were also assessed and used in combination with PECs to prioritize key polymers and their data gaps. Although the majority of these polymers are water-soluble and synthetic, insoluble and natural polymers were also included to allow all key polymers to be identified and prioritized. This was to highlight several key types of polymers that are likely to be released to the environment and provide preliminary concentration estimates and an assessment of research needs.

## Materials and methods

**Figure 2.**
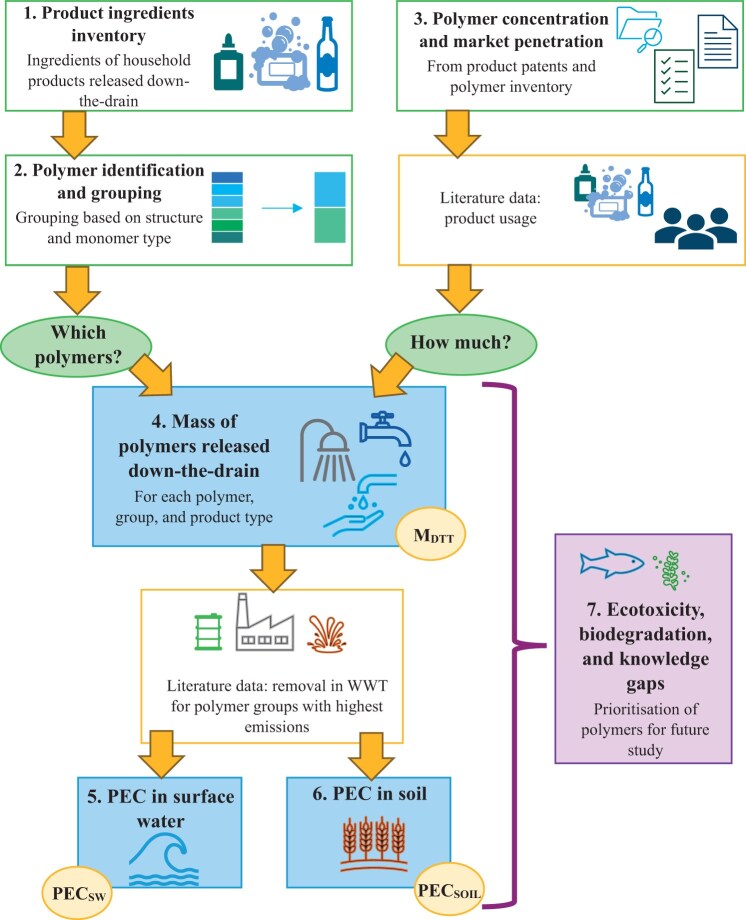
Summary of the emissions modelling and prioritization approach developed and used in this study. M_DTT_ = mass of polymer released down-the-drain; PEC = predicted environmental concentration; WWT = wastewater treatment; SW = surface water.

The overall methodology of the study is summarized in Figure 2, with each stage of the workflow outlined in detail in each of the following sections.

### Products, brands, and ingredients inventory

Household cleaning and personal care products released down-the-drain at point of use were identified from U.K.-based supermarket websites (See online supplementary material Data 1 ). The products included in the final dataset were laundry detergents, dishwashing detergents (for machine and washing by hand), toilet cleaners, and personal cleansers for skin (handwash, bodywash, soap bars, and bath liquids) and hair (shampoo and conditioner). Some product subtypes were analyzed together (e.g., liquid and powdered laundry detergent; toilet cleaner and bleach/disinfectant; and 3-in-1 personal cleansers and bodywash) under the assumption that usage patterns and polymer concentrations are likely similar.

Major brands for each product type were identified from websites of the top four U.K. supermarkets (Tesco, Sainsbury’s, Asda, and Morrisons; Coppola, 2021). For shampoo, conditioner, personal cleansers, and toilet cleaners, only brands listed by multiple websites were included in data collection, due to the large numbers of brands (> 45 in each case) initially identified. Supermarket own-brands were not included due to limited availability of ingredients data for some product types; formulations are also likely to be similar to other market brands.

Ingredients of all individual products from each brand were collated from publicly available information on brand and company websites, between April 2020 and May 2021. For some brands, information on ingredients was unavailable, so these brands were removed from the dataset. The number of brands included in the final study for each product type are shown in online supplementary material Data 2.

### Polymer identification and grouping

Polymers were identified following the Organisation for Economic Co-operation and Development (OECD) definition of a polymer (OECD, 1991), summarized as criteria 1-4 in Table 1, with two additional criteria (in keeping with the OECD polymer definition) used to narrow the scope of the study. Although enzymes were excluded (criterion 5), some other identified proteins/polypeptides were included (gelatin, keratin, wheat gluten, and whey protein) as these may consist of multiple proteins across a range of MWs (Bragulla & Homberger, 2009; Farrugia et al., 1998; Vensel et al., 2014; Wang & Lucey, 2003).

**Table 1.** Criteria for polymer identification in household products based on the Organisation for Economic Co-operation and Development definition of a polymer, and further exclusions applied in the present study in keeping with the established criteria.

Classification criteria based on definition of a polymer	
**Criterion 1**	The substance consists of molecules comprising a sequence of one or more types of monomer units.
**Criterion 2**	The substance comprises a simple weight majority of molecules containing ≥ 3 monomer units covalently bound to at least one other monomer unit or other reactant.
**Criterion 3**	The substance contains molecules distributed over a range of molecular weights with differences in molecular weight being primarily due to differences in the number of monomer units.
**Criterion 4**	The substance consists of less than a simple weight majority of molecules of the same molecular weight.
**Further exclusions applied in the present study**	
**Criterion 5**	Enzymes were excluded from the final dataset due to the fact that most enzymes will not fit the OECD definition of a polymer (USEPA, 1997).
**Criterion 6**	Silicates were excluded due to the fact that degree of polymerization is dependent on metal content, concentration, and pH, and upon release to the aquatic environment depolymerization is expected to occur (OECD, 2004).

Polymers were defined based on names listed in product ingredients, and where necessary, using information on chemicals provided by the European Chemicals Agency (ECHA, 2020), and databases such as PubChem, the Environmental Working Group (EWG) Skin Deep database, SpecialChem, The Good Scents Company (TGSC) Information System, ChemID*plus* (EWG, 2021; Kim et al., 2023; SpecialChem, 2021; TGSC, 2021), and Sigma Aldrich/Merck (Merck, 2021). In cases where insufficient information was available to make a definitive assignment (e.g., no information on average number of monomer units or MW majority [criteria 2 and 4]), most were assigned as polymers to give more conservative emissions estimates. Ingredients potentially identifiable as polymers but not included in the final dataset are listed in online supplementary material Data 3. In addition, although most identified polymers are water-soluble, solubility was not an applied criterion and thus some identified polymers were insoluble (e.g., styrene/acrylates copolymer, silicones, etc.; see *Results and discussion* section), to allow all key polymers in the studied products to be identified and prioritized.

Identified polymers were broadly categorized into groups based on structure, monomers, and functional groups. Structural features for classification of key polymer groups are shown in online supplementary material Data 8 (see also *Results and discussion* section). As further information on polymer properties relevant to grouping (including MW ranges, partitioning behavior, and charge density; ECETOC 2019) were not reported, these could not be accounted for and thus, further refinements and subgroups will likely be necessary for higher tier exposure assessment.

### Polymer concentration and market penetration

Concentrations of polymers (F_pol_) in products were obtained from patents identified using Google Patents. Search terms included product types (e.g., “laundry detergent composition”), and either individual polymers (e.g., “styrene/acrylates copolymer”) or polymer groups (e.g., “polycarboxylate”). The most preferred concentration ranges (percentage by weight) for each polymer or group were recorded for a minimum of three patents (where possible), or from the first 3–5 pages of search results. For example, if a patent listed polymer concentration as “generally 0.5 to 15%, preferably 0.5 to 10%, more preferably 1 to 5 wt. %”, values of 1% to 5% were recorded. Concentrations deemed most representative of the acquired patents for each polymer group, while generally accounting for higher concentrations to provide more conservative estimates, were selected for use in emissions modeling (See online supplementary material Data 4). It was assumed that individual polymers within groups would perform similar technical functions in products, and thus products containing multiple members of a polymer group were assumed to have the same concentration ranges as products containing only one member of a group. For example, if a detergent product listed two alcohol ethoxylate polymers in its ingredients, it was assumed these would both contribute to total nonionic surfactant concentration, rather than each being used separately at the patented concentration of nonionic surfactants, to avoid unrealistically high estimations of polymer concentration. In some cases, polymer concentrations were difficult to estimate; for example, PVOH is most commonly used as a film surrounding detergent capsules or tablets, however, mass concentrations of such films were not found. Concentrations reflecting use of PVOH in a dissolved or dispersed form in the products were instead used.

Estimates of market penetration (F_prod_) were calculated for each polymer group and product type, as the fraction of products containing one or multiple polymers belonging to each group (*Equation 1*, See online supplementary material Data 5).


(1) $$F_{\mathrm{prod}}=\frac{N_{\mathrm{prod}}}{T_{\mathrm{prod}}}$$

where F_prod_ is the estimate of market penetration, N_prod_ is the number of products of a particular type containing one or multiple members of the polymer group, and T_prod_ is the total number of products of the selected type included in the dataset. This approach thus made use of widely available product ingredients data to estimate proportions of products containing each polymer type, allowing generation of usage estimates where market penetration and production and import volumes are not publicly available.

### Down-the-drain mass emissions

Usage data (U_prod_) in g capita^−1 ^day^−1^ for each product type were obtained from the literature (See online supplementary material Data 6). Masses of polymer groups emitted down-the-drain from each product type were estimated using *Equation 2*.


(2) $$M_{\mathrm{DTT}(\mathrm{prod})}=F_{\mathrm{prod}}\times F_{\mathrm{pol}}\times U_{\mathrm{prod}}$$

where M_DTT(prod)_ is the mass of polymer released down-the-drain from a particular product type (g capita^−1 ^day^−1^), F_prod_ is the fraction of products containing polymer type, F_pol_ is the fractional concentration of polymer in product (from % by weight), and U_prod_ is the product usage (g capita^−1 ^day^−1^). Ranges of F_pol_ values given by patents were used, giving a range of values for M_DTT(prod)_ for each polymer group.

Total M_DTT_ estimates for each polymer group were then obtained as the sum of estimates for each product type according to *Equation 3*.


(3) $$M_{\mathrm{DTT}}=\sum_{\mathrm{all}\;\mathrm{product}\;\mathrm{types}}M_{\mathrm{DTT}(\mathrm{prod})}$$

To estimate M_DTT_ for individual polymers within groups, contribution to total group M_DTT(prod)_ from each polymer was calculated from its number of occurrences (as a fraction of total occurrences of all polymers in the group, for each product type), and thus applied to M_DTT(prod)_ and summed as above. This approach was taken to account for the fact that different product types contain these individual polymers to varying extents, but F_pol_ estimates were assumed to represent total contribution from each group (as described for derivation of F_pol_ above).

### Surface water exposure

Estimates of surface water exposure (PEC_SW_) for the top 10 polymer groups with the highest M_DTT_ were obtained across the studied products (*Equation 4*), based on the method given by the European Medicines Agency (EMA; 2018; adapted from ECHA 2016).


(4) $${\mathrm{PEC}}_{SW}=\frac{M_{\mathrm{DTT}}(1-F_{\mathrm{WWT}})}{{WW}_{\mathrm{INHAB}}\times DF}$$

where PEC_SW_ represents the predicted environmental concentration in surface water (µg L^−1^), M_DTT_ is the mass of polymer group released down-the-drain (µg capita^−1 ^day^−1^), F_WWT_ is the fraction removed from water in wastewater treatment, WW_INHAB_ represents the amount of wastewater per inhabitant per day (L capita^−1 ^day^−1^), and DF shows the dilution factor for entering surface water. Default values for WW_INHAB_ and DF of 200 L capita^−1 ^day^−1^ and 10, respectively, were used (EMA 2018; ECHA 2016). Wastewater treatment removal data (F_WWT_) were obtained from the literature; Web of Science and Google Scholar were searched for specific polymers or groups (with the relevant polymer names being listed in online supplementary material Data 8) and “wastewater” or “wastewater treatment”. Where multiple values were available, both within and between different sources, the highest and lowest values were applied to the lowest and highest bounds of the M_DTT_ estimates to account for the most and least conservative scenarios. This also allowed for the fact that many groups contained a broad range of polymers, which may exhibit different properties and fate in wastewater treatment.

The PEC_SW_ estimates for individual polymers within groups were calculated from the contributions of each polymer as described above for derivation of M_DTT_ for individual polymers. This approach was taken due to the lack of specific structural information (e.g., molecular weight) for most polymers, as it allowed calculations to be made for polymer groups as a whole (reducing the requirement for structurally specific input data for F_WWT_) whilst estimating relative exposure of individual polymers from their relative use in products.

### Soil exposure

Concentrations of polymers in sludge following wastewater treatment were calculated using *Equation 5* for the top 10 polymer groups with the highest M_DTT_.


(5) $$C_{\mathrm{SLUDGE}}=\frac{M_{\mathrm{DTT}}\times F_{\mathrm{SLUDGE}}}{S_{\mathrm{INHAB}}}$$

where C_SLUDGE_ represents the concentration of polymer group present in sludge (mg kg^−1^, dry wt), F_SLUDGE_ is the fraction of polymer partitioned to sludge in WWT, and S_INHAB_ is the mass of sludge per inhabitant per day (kg capita^−1 ^day^−1^, dry wt). A value for S_INHAB_ of 0.074 kg capita^−1 ^day^−1^ was used (Guo et al., 2016). Values of F_SLUDGE_ were obtained from the literature and/or F_WWT_ (see *Results and Discussion* section).

Estimates of soil exposure (PEC_SOIL_), assuming no degradation of polymers following emission, for sludge-amended soil after the first year of sludge application were determined for each polymer group according to *Equation 6*, based on guidance given by ECHA (ECHA 2016).


(6) $${\mathrm{PEC}}_{\mathrm{SOIL}}=\frac{C_{\mathrm{SLUDGE}}\times A_{\mathrm{SLUDGE}}\times 1\;\mathrm{year}}{D_{\mathrm{SOIL}}\times {\mathrm{RHO}}_{\mathrm{SOIL}}}$$

where PEC_SOIL_ is the predicted environmental concentration in sludge-amended soil (mg kg^−1^), A_SLUDGE_ represents the dry sludge application rate to land (kg m^−2 ^yr^−1^), D_SOIL_ is the soil mixing depth (m), and RHO_SOIL_ is the bulk density of soil (kg m^−3^; ECHA 2016; Guo et al., 2016). Default values for A_SLUDGE_, D_SOIL_, and RHO_SOIL_ of 0.5 kg m^−2 ^yr^−1^, 0.2 m, and 1,700 kg m^−3^, respectively, were used (ECHA 2016; Guo et al., 2016).

Estimates of PEC_SOIL_ for individual polymers within groups were calculated using the same method as for estimates of individual M_DTT_ and PEC_SW_ as described above.

### Biodegradation and ecotoxicity data

Biodegradation and environmental effects (hazard) data were gathered from the literature for the ten highest-emission groups to further assess data gaps and research needs. For three groups, data had been previously compiled (Human & Environmental Risk Assessment (HERA) project 2004, 2009, 2014a, 2014b), and thus these data were used in the present study. For the remaining seven groups, searches were conducted using the ECOTOX Knowledgebase (USEPA, 2000) and Google Scholar; search terms included generic group names (e.g., “polyquaternium”) and specific polymer names (e.g., “aziridine homopolymer”), and in Google Scholar, were combined with additional terms (“biodegradation”, “ecotoxicity”, “fish toxicity”, “algae toxicity”, or “*daphnia* toxicity”). Existing PECs and MECs were also collated from the literature to compare estimates of the present study and evaluate the accuracy of PECs.

## Results and discussion

### Identified polymers, market penetration, and polymer grouping

A total of 339 individual polymers were identified (See online supplementary material Data 8) across 1,353 products and 10 product types (laundry detergent, machine and hand dishwashing detergent, toilet cleaner, bodywash, handwash, soap bars, bath liquid, shampoo, and conditioner). For most polymers, certain key information was not reported in product ingredients, including chemical names, Chemical Abstracts Service (CAS) numbers, MW, and charge density. Chemical Abstracts Service numbers could not be identified in a meaningful way for most polymers, because many are associated with multiple CAS numbers, and specific polymer compositions used were not reported. Information on monomer ratios was unavailable, and often not all monomer types were specified for copolymers. All identified polymers are therefore reported in this study as listed in the product ingredients, with the stipulation that for many polymers, multiple naming conventions exist that may be ambiguous; for example, PEG-150 may refer to PEG of average MW 150 g mol^−1^ or PEG with an average of 150 monomer units, and “sodium acrylates copolymer” and “acrylic copolymer” could contain the same or different monomers (e.g., polymers with CAS numbers 25035-69-2 and 25133-97-5 both have “acrylates copolymer” listed as an identifier by ECHA, despite having different monomers [ECHA 2020]). Despite these data limitations, key types of polymers likely to be released down-the-drain could be identified. As emissions data are severely lacking for most WSPs, and manufacture/import volumes are typically not publicly available (Duis et al., 2021), these data are a useful first step to addressing this data gap for exposure and risk assessment until further data become available.

**Figure 3.**
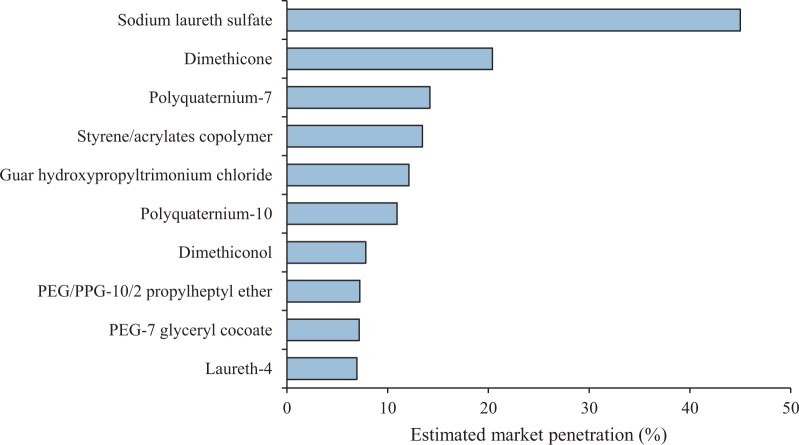
Estimated market penetration of the top 10 individual polymers (by market penetration) across all of the studied U.K. down-the-drain household products, shown as percentage of products containing polymers.

The polymer identified in the most products was sodium laureth sulfate, an anionic ethoxylated fatty alcohol commonly used as a surfactant in home and personal care products (Robinson et al., 2010), present in almost half the products studied (Figure 3). Note that although number of monomer units (*n*) is often < 3 for alcohol ethoxysulphate compounds in household products (which technically does not fulfil the OECD polymer definition), longer chain lengths are also used (e.g., *n* = 8; HERA 2004, See online supplementary material Data 8). Therefore, sodium laureth sulfate (and other compounds with unspecified n) may include both polymeric and nonpolymeric material (based strictly on the OECD polymer definition). However, in reality there is no chemical cut-off between polymers with an average of 3 and 4 monomer units, and low MW “nonpolymers” will have similar properties to low MW “OECD polymers” and may contribute to similar environmental effects as a mixture. Other commonly occurring polymers (present in > 10% of products studied) included dimethicone, polyquaternium-7, styrene/acrylates copolymer, guar hydroxypropyltrimonium chloride, and polyquaternium-10 (Figure 3).

Although the vast majority of polymers assessed in this study are water-soluble, some polymers such as dimethicone and styrene/acrylates copolymer were not WSPs. However as nonplastic polymers, many have received little attention in the context of environmental risk. It was therefore considered appropriate to include all identified polymers in the dataset to provide an overview of key polymer types, many of which have been rarely studied (see *Knowledge gaps and future applications* section).

**Figure 4.**
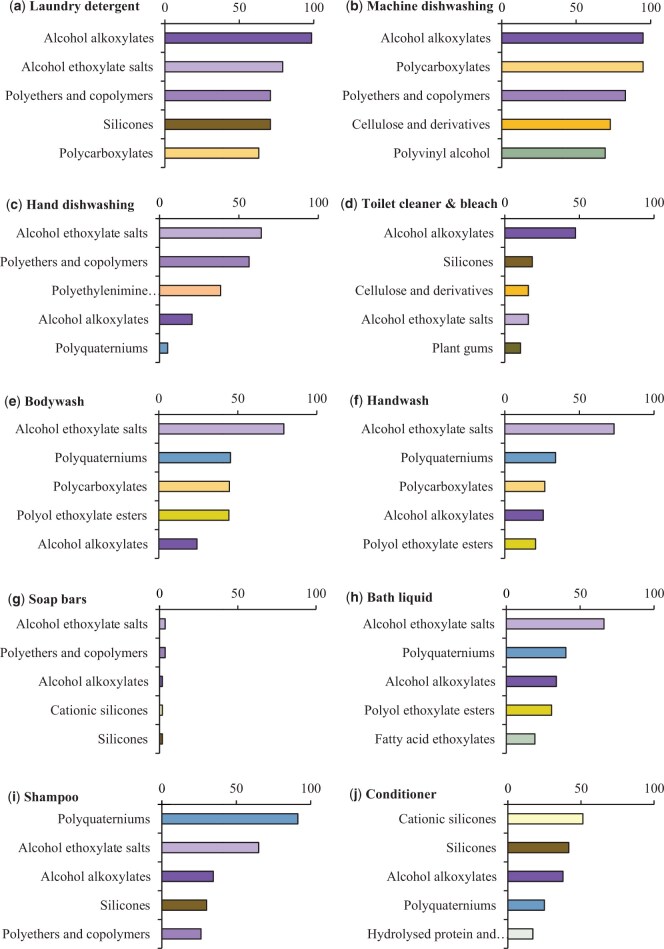
Estimated market penetration of the top five polymer groups (by market penetration) in each of the studied product types (U.K. down-the-drain household products), shown as percentage of products containing polymer groups. Polymer groups are colored for ease of comparison between graphs.

The 339 identified polymers were categorized into 26 groups (See online supplementary material Data 8), based on monomer type, polymer structure and functional groups, and expected functions in products, with the exception of one group (“other”; containing 15 remaining unrelated polymers). These 15 polymers were analyzed separately to obtain individual emissions estimates before being combined into a group. The most common polymer groups by market penetration included alcohol ethoxylate salts and alcohol alkoxylates (used as anionic and nonionic surfactants, respectively; e.g., Cowan-Ellsberry et al., 2014), and polyquaterniums (used as antistatic and film-forming agents; e.g., Johnson et al., 2016). Other key groups included polycarboxylates, silicones, polyethers and copolymers, and polyol ethoxylate esters (Figure 4, See online supplementary material Data 5). The most prevalent groups by market penetration differ by product type; for example, cationic silicones are in the top five polymer groups by market penetration for conditioner and soap bars, with cellulose polymers being prevalent in machine dishwashing detergents and toilet cleaners. Some product categories contained certain polymer groups in close to 100% of the products studied (e.g., laundry detergent, machine dishwashing detergent, shampoo), whereas other product types had no polymer groups present in more than about half of the products (conditioner, toilet cleaner). Soap bars had the lowest market penetration of all polymers, with all groups present in < 4% of the products studied.

### Down-the-drain emissions

**Figure 5.**
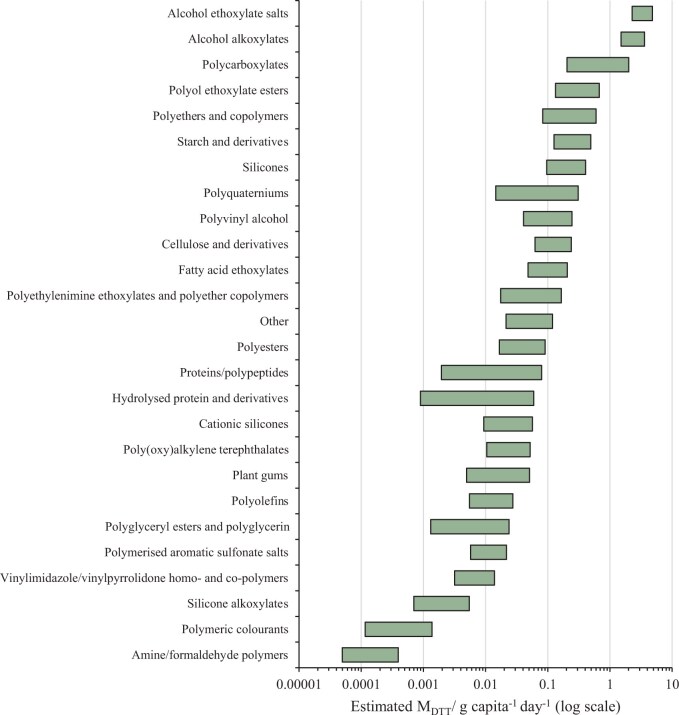
Estimates of down-the-drain mass emissions (M_DTT_) for identified polymer groups in U.K. household products. Ranges reflect minimum and maximum estimates (derived from minimum and maximum patented concentrations of polymers in products).

Total M_DTT_ estimates for polymer groups (i.e., cumulative emissions for each entire group) were in the range of 4.9E-05 g capita^−1 ^day^−1^ (amine/formaldehyde polymers) to 4.8 g capita^−1 ^day^−1^ (alcohol ethoxylate salts; Figure 5; see online supplementary material Data 7).

**Figure 6.**
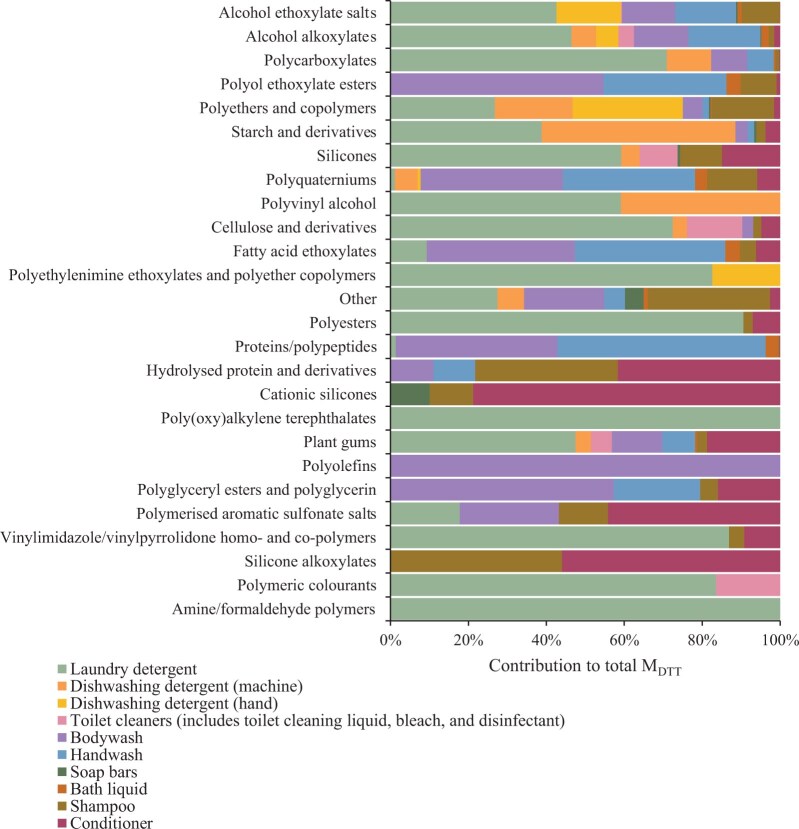
Contribution to total down-the-drain emissions (M_DTT_) for each polymer group from each of the product types (U.K. down-the-drain household products) included in the study.

Laundry detergents were the major contributor to total M_DTT_ estimates for many polymer groups, including several of the 10 groups with the highest M_DTT_. For example, laundry detergents contributed 72% to the M_DTT_ of cellulose and derivatives, 71% to polycarboxylates, and 59% to each of silicones and polyvinyl alcohol (Figure 6). Handwash and bodywash also were major contributors, collectively, for several groups, including polyol ethoxylate esters (86%) and polyquaterniums (70%). The high contributions from these three product types reflect high usage rates of 11.3, 10.3, and 8.3 g capita^−1 ^day^−1^ for laundry detergent, handwash, and bodywash, respectively (Eriksson et al., 2002; Garcia-Hidalgo et al., 2017; International Association for Soaps, Detergents and Maintenance Products (A.I.S.E.), 2019), which were notably higher than values for other product types (See online supplementary material Data 6). However, for a small number of groups, other product types contributed more significantly to M_DTT_ (Figure 6), reflecting higher concentrations and greater market penetration.

Estimates of M_DTT_ for individual polymers within groups ranged from 2.7E-06 g capita^−1 ^day^−1^ (wheat gluten, proteins/polypeptides group) to 3.4 g capita^−1 ^day^−1^ (sodium laureth sulfate, alcohol ethoxylate salts group). For some groups, M_DTT_ was largely made up of only a few specific polymers; for example, 70% of the total M_DTT_ of alcohol ethoxylate salts was from sodium laureth sulfate alone (1.6–3.4 g capita^−1 ^day^−1^; see online supplementary material Data 8). Other groups showed a wider distribution, with e.g., alcohol alkoxylates having the highest emitted polymer (laureth-4) contributing only 14% (0.2–0.5 g capita^−1 ^day^−1^) to the total group M_DTT_ (See online supplementary material Data 8). The polymers with the highest M_DTT_ overall were sodium laureth sulfate, styrene/acrylates copolymer, and monoethanolamine (MEA)-laureth sulfate, with estimated M_DTT_ of 1.6–3.4, 0.1–0.8, and 0.4–0.8 g capita^−1 ^day^−1^, respectively (See online supplementary material Data 8). However, for highly homogenous groups, for example, alcohol ethoxylate salts, study of exposure as a total mixture may be worthwhile, because these are likely to have similar fate behavior and ecotoxicological effects. Increased transparency in reporting of polymer structure and MW for other groups (e.g., polycarboxylates) will help clarify which subgroups may be necessary and which group members should potentially be assessed as a mixture.

### Wastewater treatment and PEC calculation

The top ten polymer groups with the highest M_DTT_ (Figure 5) were prioritized for calculation of PECs, incorporating removal in WWT (F_WWT_ and F_SLUDGE_) based on available literature data (Table 2). Grouped polymers were analyzed together for WWT removal to facilitate data collection and reduce data requirements, and due to the lack of available data for most polymers.

**Table 2.** Estimates from the literature of removal from wastewater (F_WWT_) and fraction present in sludge (F_SLUDGE_) used to calculate estimates of surface water exposure (PEC_SW_) for members of the top 10 prioritized polymer groups (based on down-the-drain emissions) identified in this study.

Polymer group	Fraction removed from water (F_WWT_)	Fraction present in sludge (F_SLUDGE_)	Summary of data	Data quality and representativity of the selected data for the respective polymer group	References
**Alcohol ethoxylate salts**	0.697–0.999	0.021–0.03	F_WWT_ values for alkyl/alcohol ethoxy sulfates (C = 12–15, EO = 0–8). Obtained from monitoring of influent and effluent of WWT. Lowest and highest removal estimates used. 69.7 % = trickling filter, 99.9 % = activated sludge. Key group members expected to be readily biodegradable with approx. 97% degraded for C = 12–15, EO = 2.7. Therefore assuming majority of removed fraction (97%) is degraded gives 2.1–3.0% released in sludge.	Monitoring data cover range of removal across a total of 11 activated sludge WWT plants (across the US and Netherlands) and six trickling filter plants (US), and thus are likely to cover the range of removal which may occur. Monitoring data cover most of likely range of C and EO chain lengths across entire group in present study (predominantly C = 12–15, minor contribution from C = 8–18, EO = predominantly 8 or unspecified with minor contribution from EO = 30) and thus are expected to be representative of the group which is relatively homogeneous. Degradation data are derived from SimpleTreat scaled using experimental data for which no consistent trend was observed with C or EO chain lengths, and thus these are expected to be representative of the group.	HERA, 2004; Matthijs et al., 1999; McAvoy et al., 1998
**Alcohol alkoxylates**	0.794–0.999	0.008–0.01	Values for alcohol ethoxylates (C = 12–18, EO = 0–18 or average EO assumed to be 9). Obtained from monitoring of influent and effluent of WWT. Lowest and highest removal estimates used. 79.4 % = trickling filter, 99.9 % = oxidative ditch, trickling filter, activated sludge. Shorter ethoxylate chains expected to be readily biodegradable (more than 99% degradation for C = 13–16, EO = 9). Therefore assuming majority of removed fraction (99%) is degraded gives 0.8–1.0% released in sludge.	Monitoring data cover range of removal across a total of 13 activated sludge WWT plants (across the US and Netherlands), and 8 trickling filter, 2 oxidative ditch, 2 lagoon, and 1 rotating biological contactor plant(s) (US), and thus are likely to cover range of removal which may occur. Monitoring data cover the most common ranges of C and EO chain lengths observed in the group of the present study; however, note that PPG ethers are also present which may require further study, as well as longer EO chain lengths (e.g., EO = 80). Degradation data were obtained by prediction of degradation in WWT from batch tests with activated sludge. Levels were consistent with other studies and thus assumed to be accurate. Degradation data are for typical C and EO chain lengths, however again further research into polypropylene glycol (PPG) ethers and longer-chain polyethylene glycol (PEG) which make up minor contributions to the group may be necessary to refine PEC estimates.	Federle & Itrich, 2006; HERA, 2009; Matthijs et al., 1999; McAvoy et al., 1998; Morrall et al., 2006
**Polycarboxylates**	0.09–0.98	*—*	9 % = homopolymer of acrylic acid (PAA), mean MW 1,000 g mol^–1^, OECD 303 A (Activated sludge simulation test), DOC influent concentration 15 mg/L. 98 % = copolymer of acrylic/maleic acid (PAA-MA), mean MW 70,000 g mol^–1^, OECD 303 A (Simulation test), DOC removal.	Data show the likely range of removal obtained from OECD 303 tests which are well established for assessing removal in WWT. Data were assigned Klimisch scores of 1 and 2 for 9 and 98% removal, respectively, in the Human & Environmental Risk Assessment (HERA) reports, which are both sufficient for accepting the data. Data cover a range of molecular weights for PAA and PAA-MA (1,000–4,500 and 12,000–70,000 g mol^–1^, respectively), however molecular weight data were not obtainable in the present study and thus further data may be required to determine more specific removal rates. Similarly other polycarboxylate polymers are included in the present study for which there are currently no data on WWT removal, particularly styrene/acrylates copolymer which is a significant contributor to the group; however, the removal range utilized (9–98%) is very wide and thus is likely to cover actual removal of these polymers for which there are currently no data. Further data will be useful in refining removal estimates.	HERA, 2014a, 2014b
**Polyol ethoxylate esters**	*—*	*—*	Literature values for WWT removal not found. Therefore F_WWT_ = 0 and F_SLUDGE_ = 1 was assumed for calculation of PEC_SW_ and PEC_SOIL_, respectively, to give a conservative worst-case estimate for each.	Further data are required to determine actual removal and refine PEC estimates.	—
**Polyethers and copolymers**	0.70–0.96	0.41	70 % = PEG-8000 (Pluriol E 8000; 8,000 g mol^–1^), OECD 303A (simulation test—aerobic sewage treatment)/ISO 11733 (activated sludge simulation test), DOC reduction (56 d). 96 % = PEG-400 (^14^C-labelled; 400 g mol^–1^), OECD confirmatory test: continuous activated sludge model WWT plant, 3 days , ^14^C mass-balance at test end; 4% of polymer in effluent, 41% in sludge.	Value for PEG-400 from OECD simulation test with an assigned Klimisch score of 2 by Duis et al. (2021) which is sufficient for accepting the data. The Klimisch score for the PEG-8000 value was not assignable (Duis et al., 2021), however this was obtained from an OECD 303 test which is well established for assessing removal in WWT. This value was thus included to give a more conservative lower estimate given the lack of data on PEG of higher molecular weights (see following discussion). Data cover removal from simulation tests for PEG of molecular weight 400–8,000 g mol^-1^; further data are thus required to refine PEC estimates for PPG, PEG copolymers, and PEG of higher molecular weights (up to 180,000 g mol^-1^), all of which were identified in the present study.	BASF, 2018; Duis et al., 2021; Steber & Wierich, 1985
**Starch and derivatives**	0.50	*—*	Literature values for WWT removal not found. However, starch is expected to be readily biodegradable and is faster to degrade than cellulose. Therefore a value of at least 50% (see cellulose and derivatives) can be assumed.	Further data are required to determine actual removal and refine PEC estimates.	Van Ginkel & Gayton, 1996
**Silicones**	0.94–0.97	*—*	94 % = polydimethylsiloxane (PDMS), average molecular weight < 14,000 g mol^-1^ (estimated from gel permeation chromatogram), monitoring of WWT in North America. 97 % = PDMS, based on WWT models and laboratory scale calculations.	Lower estimate (94%) based on monitoring of five activated sludge, two trickling filter, and one rotating biological contactor WWT plant(s), with measured removal >94% at all sites. Given the wide range of WWT plants studied this value is likely to be an accurate lower estimate for removal. Upper limit (97%) is an estimate only, reported by Graiver et al. (2003) based on previous studies; however, given that PDMS is frequently not detected in WWT effluent and 94% is the likely lower limit, 97% was deemed a sensible upper limit. Both estimates are for PDMS (dimethicone), the highest contributor to the silicones group in the present study, with many other group members such as dimethiconol and simethicone being highly similar and the group being relatively homogeneous. However, it may be relevant to adjust PEC estimates where data become available for other silicones in the group if removal is found to significantly differ to these estimates.	Fendinger et al., 1997; Graiver et al., 2003
**Polyquaterniums**	0.081–0.38	*—*	8.1 % = Polyquaternium-28 (Gafquat HS100, Chemical Abstracts Service (CAS) 53633-54-8), 38 % = Polyquaternium-6 (poly(DADMAC), CAS 26062-79-3). Equifugacity model used to predict removal of various polyquaternium compounds in WWT. Lowest and highest estimates are used here, which encompass estimates for various polyquaterniums (polyquaterniums –6, –10, –11, –28, and –55, and cetyl pyridinium chloride) of various trade names (see reference for further information).	The data obtained are from modelling only and thus further data (from experimental simulation studies and/or monitoring, where analytical methods become available) are required to accurately determine removal in WWT. However, the data provide a useful preliminary estimate for a range of polyquaterniums which were identified in the present study to determine preliminary PEC estimates and supplement polymer prioritization, given that further data are not available.	Cumming et al., 2011
**Cellulose and derivatives**	0.50	*—*	Carboxymethyl cellulose (CMC), degree of substitution 0.7. CAS test developed from OECD Test Guideline 303 A, 14 days.	Data were obtained from an adapted OECD 303 test which is well established for determining removal in wastewater treatment, for a key polymer in the group identified in the present study. Further data may be useful to confirm that other members of the group behave similarly, although the group is relatively homogeneous.	Van Ginkel & Gayton, 1996
**Polyvinyl alcohol (PVA)**	0.8424	0.6120	Model based on literature data for PVA degradation in critical processes of WWT plants. Mass balance; estimated that ∼61.20% of PVA is emitted via sludge, and ∼15.76% is emitted via effluent.	The data obtained are modelled based on an extensive review of the literature, with data from degradation studies in various WWT types and accounting for sorption and partitioning to sludge. Therefore these values are likely the best estimates for overall removal of PVA in typical WWT. All group members are PVA and therefore estimates are representative of the entire group.	Rolsky & Kelkar, 2021

*Note*: OECD = Organisation for Economic Co-operation and Development WWT = wastewater treatment.

Where F_SLUDGE_ is not specified, the value for F_WWT_ was used. Where reported in the original references, additional polymer information such as molecular weight, chain length, and CAS number is also specified.

Some data on F_WWT_ were available for most groups (Table 2). For polyethers and copolymers, polycarboxylates, and cellulose and derivatives, data were available from OECD simulation experiments. For alcohol ethoxylate salts and alcohol alkoxylates, data from degradation experiments were combined with data from monitoring studies to estimate removal. Monitoring data were also available for silicones to estimate F_WWT_, and data for PVOH were based on an extensive literature review with data from various degradation studies (Rolsky & Kelkar, 2021). However, for polyquaterniums, only modeling data were available, and for polyol ethoxylate esters and starch, data were not found. For polyol ethoxylate esters, values of F_WWT_ = 0 and F_SLUDGE_ = 1 were used (i.e., assuming all of this polymer type is either released in effluent or partitioned to sludge, respectively) meaning both PECs for this polymer group represent conservative worst-case scenarios for surface water and soil. For starch, significant biodegradation is likely; an estimate of 50% degradation was thus used from the data for cellulose, which will degrade more slowly than starch, to provide a conservative estimate while accounting for likely biodegradation. For these polymers, information on relative removal via partitioning and degradation was unavailable, and thus F_WWT_ = F_SLUDGE_ was applied for starch and cellulose, assuming the nondegraded fraction is entirely partitioned to sludge for PEC_SOIL_ calculations.

Moreover, F_SLUDGE_ = F_WWT_ was also applied to groups expected to have limited biodegradability (polycarboxylates, silicones, and polyquaterniums; i.e., no degradation assumed). For the remaining four groups (alcohol ethoxylate salts, alcohol alkoxylates, polyethers and copolymers, and polyvinyl alcohol), derivation of the fraction present in sludge (F_SLUDGE_) was possible (i.e., accounting for degradation).

For five groups (alcohol ethoxylate salts, alcohol alkoxylates, polyethers and copolymers, silicones, and polyvinyl alcohol), removal estimates were relatively high, ranging from approximately 70% to close to 100% (Table 2), suggesting relatively small proportions of these polymers are released in treated effluent. For polycarboxylates, although the upper estimate of WWT removal was also high (98%), the lower estimate (9%) indicates high variation depending on polymer structure and MW (HERA 2014a, 2014b). However, due to the lack of MW data for polycarboxylate polymers identified in this study (with only generic names such as “polyacrylic acid” being reported), and lack of specific structural data for some polymers, it was not possible to further subdivide this group to refine removal estimates and subsequent PEC. In addition, styrene/acrylates polymer is insoluble and thus likely to be efficiently removed from water in WWT by flocculation; however, in the absence of specific WWT removal data for this polymer, the full range of 9%–98% removal applied to polycarboxylates was used (assuming 98% removal is realistic for an insoluble material, but 9% removal provides a conservative estimate where specific data are lacking), with the stipulation that PEC_SW_ are likely towards the lower end of the estimated range for this polymer, and vice versa for PEC_SOIL_ (see subsequent sections). For the remaining two polymer groups (polyquaterniums, and cellulose and derivatives), WWT removal was estimated at ≤ 50% (Table 2), suggesting relatively low removal rates for these polymers and high potential for release in WWT effluent.

### Predicted environmental concentration in surface water

Total PEC_SW_ estimates for whole polymer groups (See online supplementary material Data 9a) ranged from 0.8 µg L^−1^ (alcohol alkoxylates) to 915 µg L^−1^ (polycarboxylates). Estimates of PEC_SW_ for individual polymers ranged from 7E-05 µg L^−1^ (coceth-7, alcohol alkoxylates group) to 512 µg L^−1^ (sodium laureth sulfate, alcohol ethoxylate salts group; see online supplementary material Data 9c). The three polymers with the highest PEC_SW_ were sodium laureth sulfate (0.8–512 µg L^−1^), styrene/acrylates copolymer (0.8–349 µg L^−1^), and sodium polyacrylate (0.4–160 µg L^−1^). Although the 10 polymers with the highest M_DTT_ all belonged to the alcohol ethoxylate salts, polycarboxylates, alcohol alkoxylates, and starch groups, the top 10 polymers in terms of PEC_SW_ also included polyquaternium and polyol ethoxylate ester polymers, with PEG-7 glyceryl cocoate, polyquaternium-7, and PEG-200 hydrogenated glyceryl palmate having PEC_SW_ of 20–99, 2.7–84, and 15–74 µg L^−1^, respectively.

For polycarboxylates, PEC_SW_ from this study were in good agreement with literature values (See online supplementary material Data 10a). Total PEC_SW_ from polyacrylic acid (PAA) within this group estimated in our study ranged from 0.5 to 222 µg L^−1^, and literature studies report PEC_SW_ of 70–570 µg L^−1^ (DeLeo et al., 2020) and 43–110 µg L^−1^ (HERA 2014a) for this polymer. Similarly, total PEC_SW_ from polyacrylic acid-maleic acid copolymers (PAA-MA) in the present study ranged from 0.4 to 166 µg L^−1^, and literature studies report PEC_SW_ of 20–130 µg L^−1^ (DeLeo et al., 2020) and 35–49 µg L^−1^ (HERA 2014b). However, these literature data cover only PAA and PAA-MA, leaving other group members unstudied.

Literature data for silicones and polyquaterniums were also generally in good agreement with data of the present study (See online supplementary material Data 10a). Fendinger et al. (1997) reported MECs of dimethicone (polydimethylsiloxane) in receiving water of < 5–7 µg L−1 (however, note that all but one sample were below the limit of detection at 5 µg L^−1^). Total PECSW for dimethicone and simethicone (which is a mixture of dimethicone and SiO2) was estimated at 0.8–7 µg L^−1^ in our study. For polyquaterniums, the literature value of PECSW = 0.72 µg L^−1^ for polyquaternium-68 (Australian National Industrial Chemicals Notification and Assessment Scheme [NICNAS] 2009) also shows close agreement with our study, with the PECSW of polyquaternium-68 being calculated as 0.02–0.6 µg L^−1^. However, Cumming (2008) reported PECSW for polyquaterniums in Australia at 0.039–0.46 µg L^−1^, which is significantly lower than the range of 5–142 µg L^−1^ for the entire polyquaterniums group in our study. The author noted insufficient data for estimation of the mixture of polyquaterniums present, including range of charge densities and MW, and thus a general approach was applied to estimate PECSW using a “theoretical” polyquaternium with properties typical of other identified polymers (Cumming, 2008), similar to the broad WWT removal approach used in our study. However, the author also noted incomplete manufacture and import estimates (Cumming, 2008), whereas the methods of this our study did not rely on production or import volumes.

Comparison of estimates from our study with literature data for other polymer groups is less straightforward. For alcohol ethoxylate salts, surface water MECs range from 0.01 to 10.3 µg L^−1^ (Popenoe et al., 1994; Sanderson et al., 2006; See online supplementary material Data 10a), and literature PEC_SW_ range from 0.42 to 54.87 µg L^−1^ (Environment and Climate Change Canada (ECCC) 2019, See online supplementary material Data 10a). Although most literature data report values for specific ranges of carbon (C) and ethoxy (EO) chain lengths, most members of this group did not have reported EO chain lengths or MW in our study and thus only comparison to the entire group is possible. The PEC_SW_ of our study ranges from 1 to 731 µg L^−1^ for this group, and although there is significant overlap with literature data, the higher upper estimate suggests greater transparency in reporting of polymer chain lengths and MW distributions may allow more direct comparisons and thus better evaluation of PEC_SW_ estimates.

Similarly, for alcohol alkoxylates, although most group members have reported C and EO chain lengths, each individual polymer still contains a distribution of chains across a range of MWs, and actual mixtures and distributions cannot be estimated from available data. The summed PEC_SW_ for alcohol ethoxylates (i.e., only ethoxy ethers, not propoxy ethers) with C = 9–18 and EO ≤ 21 (deemed to be generally representative of ranges in literature studies) is estimated at 0.4–198 µg L^−1^ (See online supplementary material Data 10a). Ranges of literature MEC are reported as <0.011–50.9 µg L^−1^ (Lara-Martin et al., 2011; McAvoy et al., 1998; See online Supplemental Data), and PEC_SW_ are reported as 0.06–16.76 µg L^−1^ (ECCC) 2019; See online Supplemental Data), again showing overlap, but with the values in our study ranging to one order of magnitude higher. However, as described above, the lack of information on specific polymer composition in this study limits the usefulness of these comparisons. For example, polymers such as steareth-20 included in the summed PEC_SW_ of our study will contain polymer chains of 20 and lower (within the ranges reported in literature studies), as well as higher chain lengths, which are not reported in literature studies.

A similar challenge exists for polyethers, with the only directly comparable literature data being those of Pauelsen et al. (2023), who reported PEG MECs (quantified independently of MW) up to 11 µg L^−1^ in surface water in Germany. This shows good agreement with total PEC_SW_ for all PEG polymers in our study, which is estimated as 0.6–30 µg L^−1^ (See online supplementary material Data 10a). For other reported literature MECs, general estimates can be made from our study for comparison with key polymer chain lengths (See online supplementary material Data 10a), but these are again limited by the lack of data on MW distributions, and thus there is likely significant overlap between polymers included and excluded in summed PEC_SW_ with those reported in the literature.

### Predicted environmental concentration in soil

Total PEC_SOIL_ estimates for whole polymer groups (See online supplementary material Data 9b) ranged from 0.02 mg kg^−1^ (polyquaterniums) to 39 mg kg^−1^ (polycarboxylates). Estimates of PEC_SOIL_ for individual polymers ranged from 9E-06 mg kg^−1^ (modified guar hydroxypropyltrimonium chloride, polyquaterniums group) to 15 mg kg ^−1^ (styrene/acrylates copolymer, polycarboxylates group; see online supplementary material Data 9c). The three polymers with the highest estimated PEC_SOIL_ were styrene/acrylates copolymer (0.1–15 mg kg^−1^), sodium polyacrylate (0.06–7 mg kg^−1^), and sodium acrylic acid/MA copolymer (0.06–6 mg kg^−1^), suggesting polycarboxylates are a key group in terms of environmental soil exposure to polymers. Members of the silicones and polyvinyl alcohol groups were also present in the top 10 polymers with the highest PEC_SOIL_, with dimethicone and PVOH having estimated PEC_SOIL_ of 0.9–4 and 0.5–3 mg kg^−1^, respectively.

Literature data for PEC_SOIL_ are scarce and even more limited than data for surface waters (See online supplementary material Data 10b). Measured environmental concentration data were available only for silicones, with concentrations of polydimethylsiloxane measured in sludge-amended agricultural soil ranging from < 0.41 to 10.4 mg kg^−1^ (Fendinger et al., 1997). These values are in good agreement with the sum of PEC_SOIL_ for dimethicone and simethicone determined in our study (1–4 mg kg^−1^). Literature PEC_SOIL_ for PAA (polycarboxylates group), reported to range from 0.47 to 4.37 mg kg^−1^ (HERA 2014a), is also in close agreement with estimates of our study (0.09–9 mg kg^−1^ for all PAA polymers; see online supplementary material Data 10b). Literature PEC_SOIL_ for PAA-MA (26.8–35.2 mg kg^−1^; HERA 2014b) is, however, higher than in our study (0.07–7 mg kg^−1^).

Literature PEC_SOIL_ for polyquaternium-68 has been reported at 0.0055–0.055 mg kg^−1^ (NICNAS 2009), showing overlap with the range for polyquaternium-68 in our study (0.00009–0.009 mg kg^−1^; see online supplementary material Data 10b), although ranging one order of magnitude higher. Comparison of data for alcohol alkoxylates and alcohol ethoxylate salts with literature data again remains challenging, as discussed previously, with broad comparisons suggesting close agreement for alcohol ethoxylates but not alcohol ethoxylate salts (See online supplementary material Data 10b) but further data on polymer mixtures and MW distributions being required for more in-depth comparisons.

### Biodegradation data

Several of the identified polymers are likely to further biodegrade in the environment (Table 3). Alcohol ethoxylates and alcohol ethoxylate salts of typical chain lengths are readily biodegradable (HERA 2004, 2009), and natural polymers such as starch are expected to rapidly biodegrade. However, modified natural polymers may be less susceptible to biodegradation; for example, carboxymethylcellulose (cellulose gum) is not readily biodegradable (Menzies et al., 2023), and hydroxyethylcellulose has been classed as nonbiodegradable (Bading et al., 2024), with these two polymers being the highest emitted in the cellulose group (See online supplementary material Data 8). Polypropylene glycol (PPG) and PEG (making up the majority of the polyethers group) are typically readily or inherently biodegradable (Beran et al., 2013; McDonough et al., 2023; Menzies et al., 2023; West et al., 2007), with low MW PEG and PPG degrading rapidly in river water (Zgoła-Grześkowiak et al., 2006). However, high MW polyethers (≥ 14.6 kDa) take significantly longer to degrade in ready tests (e.g., 86% biodegradation after 160 days for PEG of 500 kDa; Bernhard et al., 2008; Menzies et al., 2023), and biodegradation in marine water is significantly slower than in freshwater (Bernhard et al., 2008; West et al., 2007). Polyvinyl alcohol is also readily biodegradable (McDonough et al., 2023; Menzies et al., 2023) but with negligible biodegradation in marine water (Alonso-López et al., 2021).

**Table 3.** Summary of biodegradability for each key polymer group.

Polymer group	Biodegradability	Summary of biodegradation data	References
**Polycarboxylates**	Not readily biodegradable	Not readily biodegradable. Polyacrylic acid biodegradation (% CO_2_) in river water, river water and sediment, and soil: 7%–20% in 135 days, 12%–58% in 135 days, and 5%–35% in 165 days, respectively. Polyacrylic acid/maleic acid copolymer biodegradation (% CO_2_) in river water, river water and sediment, and soil: 12%–21% in 100 days, 11%–41% in 100 days, and 8%–32% in 165 days, respectively. Biodegradation decreases with increasing MW. Data are lacking for some key members of the group (e.g., styrene/acrylates copolymer).	Duis et al., 2021; HERA, 2014a, 2014b
**Alcohol ethoxylate salts**	Readily biodegradable	Readily biodegradable (C = 12–18, EO = 0–8).	HERA, 2004
**Alcohol alkoxylates**	Readily biodegradable	Readily biodegradable (C = 8–18, EO = 0–22). Further data may be required for high molecular weight (MW) members of the group (e.g., ceteareth-80).	HERA, 2009
**Polyol ethoxylate esters**	Not determined	Polysorbate 20 (CAS 9005-64-5) = readily biodegradable according to Registration, Evaluation, Authorisation and Restriction of Chemicals (REACH) dossier. Polysorbates 20, 60, 80, 61, and 65 have been degraded by bacteria isolated from soil and sediment; however, environmentally relevant studies are lacking, and no data are available for most group members (e.g., PEG-7 glyceryl cocoate, PEG-200 hydrogenated glyceryl palmate).	ECHA, 2020; Nguyen, 2018; Nguyen et al., 2021; Yeh & Pavlostathis, 2005
**Starch and derivatives**	Readily biodegradable	Natural polymers expected to rapidly biodegrade. CAS 68425-17-2 and 738602-93-2 readily biodegradable according to REACH dossiers.	ECHA, 2020
**Polyquaterniums**	Not readily biodegradable	Previously reviewed data by Duis et al. (2021): polyquaterniums-6, -10, and -16 are not readily biodegradable (< 10% ThOD in 28 days or not specified). Polyquaternium-6 is not inherently biodegradable. Polyquaterniums-7 and -16 may be inherently biodegradable (40%–50% DOC elimination for polyquaternium-16 in 28 days). Reliability of data not assignable due to lack of experimental details. Further data are required for these and other key polymers in the group (e.g., guar hydroxypropyltrimonium chloride).	Duis et al., 2021
**Polyethers and copolymers**	Readily or inherently biodegradable	Low MW PEG and PPG (and PEG/PPG copolymers) readily or inherently biodegradable (≤35 kDa, ≤14.6 kDa, or ≤1kDa depending on the study). Higher MW polyethers will biodegrade over longer time periods in ready tests (up to 86% biodegradation (% CO_2_) for PEG of 500 kDa in 160 days). Low MW PEG and PPG (<1 kDa) reach 99% biodegradation in river water in ≤17 days. Biodegradation of PEG and PPG in marine water is slow or negligible. More data required for other key group members (e.g., copolymer of PEG/vinyl acetate).	Beran et al., 2013; Bernhard et al., 2008; McDonough et al., 2023; Menzies et al., 2023; West et al., 2007; Zgoła-Grześkowiak et al., 2007; Zgoła-Grześkowiak et al., 2006
**Cellulose and derivatives**	Not readily biodegradable	Microcrystalline cellulose is readily biodegradable (82% biodegradation (% CO_2_) in 28 days). However, key modified cellulose polymers (contributing to 70% of the group emissions) are not readily biodegradable. Hydroxyethylcellulose has been previously assigned non-biodegradable (< 5% biodegradation (% CO_2_) in 28 days in ready test). Carboxymethylcellulose (cellulose gum) is not readily biodegradable (≤ 20% biodegradation (% CO_2_) in 28 days in ready test for DS = 0.6, 0.79, and 0.8, negligible biodegradation for DS = 1.2 in 60 days).	Bading et al., 2024; Menzies et al., 2023
**Polyvinyl alcohol**	Readily biodegradable	Readily biodegradable (MW 10,000 to 130,000 Da or not specified). Polyvinyl alcohol (PVOH; MW 9–10,000 Da) biodegrades in river water (71% (% CO_2_) in 90 days, 11%–79% ThOD in 180 days, variability due to variability in microbial communities obtained by grab-sampling). Negligible biodegradation in marine water.	Alonso-López et al., 2021; McDonough et al., 2023; Menzies et al., 2023
**Silicones**	Not biodegradable	Dimethicone has been classed as nonbiodegradable. Mineralization may eventually occur following soil-catalyzed hydrolysis (half-lives previously reviewed by Graiver et al., 2003; ≤28 days) and release of volatile compounds (predicted to oxidize in < 30 days).	Darracq et al., 2010; de Albuquerque Vita et al., 2023; Graiver et al., 2003

However, polycarboxylate polymers such as PAA and PAA-MA are not readily biodegradable (HERA 2014a, 2014b) and are slow to degrade in environmental matrices (Table 3), with biodegradation decreasing at higher MW. Similarly, polyquaterniums are likely to persist in the environment (although few data are available for these substances; Duis et al., 2021), and silicones are nonbiodegradable (e.g., Darracq et al., 2010; de Albuquerque Vita et al., 2023), although they may be removed by abiotic processes (reviewed by (Graiver et al., 2003). Polyol ethoxylate esters are generally lacking in data; a REACH registration dossier for polysorbate 20 (CAS 9005-64-5) states ready biodegradability (ECHA 2020), and bacterial strains isolated from environmental soil and sediment have been found to degrade polysorbates (Nguyen, 2018; Nguyen et al., 2021; Yeh & Pavlostathis, 2005), suggesting potential for biodegradation in the environment. However, environmentally relevant data are needed for other key members of the group to confirm this, particularly higher MW polymers (e.g., PEG-200 hydrogenated glyceryl palmate).

For readily biodegradable polymers, the PECs calculated in our study are likely to be reduced further from the point of release to the environment, and thus higher-tier modeling incorporating biodegradation may be useful to further refine PECs. However, it is worth noting that several of these biodegradable polymers have been detected in the environment (e.g., PEG has been detected in surface water at levels similar to PEC_SW_ of our study; Pauelsen et al., 2023). Furthermore, considerable environmental exposure to even biodegradable WSPs close to the point of release, before significant degradation has occurred, is likely and thus may be relevant in ERA.

### Ecotoxicity data

Literature ecotoxicity data were collated for the top 10 polymer groups with the highest emissions to identify data gaps and highlight potential polymers of concern (i.e., those polymers for which PEC_SW_ exceed concentrations which cause ecological effects).

For polycarboxylates, alcohol ethoxylate salts, and alcohol alkoxylates, extensive data were available and previously summarized in the HERA reports (HERA 2004, 2009, 2014a, 2014b; Table 4), with acute and chronic data for all standard species groups (fish, algae, and crustaceans). Chronic toxicity for alcohol ethoxylate salts and alcohol alkoxylates was reported to range to < 0.1 mg L^−1^, with upper estimates of PEC_SW_ for these groups of 0.7 and 0.4 mg L^−1^ (See online supplementary material Data 9a), respectively, although as noted previously, most group members are readily biodegradable, which is likely to reduce PEC_SW_. All recorded effect concentrations for polycarboxylates were > 1 mg L^−1^ (Table 4), with PEC_SW_ ranging to 0.9 mg L^−1^ for the entire group, suggesting a low potential for ecological hazard. However, some key polymers within the group lack ecotoxicity data, including styrene/acrylates copolymer.

**Table 4.** Summary of the available acute and chronic aquatic ecotoxicity data for each key polymer group.

Polymer group	Species group	Acute ecotoxicity (LC50 and EC50)/mg L^–1^ (durations)	Chronic ecotoxicity (EC10, NOEL, NOEC and LOEC)/mg L^–1^ (durations)	Other relevant ecotoxicity data/mg L^–1^ (durations)	References
**Polycarboxylates**	Fish	>100 – >10,000 (4 days)	100 (14–42 days)	—	HERA, 2014a, 2014b
	Algae	24.2 – >500 (3–4 days)	32–180 (3–4 days)	—	Hisar & Oehlmann, 2023; HERA, 2014a, 2014b
	Crustaceans	>100 – >1,000 (1–3 days)	3.75–450 (21 days)	—	HERA, 2014a, 2014b; Oliveira D’Alessandro et al., 2024
**Alcohol ethoxylate salts**	Fish	0.8–450 (6 hours–4 days)	0.1–1.7 (duration NR or 28–365 days)	LC50 = 0.1–0.94 (45 days)	Madsen et al., 2001; HERA, 2004; Little, 1991
	Algae	4–65 (duration NR or 2–3 days)	0.35–70 (duration NR or 3–21 days)	EC50 = 20–30 (21 days)	HERA, 2004; Madsen et al., 2001; Little, 1991
	Crustaceans	1.17 – >1000 (1–4 days)	0.06–16.5 (7–21 days)	—	HERA, 2004; Madsen et al., 2001; Little, 1991
**Alcohol alkoxylates**	Fish	0.4 – >100 (duration NR or 4 days)	0.079–8.983 (duration NR)	—	HERA, 2009
	Algae	0.05 – >990 (duration NR or 2–4 days)	0.030–9.791 (duration NR)	—	HERA, 2009
	Crustaceans	0.10–270 (duration NR or 1–2 days)	0.082–3.882 (duration NR)	EC/LC50 = 14 (10 days) EC10 = 0.2–28 (2 days)	HERA, 2009
**Polyol ethoxylate esters**	Fish	240 – >1,000 (1–2 days)	10,000 (duration NR)	*—*	Straub et al., 2014; Tsuji et al., 1986; Yarzhombek et al., 1991
	Algae	58.5 – ≥100 (duration NR)	3.16–10 (duration NR)	—	Straub et al., 2014
	Crustaceans	—	10—ca. 32 (21 days or duration NR)	EC50 = 100 (21 days) No ecotoxicity observed at 1–10 mg L^–1^ (2 and 21 days)	Brown et al., 1998; Straub et al., 2014
	Other	Insects: 8 %v/v (4 hours)	Worms: 2 (14 days)	Invertebrates: NOEC/LOEC = 0.002–0.01 % (2.9 days), no ecotoxic effect observed at 0.25% (4 days) Sea urchin embryo: NOEC = 21 – 50 (2.5–35 hours)	Bresch & Ockenfels, 1977; Chen et al., 2012; Kramer et al., 1983; Semenova et al., 2024; Wiger, 1985
**Starch and derivatives**	Fish	—	—	NR–ZERO = 5,000 (4 days)	Daugherty, 1951
	Algae	—	—	—	—
	Crustaceans	—	—	—	—
	Other	—	—	Mollusks: NR-ZERO = 1,000; NR-LETH = 3,000 (4 days)	Daugherty, 1951
**Polyquaterniums**	Fish	0.044 – >9,820 (1–4 days)	0.25–1.0 (30 days)	LC10 = 0.32–0.47 (1–2 days) NOEC = 0.037–600 (4 days) LOEC = 3.2 (4 days)	Biesinger & Stokes, 1986; Clifford et al., 2022; Cumming et al., 2008; Giltner & Baumann, 1991; Hall & Mirenda, 1991; Liber et al., 2005; Rawlings et al., 2022; Simões et al., 2021; Simões et al., 2022; Tooby et al., 1975; USEPA, 1992; Waller et al., 1993
	Algae	0.0088–682.8 (3–5 days)	< 0.001–31.2 (3–5 days)	LOEC = 0.02 mL/L (1 hour)	Cumming, 2008; Hansen et al., 2023; Hisar & Oehlmann, 2023; Jellyman et al., 2010; Pereira et al., 2018; Simões et al., 2021; Simões et al., 2022; USEPA, 1992
	Crustaceans	0.04 – 8,437 (2–4 days)	0.012–0.02 (21 days)	NOEL = 0.08 - <7.8 (2–4 days) NR-ZERO = >1 to <1.5 (2 days)	Biesinger & Stokes, 1986; Cowgill & Milazzo, 1991; Cumming, 2008; Giltner & Baumann, 1991; Hall & Mirenda, 1991; Pereira et al., 2018; Simões et al., 2021; Simões et al., 2022; USEPA, 1992
	Other	Mollusks: 0.35 – >60 (2 days) Plants: >0.65 – 1,060 (7–14 days) Marine bacteria: 208–977,619 (15 minutes or duration NR) Invertebrates: 0.17–484.9 (1 day) Insects: <6.25–>100 (2 days)	Plants: 0.043 (14 days)	Invertebrates: LOEC = 0.7-25 (1 hour) Mollusks: NOEL = 0.23 (2 days)	Biesinger & Stokes, 1986; Pereira et al., 2018; Simões et al., 2021; Simões et al., 2022; Srikanth & Berk, 1993; USEPA, 1992; Waller et al., 1993
**Polyethers and copolymers**	Fish	650–129,900 (1–4 days)	—	—	Bathe et al., 1975; Dawson et al., 1975; Harford et al., 2011; Pelka et al., 2017; Tsuji et al., 1986; Wildish, 1974
	Algae	18.51–>7,000 (3 days)	—	LC36 = 0.0125 (2 days) LC59 = 0.05 (2 days) No ecotoxicity observed up to 100 mg L^–1^ (2–3 days)	Harford et al., 2011; Hisar & Oehlmann, 2023; Kutt & Martin, 1974; Pastorino et al., 2022
	Crustaceans	1,170 (5–6 days)	—	No significant ecotoxicity observed at 0.025–0.1 mg L^–1^ (up to 22+ days)	Harford et al., 2011; Sandifer et al., 1975
	Other	Invertebrates: >7,000 (3 days) Plants: >7,000 (4 days)	—	Sea urchin embryo: NOEC = 1,200–2,260 (2.5–35 hours)	Harford et al., 2011; Semenova et al., 2024
**Cellulose and derivatives**	Fish	3,000–>20,000 (4 days)	—	No toxic effects at 58.29 and 100 mg L^–1^ (7 days)	Bathe et al., 1975; Kovacs et al., 2010; Souza et al., 2023
	Algae	—	—	No observed effect at 50 mg L^–1^ (4 days)	Schwab et al., 2011
	Crustaceans	87.2– >10,000 (2–4 days)	—	EC25 = >1,000–1,100 (7 days)	Kovacs et al., 2010; Oliveira D’Alessandro et al., 2024; Portmann & Wilson, 1971; Warne & Schifko, 1999
	Other	Plants: 2,244.2–2,532.7 (7 days) Insects: 58.29 and 100 (1 and 1.5 days)	—	—	Boros et al., 2022; Souza et al., 2023
**Polyvinyl alcohol**	Fish	> 1,000 (duration NR)	—	No significant effects observed in fish embryos from 0.001–1 mg L^–1^ (up to 5 days) No acute or chronic toxicity at 39.00–308.30 mg L^–1^ (4 days or duration NR)	Arfsten et al., 2004; McDonough et al., 2024; Nigro et al., 2022
	Algae	> 1,000 (3–4 days)	172.64–308.30 (duration NR)	—	Arfsten et al., 2004; McDonough et al., 2024
	Crustaceans	14.31–> 1,000 (1–4 days)	2.18–172.64 (duration NR)	No significant effects observed from 0.001–5.55 mg L^–1^ (10–28 days)	Arfsten et al., 2004; McDonough et al., 2024; Nigro et al., 2022
	Other	—	—	—	—
**Silicones**	Fish	3.16–>10,000 (duration egg gestation period or 3–7 days)	—	LC01 = 0.04 (7 days), NR-ZERO = 100 (4 days)	Birge et al., 1978; Hobbs et al., 1975
	Algae	—	—	—	—
	Crustaceans	44.5–> 1,000 (2–4 days)	—	LC01 = 379.6–600 (2 days), NR-ZERO = 100 (4 days) EC50 = >2,300–>88,900 mg kg^–1^ sediment (10 days) No effect up to 994 and 1,900 mg kg ^–1^ sediment (28 and 10 days)	Henry et al., 2001; Hobbs et al., 1975; Stevens et al., 2001
	Other	Amphibians: 6.95-–134.76 (duration egg gestation period or 4 days), mollusks: > 1,000 (4 days)	—	No significant toxic effects observed in invertebrates up to 10,000 mg kg^–1^ sediment (4 days) or up to 1,000 mg kg^–1^ sediment (28 days). No effect on plants up to 13 ppm in soil. No effect in insects up to 2,600 mg kg ^–1^ sediment (10–65 days)	Birge et al., 1978; Craig & Caunter, 1990; Henry et al., 2001; Hobbs et al., 1975; Tolle et al., 1995

*Note:* Data for polycarboxylates, alcohol ethoxylate salts, and alcohol alkoxylates were summarized for standard test species groups predominantly from previously collated data in the associated Human & Environmental Risk Assessment (HERA) reports (HERA 2004, 2009, 2014a, 2014b) and references therein. Data for the remaining polymer groups were summarized from data collated from the wider literature. Units are given in mg L^−1^ unless otherwise specified. LC50 = median lethal concentration; EC50 = median effect concentration; NOEL = no observed effect level; NOEC = no observed effect concentration; LOEC = lowest observed effect concentration.

Cellulose (and derivatives) and polyvinyl alcohol were the least toxic groups; although some data indicated moderate toxicity of carboxymethylcellulose (cellulose gum) and PVOH to crustaceans (acute half-maximal effect concentration [EC50] = 87.26 and chronic no observed effect concentration [NOEC] = 2.18 mg L^−1^, respectively; Arfsten et al., 2004; Warne & Schifko, 1999), these concentrations were orders of magnitude above maximum PEC_SW_ for the groups (0.06 and 0.019 mg L^−1^, respectively; see online supplementary material Data 9a). Silicones are also nontoxic; although some high toxicity has been reported with median lethal toxicity (LC50) values for fish as low as 3.16 mg L^−1^ (Birge et al., 1978), aquatic ecotoxicity tests are generally considered inapplicable to this group of substances due to their insolubility in water (Stevens, 1998), with more relevant sediment and soil tests indicating no toxicity even at high polymer concentrations (Craig & Caunter, 1990; Henry et al., 2001; Stevens et al., 2001; Tolle et al., 1995). Similarly, although few data were found for starch polymers, toxicity is highly unlikely for the natural polymers of this group.

Polyethers such as PEG are generally considered nontoxic, with literature ecotoxicity data typically indicating no or low toxicity (Table 4). Although moderate toxicity to algae has been observed for PEG 400, with acute EC50 values of 18.51 mg L^−1^ and 67.79 mg L^−1^ observed for *C. tenuissimus* and *P. tricornutum*, respectively (Pastorino et al., 2022), these values are orders of magnitude above PEC_SW_ for the entire polymer group (up to 0.09 mg L^−1^). However, Kutt and Martin (1974) reported 36% and 59% mortality after 2-day exposure of algae to 0.0125 and 0.05 mg L^−1^, respectively, of a PEG/PPG copolymer of MW 2,700 ± 300 g mol^−1^, suggesting high toxicity, which may be due to surfactant properties; maximum PEC_SW_ values for all PEG/PPG copolymers were estimated at 0.003 mg L^−1^ in our study, approximately an order of magnitude below these observed effects. Similarly, although acute data for polyol ethoxylate esters all indicated moderate or low toxicity (Table 4), chronic NOECs for algae and crustaceans for polysorbate 20 (3.16 and 10 mg L^−1^, respectively; Straub et al., 2014) indicated high toxicity, although again, these were higher than maximum PEC_SW_ for this polymer (0.058 mg L^−1^; see online supplementary material Data 9c). Key members of this group, including PEG-7 glyceryl cocoate and PEG-200 hydrogenated glyceryl palmate, had no ecotoxicity data available.

The polymer group with the highest ecotoxicity potential is undoubtedly polyquaterniums, with acute effects observed at concentrations < 1 mg L^−1^ for fish, algae, and crustaceans, indicating very high toxicity. However, none of the recorded effect concentrations for specific polymers were exceeded by PEC_SW_ of our study. For polyquaterniums -6, -16, -28, and -55, the lowest effect concentrations were 0.03, 0.12, 1.6, and 0.5 mg L^−1^, respectively, and corresponded to acute EC50 values for algae (polyquaternium-6 and polyquaternium-16) and fish (polyquaternium-28 and polyquaternium-55; Cumming, 2008; Cumming et al., 2008; Hansen et al., 2023). Maximum PEC_SW_ values for these polymers determined in our study were 0.001, 0.0001, 0.0002, and 0.0002 mg L^−1^, respectively (See online supplementary material Data 9c). For polyquaternium-10, the lowest effect concentration (acute algae EC50 of 0.04 mg L^−1^; Cumming, 2008) was closer to but still greater than maximum PEC_SW_ (0.01 mg L^−1^). No ecotoxicity data were found for polyquaternium-7 or guar hydroxypropyltrimonium chloride, the two highest emitted polymers in this group (maximum PEC_SW_ = 0.08 and 0.02 mg L^−1^, respectively; see online supplementary material Data 9c).

However, comparisons of PEC_SW_ for these polymers (and polymers in other groups) with ecotoxicity data are overall limited by a lack of information on MW and charge density in our study, which will affect ecological effects (e.g., Hansen et al., 2023; Rawlings et al., 2022). It should also be noted that predicted no-effect concentrations (PNECs) and environmental quality standards are typically 1–3 orders of magnitude lower than directly observed EC50s and NOECs due to application of assessment factors to account for uncertainty and ensure protection of the majority of species (European Commission 2011). Where PEC exceeds PNEC, this indicates unacceptable environmental risk. Thus, although no individual polymers were predicted to have PEC_SW_ above effect concentrations, this does not necessarily preclude risk. Determination of PNECs in our study was limited by most individual polymers lacking ecotoxicity data for all three standard species groups (European Commission 2011). Predicted no-effect concentrations are available for PAA, PAA-MA, alcohol ethoxysulfates and alcohol ethoxylates, polysorbate 20, polysorbate 80, and polyquaternium-67 (HERA 2004, 2009, 2014a, 2014b; Simões et al., 2021; 2022; Straub et al., 2014). The latter two of these polymers were not identified in our study, and comparison of PNEC values to PEC_SW_ for the remaining polymers was again limited by a lack of structural information such as MW. Direct comparison was only possible for PAA and PAA-MA (because literature PNECs accounted for a range of MW) and polysorbate 20 (because the structure and MW of this polymer is well defined). The relevant PNEC and PEC_SW_ values for these polymers are shown in Table 5. Although PEC_SW_ values for both polycarboxylate polymers are significantly lower than their PNECs, for polysorbate 20, maximum PEC_SW_ (58 µg L^−1^) is similar to the aquatic PNEC (63 µg L^−1^; Straub et al., 2014), suggesting polyol ethoxylate esters may be a priority for further risk assessment; information on removal in WWT for this group may thus be useful to refine PEC_SW_. In addition, PNECs for various polyquaternium-67 polymers with differing charge densities and hydrophobic modifications range to as low as 0.17 µg L^−1^ (Simões et al., 2021; 2022). Because this polymer is less toxic than other polyquaternium polymers discussed above (Cumming, 2008; Cumming et al., 2008; Hansen et al., 2023), there is significant potential for the identified polyquaterniums to exceed safe concentrations in the environment (e.g., maximum PEC_SW_ for polyquaternium-6, which is more toxic than polyquaternium-67 based on available data, is 1.3 µg L^−1^), although polyquaternium toxicity may also be mitigated by the presence of humic acid and suspended solids in the environment (e.g., Hansen et al., 2023; Rawlings et al., 2022). Predicted no-effect concentrations for individual high-emission polymers across all groups, requiring extensive ecotoxicity data across multiple species, are needed to characterize potential risk. In addition, the potential for some polymers to contribute to ecological effects as a mixture (e.g., polyquaterniums, for which ecotoxicity is directly related to cationic charge; Connors et al., 2023) may be significant for ERA.

**Table 5.** Comparison of surface water exposure (PEC_SW_) of the present study with aquatic predicted no-effect concentrations (PNECs) from the literature (all µg L^–^^1^), for individual polymers with sufficient information for direct comparisons to be made.

Polymer	PEC_SW_ (present study)	Aquatic PNEC (literature)	Reference
**Polyacrylic acid**	0.5–222	1,200	HERA, 2014a
**Polyacrylic acid/maleic acid copolymer**	0.4–166	560	HERA, 2014b
**Polysorbate 20**	12–58	63	Straub et al., 2014

### Knowledge gaps and future applications

The availability of key data for the top three polymers (highest M_DTT_) in each of the top 10 highest-emitted groups is summarized in Table 6. These data have been compiled and discussed in the above sections. This provides a list of 30 key polymers, based on down-the-drain emissions, which can be prioritized for further study based on associated data gaps (Table 6). Several polymers have associated REACH registration dossiers, although many have multiple CAS numbers with different levels of information available (ECHA 2020).

**Table 6.** Summary of the availability of data and key knowledge gaps for the top three polymers (by relative emissions) in each of the 10 polymer groups with highest down-the-drain emissions (M_DTT_).

Group	Polymer	Contribution to group (%)	REACH dossier (CAS)^a^	WWT removal	MEC (water)	**MEC (soil)**	Biodegradability	Ecotoxicity
Alcohol ethoxylate salts	Sodium laureth sulfate	70.1	68891-38-3	✓	✓	✗	✓	✓
	MEA-laureth sulfate	15.7	157627-92-4	✓	✓	✗	✓	✓
	Sodium C12-15 pareth sulfate	4.9	✗	✓	✓	✗	✓	✓
Alcohol alkoxylates	Laureth-4	14.2	68439-50-9; 9002-92-0	✓	✓	✗	✓	✓
	PEG/PPG-10/2 propylheptyl ether	9.2	✗	✗	✗	✗	✗	✗
	C11-15 Pareth-7	7.2	68131-40-8	✓	✓	✗	✓	✓
Polycarboxylates	Styrene/acrylates copolymer	38.2	✗	✗	✗	✗	✗	✗
	Sodium polyacrylate	17.5	9003-01-4	✓	✗	✗	✓	✓
	Sodium acrylic acid/MA copolymer	16.0	✗	✓	✗	✗	✓	✓
Polyol ethoxylate esters	PEG-7 glyceryl cocoate	29.4	✗	✗	✗	✗	✗	✗
	PEG-200 hydrogenated glyceryl palmate	22.0	✗	✗	✗	✗	✗	✗
	Polysorbate 20	17.2	9005-64-5	✗	✗	✗	✓	✓
Polyethers and copolymers	PPG-26	23.2	25322-69-4	✓	✓^b^	✗	✓	✗
	Copolymer of PEG/vinyl acetate	11.5	✗	✗	✗	✗	✗	✗
	Polyethylene glycol	10.0	25322-68-3	✓	✓	✗	✓	✓
Starch and derivatives	Dextrin	51.6	✗	✗	✗	✗	✓	✗
	Oryza sativa (rice) starch	18.7	✗	✗	✗	✗	✓	✗
	Hydrogenated starch hydrolysate	8.1	68425-17-2; 738602-93-2	✗	✗	✗	✓	✗
Silicones	Dimethicone	49.3	✗	✓	✓	✓	✓	✓
	Dimethiconol	14.4	✗	✗	✗	✗	✓	✗
	Trimethylsiloxysilicate	7.5	✗	✗	✗	✗	✗	✗
Polyquaterniums	Polyquaternium-7	59.2	✗	✗	✗	✗	✗^c^	✗
	Guar hydroxypropyltrimonium chloride	17.5	✗	✗	✗	✗	✗	✗
	Polyquaternium-10	8.0	✗	✓	✗	✗	✗^c^	✓
Polyvinyl alcohol	Polyvinyl alcohol	96.1	✗	✓	✗	✗	✓	✓
	Polyvinyl alcohol film	2.0	✗	✓^d^	✗	✗	✓^d^	✓^d^
	Thermal shrinkable PVOH film	2.0	✗	✓^d^	✗	✗	✓^d^	✓^d^
Cellulose and derivatives	Hydroxyethyl cellulose	39.5	✗	✗	✗	✗	✓	✓
	Cellulose gum	30.5	✗	✓	✗	✗	✓	✓
	Microcrystalline cellulose	16.7	✗	✗	✗	✗	✓	✓

Note: Summary includes wastewater treatment (WWT) removal data, measured environmental concentration (MEC) data, biodegradability data, and ecotoxicity data compiled and discussed in the present study. Ticks (✓) indicate that data are available, and crosses (✗) indicate data gaps.

a Many polymers correspond to multiple CAS numbers, and often only some of these CAS numbers have corresponding REACH registration dossiers; therefore for any polymers which do have one or multiple REACH registration dossiers, the CAS numbers corresponding to these dossiers are listed. These were determined from the ECHA Information on Chemicals database (ECHA, 2020).

b MEC concentrations in water are only available for PPG of lower chain lengths (than PPG-26).

c Some limited data are available (Duis et al., 2021), however insufficient experimental details mean further data are required.

d Assuming that polyvinyl alcohol film and thermal shrinkable polyvinyl alcohol film contain only polyvinyl alcohol.

From Table 6, several polymers and polymer types can be identified as lacking in key data. Polyol ethoxylate esters as a group are severely lacking both environmental fate and effects data, and more research is thus warranted to determine similarities and differences between these and other polymeric nonionic surfactants, such as alcohol ethoxylates, particularly given the closeness of PEC_SW_ (this study) to PNEC (Straub et al., 2014) for polysorbate 20. In addition, the most highly emitted polyquaterniums are lacking in data, which is highly significant given the potential ecotoxicity and persistence of cationic polymers. Styrene/acrylates copolymer is also likely to require further research moving forwards, because it differs significantly to other polycarboxylates, is the highest-emitted polymer in this group (as well as one of the highest-emitted polymers overall), and does not have any corresponding fate or effects data. Most of these criteria also apply to copolymers of PEG/vinyl acetate (polyethers group). Several polymers identified in our study were highlighted by Pecquet et al. (2019) as having insufficient data available for conducting an ERA, including polyquaternium-10, polyquaternium-7, and styrene/acrylates copolymer; we have here shown high emission rates and therefore high potential for environmental exposure to these polymers, further suggesting they should be prioritized for further study. In addition, Pecquet et al. (2019) excluded polymers identified only by trade names and those lacking in CAS numbers from their dataset due to inadequate characterization. In our study, polymers were identified based on names listed in product ingredients, which are often more informative than CAS numbers (although, as discussed previously, information on polymer structure and MW was lacking in most cases). Therefore, although data of the present study may incorporate some materials that do not strictly fit the OECD polymer definition, there is also potential for inclusion of other polymers that do not have sufficient data for ERA but were excluded from analyses by Pecquet et al. (2019). Data are also somewhat lacking for cellulose and starch polymers; as these are naturally occurring, they may be of less cause for concern, although physical effects from release of large quantities of natural polymers should not be overlooked, and the assumption that these polymers cause minimal ecological effects should be confirmed for chemically modified variants.

Several assumptions were needed to account for a lack of data for most identified polymers when obtaining estimates of emissions and PEC in the present study. Although most polymer groups had some level of WWT removal data available to calculate PEC, this was often not specific to the highest contributing group members, and thus, further data are needed to refine estimates and the need for subgroups and obtain more specific PEC estimates for each polymer. For most polymers identified, including 23 of these top 30 polymers, MW data were not available, which further limits the ability to fully characterize polymers and their fate. Polymer naming conventions are also oftentimes ambiguous and for many polymers, do not reveal sufficient information on polymer structure. Increased transparency in reporting of key polymer properties for polymers in current use, particularly relating to MW, would significantly enhance further data collection and ERA. Although reporting of full MW distribution and mixture composition data may be unrealistic currently, even reporting of average MW would greatly facilitate risk assessment efforts and allow more specific analyses of polymers based on their individual properties, including ecotoxicity, WWT removal, and environmental biodegradation, which are all MW-dependent. Although the polymer groups established in this study are also a useful first step to indicate key polymer functionalities that are likely released to the environment, particularly in light of the severe lack of emissions data for most WSPs, many of these groups contain a broad range of polymers with (potentially) different MWs, additional monomer units (in the case of identified copolymers), and physicochemical properties (e.g., charge density), with insufficient information to determine these properties, in most cases, from available product ingredients data. For higher-tier exposure and effects assessment, it may be useful to test the extent to which these differences in polymer properties affect behavior, ecotoxicity, and subsequent environmental risk. This is likely to lead to the need for further refinement of groups and subgroups as more data become available. However, for some groups, determination of grouped PEC may be more relevant; for example, in the alcohol alkoxylates group, polymers such as “C11-15 pareth-7”, “C12-14 pareth-7”, and “C12-14 pareth-n” are listed under separate names but will contain many of the same components, and chain lengths outside this range will also exist as a distribution, indicating significant overlap between group members.

Despite the uncertainty in emissions and PEC data due to the assumptions applied, the data in this study are useful in providing both preliminary exposure estimates for a group of substances for which data are severely lacking and in prioritizing polymers for further research to fill these data gaps with more robust methods. Environmental concentrations have been modeled for initially unidentified polymers without the need for substance-specific usage or emissions data, such as manufacture and import volumes. The approach used allows identification of specific polymers without prior knowledge of polymer identities, meaning the full range of polymers used in the incorporated products can be accounted for. The down-the-drain emissions estimates may be useful for future exposure assessments and modeling, particularly in combination with addressing the knowledge gaps highlighted for key high-emission polymers in Table 6, with incorporation of exposure-based indicators into prioritization approaches having been recommended previously (Groh et al., 2023). As more data become available on identities and environmental fate behavior of these polymers and identified knowledge gaps are addressed, more complex models could be developed, combining emissions estimates of our study with fate and biodegradability data to refine PECs. Incorporation of other product types which may be released down-the-drain from household use, including toothpaste, moisturizer, fabric conditioner, deodorant, and others, may also be significant for future polymer identification and emissions estimates.

## Conclusion

Results from the emissions modeling approach developed in this study suggest that a wide variety of WSPs found in household products are likely to be present in the environment. Several high-emission polymers are currently lacking in environmental data, including polymers from the polycarboxylates, polyethers, polyol ethoxylate esters, and polyquaternium groups, and further research is recommended to fill these data gaps by characterizing environmental fate and effects as well as the suitability of read-across approaches. Several polymers identified in this study have been detected in the environment already, and development of analytical methods to characterize other polymer types is critical. However, analyses in this study were hindered by a lack of reporting of key polymer properties and ambiguity in polymer naming conventions. Increased transparency in the identities of polymers used in industry as well as better characterization methods will greatly facilitate future research.

## Supplementary Material

vgae030_Supplementary_Data

## Data Availability

All data except raw ingredients and brand data are included in the supplemental data. The aforementioned additional data are available on request from the authors.
