# Supplementary material for: Emissions of water-soluble polymers from household products to the environment: a prioritization study
Source: Environ Toxicol Chem. 2025 Jan 6;44(2):563–88. doi: 10.1093/etojnl/vgae030 (PMC11816303; doi:10.1093/etojnl/vgae030)
Supplement: vgae030_Supplementary_Data [file vgae030_supplementary_data.pdf]

EMISSIONS OF WATER-SOLUBLE POLYMERS FROM HOUSEHOLD PRODUCTS TO THE ENVIRONMENT: A PRIORITISATION STUDY

Supplemental Data

Contents

1. Supermarket websites 4
2. Product brands 4
3. Potential polymers excluded from the dataset 5
4. Polymer concentration in products 6
 4a. Laundry detergent....................6
 4b. Machine dishwashing detergent....................7
 4c. Hand dishwashing detergent....................7
 4d. Toilet cleaner and bleach....................7
 4e. Bodywash 8
 4f. Handwash....................9
 4g. Soap bars 9
 4h. Bath liquid....................10
 4i. Shampoo....................11
 4j. Conditioner....................12
5. Market penetration....................13
 5a. Laundry detergent....................13
 5b. Machine dishwashing detergent....................14
 5c. Hand dishwashing detergent....................14
 5d. Toilet cleaner and bleach....................14
 5e. Bodywash 15
 5f. Handwash....................15
 5g. Soap bars 16
 5h. Bath liquid....................16
 5i. Shampoo....................17
 5j. Conditioner....................18
6. Product usage....................19
7. Down-the-drain emissions of polymer groups....................20

8. Polymer grouping and individual emissions....21
9. Predicted environmental concentration....30
9a. Polymer groups in surface water....30
9b. Polymer groups in soil....30
9c. Individual polymers in surface water and soil....31
10. Comparison to exposure data from the literature....37
10a. Surface water....37
10b. Soil....51
References....56

1. Supermarket websites

Supplemental Data 1: Supermarket websites used for identification of household product types released down-the-drain at point-of-use, and major brands for each product type, for the UK.

Supermarket	Link	Dates accessed
Tesco	https://www.tesco.com/	April 2020-January 2021
Sainsbury's	https://www.sainsburys.co.uk/	April 2020-January 2021
Asda	https://www.asda.com/	April 2020-January 2021
Morrisons	https://groceries.morrisons.com/	April 2020-January 2021

2. Product brands

Supplemental Data 2: Total numbers of brands included in the final dataset for each product type.

Product type	Number of brands included in the final study
Laundry detergent	9
Dishwashing detergent (machine)	4
Dishwashing detergent (hand)	3
Toilet cleaners (including toilet cleaning liquid, bleach, and disinfectant)	9
Shampoo	21
Conditioner	19
Bodywash	22
Handwash	15
Soap bars	14
Bath liquid	13

3. Potential polymers excluded from the dataset

Supplemental Data 3: Potential polymers identified from product ingredients that have been excluded from the dataset due to insufficient information.

Name	Reason for exclusion
1,4-Benzenedicarboxylic acid, 1,4-dimethyl ester, polymer:1,	Insufficient information /incomplete name does not allow polymer identification for gathering of information from patents
Peptides, salts, sugars from fermentation (process)	Mixture of polymer (peptides) and non-polymers, with insufficient information to determine composition
Poly(oxy-1,2-ethanediyl), alpha-(1-oxohexadecyl)-omega-hydroxy-	Insufficient information /incomplete name does not allow polymer identification for gathering of information from patents
Polymers	Insufficient information from name does not allow polymer identification for gathering of information from patents
Sulfonated polymer	Insufficient information from name does not allow polymer identification for gathering of information from patents

4. Polymer concentration in products

4a. Laundry detergent

Supplemental Data 4a: Estimated polymer concentration in product (F_{pol}) of polymer groups for laundry detergent, with selection of patents (corresponding to final concentration estimates) referenced. Note that polymers grouped as 'Other' are treated separately, as individual polymers. Groups/polymers are listed in order of highest maximum F_{pol} for this product type.

Groups/polymers	Min. F_{pol}	Max. F_{pol}	References (patents)
Alcohol ethoxylate salts	0.14	0.23	Hsu *et al.* 2006b; Della Noce 2016
Polycarboxylates	0.02	0.2	Machin and van de Pas 1992; Reyes 2011; Gori and Baltsen 2016
Alcohol alkoxylates	0.09	0.15	Arisandy *et al.* 2014
Lignin*	0.004	0.11	Batchelor and Bird 2015
Polyesters	0.02	0.1	Bennett *et al.* 2012
Hemicelluose*	0.005	0.1	Hüffer *et al.* 2016
Polyvinyl alcohol	0.01	0.06	Antwerpen *et al.* 1994
Starch and derivatives	0.03	0.06	Desforges 1972; Temple *et al.* 1978; Casteel *et al.* 2001
Fatty acid ethoxylates	0.01	0.05	Hsu *et al.* 2006a
Poly(oxy)alkylene terephthalates	0.01	0.05	Beagle *et al.* 1999
Cellulose and derivatives	0.01	0.03	Leupin and Gosselink 2002; Wang *et al.* 2003
Ethoxylated polyethyleneimines	0.003	0.03	Souter *et al.* 2006; Borne 2012
Silicones	0.01	0.03	Zhen and Strickland 1998; Depoot *et al.* 2003; Zhu and Hsu 2004
Polyvinylpyridine-N-oxide*	0.002	0.025	Meine and Bessler 2015
Plant gums	0.001	0.02	Corominas *et al.* 2013
Polyethers and copolymers	0.003	0.02	Jones 1984; Kud *et al.* 1987
Proteins/polypeptides	0.0001	0.02	Gorlin *et al.* 2008
Vinylimidazole/ vinylpyrrolidone homo- and co-polymers	0.005	0.02	Detering *et al.* 1997; Gopalkrishnan and Guiney 1999
Ethoxylated m-toluidine*	0.000033	0.0165	Fernandes *et al.* 2013
Polymerised aromatic sulfonate salts	0.005	0.01	McDonald 1966; Garcia *et al.* 1998; Moeller *et al.* 2002
Polyquaterniums	0.0005	0.002	Boutique *et al.* 2008
Polymeric colourants	0.00015	0.0015	Schramm *et al.* 2005
Amine/formaldehyde polymers	0.000075	0.0006	Fossum *et al.* 2007; Ohtani and Azuma 2016

*Individual polymer, group 'Other'

4b. Machine dishwashing detergent

Supplemental Data 4b: Estimated polymer concentration in product (F_{pol}) of polymer groups for machine dishwashing detergent, with selection of patents (corresponding to final concentration estimates) referenced. Note that polymers grouped as 'Other' are treated separately, as individual polymers. Groups/polymers are listed in order of highest maximum F_{pol} for this product type.

Groups/polymers	Min. F_{pol}	Max. F_{pol}	References (patents)
Starch and derivatives	0.001	0.2	Saito and Takada 2016
Alcohol alkoxylates	0.01	0.1	Fischer *et al.* 2012
Polycarboxylates	0.01	0.1	Sabatelli and Brungs 1971; Weber *et al.* 2012
Alginic acid*	0.005	0.1	Chun *et al.* 1992
Polyethers and copolymers	0.0075	0.06	Manske 2004
Polyvinyl alcohol	0.01	0.06	Antwerpen *et al.* 1994
Polyquaterniums	0.0025	0.04	Parran 1970; Eiting *et al.* 2016
Plant gums	0.0125	0.025	Fox *et al.* 1981
Silicones	0.01	0.018	Charles 2014
Cellulose and derivatives	0.001	0.005	Gomez Ruiz *et al.* 2013

*Individual polymer, group 'Other'

4c. Hand dishwashing detergent

Supplemental Data 4c: Estimated polymer concentration in product (F_{pol}) of polymer groups for hand dishwashing detergent, with selection of patents (corresponding to final concentration estimates) referenced. Groups are listed in order of highest maximum F_{pol} for this product type.

Groups	Min. F_{pol}	Max. F_{pol}	References (patents)
Alcohol ethoxylate salts	0.15	0.25	Moffatt 1995
Alcohol alkoxylates	0.03	0.2	Evers and Maddox 2014
Polyethers and copolymers	0.0075	0.06	Manske 2004
Polyethylenimine ethoxylates and polyether copolymers	0.002	0.015	Borne 2012
Polyquaterniums	0.0005	0.01	Perez-Prat Vinuesa *et al.* 2014

4d. Toilet cleaner and bleach

Supplemental Data 4d: Estimated polymer concentration in product (F_{pol}) of polymer groups for toilet cleaner and bleach, with selection of patents (corresponding to final concentration estimates) referenced. Groups are listed in order of highest maximum F_{pol} for this product type.

Groups	Min. F_{pol}	Max. F_{pol}	References (patents)
Alcohol alkoxylates	0.01	0.07	Klinkhammer *et al.* 2004
Cellulose and derivatives	0.00001	0.05	Cheung and Costa 2003
Silicones	0.0001	0.05	Cermenati and Tomarchio 2006
Alcohol ethoxylate salts	0.005	0.02	Baixas Veiga *et al.* 1994
Plant gums	0.002	0.006	Miskiel and Solanki 1999
Polymeric colourants	0.000001	0.001	Marin and Bergstrom 2013

4e. Bodywash

Supplemental Data 4e: Estimated polymer concentration in product (F_{pol}) of polymer groups for bodywash, with selection of patents (corresponding to final concentration estimates) referenced. Note that polymers grouped as 'Other' are treated separately, as individual polymers. Groups/polymers are listed in order of highest maximum F_{pol} for this product type.

**Groups/polymers**	**Min. F_{pol}**	**Max. F_{pol}**	**References (patents)**
Alcohol alkoxylates	0.08	0.25	Rosser 1990
Polyolefins	0.05	0.25	Dixon *et al.* 2000; Glenn *et al.* 2000; Gittleman *et al.* 2015
Polymerised aromatic sulfonate salts	0.05	0.2	Taylor *et al.* 2002; Seitz *et al.* 2005
Proteins/polypeptides	0.005	0.2	Staples 1982; Giddey *et al.* 1991
Alcohol ethoxylate salts	0.03	0.1	Malik *et al.* 1987
Polyol ethoxylate esters	0.02	0.1	Sebillotte-Arnaud and Guillou 2002
Polyurethane Crosspolymer-2*	0.005	0.1	Noor and Lemma 2007; Yu *et al.* 2009; Hourigan *et al.* 2015
Disodium Laureth Sulfosuccinate*	0.015	0.07	Fan *et al.* 2008
Cellulose and derivatives	0.01	0.06	Conklin 1991
Fatty acid ethoxylates	0.01	0.05	Zofchak *et al.* 2006
Polycarboxylates	0.005	0.05	Margosiak *et al.* 2009
Polyglyceryl esters and polyglycerin	0.0025	0.05	Fevola 2012
Starch and derivatives	0.02	0.04	Yang and Tsaur 2012
Hydrolysed proteins and derivatives	0.001	0.03	Giddey *et al.* 1991; Fan *et al.* 2014
Polyethers and copolymers	0.001	0.03	Oldenhove *et al.* 2011; Fan *et al.* 2014
Polyquaterniums	0.001	0.03	Tsaur 2012; Fan *et al.* 2014
PEG-4 Rapeseedamide*	0.005	0.03	Librizzi 2002
Plant gums	0.001	0.02	Tsaur and Aronson 2003; Merces 2013
Butyl Acrylate/Ethyltrimonium Chloride Methacrylate/Styrene Copolymer*	0.001	0.015	Mabille and Leroy 2010

*Individual polymer, group 'Other'

4f. Handwash

Supplemental Data 4f: Estimated polymer concentration in product (F_{pol}) of polymer groups for handwash, with selection of patents (corresponding to final concentration estimates) referenced. Note that polymers grouped as 'Other' are treated separately, as individual polymers. Groups/polymers are listed in order of highest maximum F_{pol} for this product type.

Groups/polymers	Min. F_{pol}	Max. F_{pol}	References (patents)
Alcohol alkoxylates	0.08	0.25	Rosser 1990
Proteins/polypeptides	0.005	0.2	Staples 1982; Giddey *et al.* 1991
Alcohol ethoxylate salts	0.03	0.1	Malik *et al.* 1987
Polyol ethoxylate esters	0.02	0.1	Sebillotte-Arnaud and Guillou 2002
Fatty acid ethoxylates	0.01	0.05	Zofchak *et al.* 2006
Polycarboxylates	0.005	0.05	Margosiak *et al.* 2009
Polyglyceryl esters and polyglycerin	0.0025	0.05	Fevola 2012
Starch and derivatives	0.02	0.04	Yang and Tsaur 2012
Hydrolysed proteins and derivatives	0.001	0.03	Giddey *et al.* 1991; Fan *et al.* 2014
Polyethers and copolymers	0.001	0.03	Oldenhove *et al.* 2011; Fan *et al.* 2014
Polyquaterniums	0.001	0.03	Tsaur 2012; Fan *et al.* 2014
PEG-4 Rapeseedamide*	0.005	0.03	Librizzi 2002
Plant gums	0.001	0.02	Tsaur and Aronson 2003; Merces 2013

*Individual polymer, group 'Other'

4g. Soap bars

Supplemental Data 4g: Estimated polymer concentration in product (F_{pol}) of polymer groups for soap bars, with selection of patents (corresponding to final concentration estimates) referenced. Note that polymers grouped as 'Other' are treated separately, as individual polymers. Groups/polymers are listed in order of highest maximum F_{pol} for this product type.

Groups/polymers	Min. F_{pol}	Max. F_{pol}	References (patents)
Alcohol alkoxylates	0.05	0.15	Wis-Surel and Moaddel 2013; Pan *et al.* 2014
Alcohol ethoxylate salts	0.05	0.15	Potgeiter *et al.* 1999; Pan *et al.* 2014
Cationic silicones	0.005	0.1	Schmucker-Castner *et al.* 2005; Seidling and Cunningham 2014
Hydroxypropyl Cyclodextrin*	0.01	0.1	Salvador *et al.* 2011
Starch and derivatives	0.02	0.05	Thiessies *et al.* 2013; Astolfi *et al.* 2017
Silicones	0.001	0.045	Payne and Chupa 1999
Polyethers and copolymers	0.01	0.02	Demson and Dalton 2004

*Individual polymer, group 'Other'

4h. Bath liquid

Supplemental Data 4h: Estimated polymer concentration in product (F_{pol}) of polymer groups for bath liquid, with selection of patents (corresponding to final concentration estimates) referenced. Note that polymers grouped as 'Other' are treated separately, as individual polymers. Groups/polymers are listed in order of highest maximum F_{pol} for this product type.

**Groups/polymers**	**Min. F_{pol}**	**Max. F_{pol}**	**References (patents)**
Alcohol alkoxylates	0.08	0.25	Rosser 1990
Proteins/polypeptides	0.005	0.2	Staples 1982; Giddey *et al.* 1991
Alcohol ethoxylate salts	0.03	0.1	Malik *et al.* 1987
Polyol ethoxylate esters	0.02	0.1	Sebillotte-Arnaud and Guillou 2002
Disodium Laureth Sulfosuccinate*	0.015	0.07	Fan *et al.* 2008
Fatty acid ethoxylates	0.01	0.05	Zofchak *et al.* 2006
Polyethers and copolymers	0.01	0.05	Ribery and Penverne 2003; Oldenhove *et al.* 2011
Polycarboxylates	0.005	0.05	Margosiak *et al.* 2009
Polyquaterniums	0.001	0.03	Tsaur 2012; Fan *et al.* 2014
PEG-4 Rapeseedamide*	0.005	0.03	Librizzi 2002
Plant gums	0.001	0.02	Tsaur and Aronson 2003; Merces 2013

*Individual polymer, group 'Other'

4i. Shampoo

Supplemental Data 4i: Estimated polymer concentration in product (F_{pol}) of polymer groups for shampoo, with selection of patents (corresponding to final concentration estimates) referenced. Note that polymers grouped as 'Other' are treated separately, as individual polymers. Groups/polymers are listed in order of highest maximum F_{pol} for this product type.

**Groups/polymers**	**Min. F_{pol}**	**Max. F_{pol}**	**References (patents)**
Alcohol ethoxylate salts	0.05	0.25	Janchitraponvej and Brown 1995
Polymerised aromatic sulfonate salts	0.05	0.25	Seitz *et al.* 2005
Laureth-5 Carboxylic Acid*	0.05	0.25	Janchitraponvej and Brown 1995
Disodium Laureth Sulfosuccinate*	0.12	0.2	Hilvert and Winstel 2014
Polyethers and copolymers	0.025	0.125	Takebayashi and Ishiwatari 1999
Polyol ethoxylate esters	0.02	0.1	Sebillotte-Arnaud and Guillou 2002
Methyl Gluceth-10*	0.005	0.08	Bergmann 1994
Alcohol alkoxylates	0.025	0.05	Preston 1986
Hydrolysed protein and derivatives	0.0005	0.05	Hippe *et al.* 2012; Gerardi *et al.* 2014; Patron and Ditschun 2017
Polyesters	0.001	0.05	Barrios *et al.* 2008
Polyglyceryl esters and polyglycerin	0.005	0.05	Zhou *et al.* 2017
Silicones	0.005	0.05	Bolich and Williams 1988
Starch and derivatives	0.003	0.05	Coffindaffer and Schrader 1998
Vinylimidazole/vinylpyrrolidone homo- and co-polymers	0.003	0.05	Coffindaffer and Schrader 1998
Polycarboxylates	0.009	0.04	Holt and Shaw 2006
Silicone alkoxylates	0.005	0.02	Mabille and Leroy 2010
Cationic silicones	0.005	0.02	Mabille and Leroy 2010
Cellulose and derivatives	0.001	0.02	Hirota and Takaya 1986
Poly(Linseed Oil)*	0.005	0.02	Thiel *et al.* 1994
Fatty acid ethoxylates	0.015	0.015	Yu 2015
Polyquaterniums	0.001	0.015	Mabille and Leroy 2010
Plant gums	0.0025	0.01	Janchitraponvej and Brown 1995
Proteins/polypeptides	0.00001	0.01	Kelly and Roddick-Lanzilotta 2004
Sodium Hyaluronate*	0.0009	0.01	Dos Santos 2017

*Individual polymer, group 'Other'

4j. Conditioner

Supplemental Data 4j: Estimated polymer concentration in product (F_{pol}) of polymer groups for conditioner, with selection of patents (corresponding to final concentration estimates) referenced. Note that polymers grouped as 'Other' are treated separately, as individual polymers. Groups/polymers are listed in order of highest maximum F_{pol} for this product type.

Groups/polymers	Min. F_{pol}	Max. F_{pol}	References (patents)
Polymerised aromatic sulfonate salts	0.05	0.25	Seitz *et al.* 2005
Alcohol ethoxylate salts	0.07	0.15	Jordan 2013
Polyglyceryl esters and polyglycerin	0.01	0.15	Carew *et al.* 2003
Cellulose and derivatives	0.01	0.06	Conklin 1991
Starch and derivatives	0.05	0.06	Gevgilili and Liang 2017
Alcohol alkoxylates	0.01	0.05	Grit *et al.* 2006
Fatty acid ethoxylates	0.011	0.05	Hindley 2016
Hydrolysed protein and derivatives	0.0005	0.05	Hippe *et al.* 2012; Gerardi *et al.* 2014; Patron and Ditschun 2017
Plant gums	0.005	0.05	Zofchak and Carson 2005
Polyethers and copolymers	0.005	0.05	Deryon *et al.* 2013
Polyesters	0.001	0.05	Barrios *et al.* 2008
Silicones	0.001	0.05	Sturla *et al.* 2013
Vinylimidazole/vinylpyrrolidone homo- and co-polymers	0.005	0.05	Shih *et al.* 1992
Poly(Linseed Oil)*	0.005	0.05	Meralli *et al.* 2014
Polycarboxylates	0.001	0.04	Quenzer 2000
Silicone alkoxylates	0.001	0.03	Molenda and Tietjen 2017
Cationic silicones	0.005	0.03	Read and Southey 2015
PPG-3 Benzyl Ether Myristate*	0.01	0.03	Demitz *et al.* 2009
Polyquaterniums	0.003	0.025	Hoffmann and Ning 2016
Polyol ethoxylate esters	0.0005	0.02	Uehara and Yang 2004
Sodium Hyaluronate*	0.0005	0.02	Hammond *et al.* 2006
Proteins/polypeptides	0.00001	0.01	Kelly and Roddick-Lanzilotta 2004

*Individual polymer, group 'Other'

5. Market penetration

5a. Laundry detergent

Supplemental Data 5a: Estimated market penetration (F_{prod}) of polymer groups for laundry detergent.
Note that polymers grouped as 'Other' are treated separately, as individual polymers. Groups/polymers are listed in order of highest market penetration for this product type.

Group/polymer	No. products containing group/polymer	Total no. products	F_{prod}
Alcohol alkoxylates	203	206	0.99
Alcohol ethoxylate salts	163	206	0.79
Polyethers and copolymers	146	206	0.71
Silicones	146	206	0.71
Polycarboxylates	130	206	0.63
Cellulose and derivatives	105	206	0.51
Polyethylenimine ethoxylates and polyether copolymers	83	206	0.40
Starch and derivatives	58	206	0.28
Polyvinyl alcohol	44	206	0.21
Polyquaterniums	33	206	0.16
Plant gums	22	206	0.11
Poly(oxy)alkylene terephthalates	19	206	0.092
Ethoxylated m-toluidine*	19	206	0.092
Polyesters	15	206	0.073
Polymeric colourants	14	206	0.068
Amine/formaldehyde polymers	12	206	0.058
Vinylimidazole/vinylpyrrolidone homo- and co-polymers	11	206	0.053
Fatty acid ethoxylates	7	206	0.034
Polymerised aromatic sulfonate salts	7	206	0.034
Polyvinylpyridine-N-oxide*	3	206	0.015
Proteins/polypeptides	1	206	0.0049
Hemicelluose*	1	206	0.0049
Lignin*	1	206	0.0049

*Individual polymer, group 'Other'

5b. Machine dishwashing detergent

Supplemental data 5b: Estimated market penetration (F_{prod}) of polymer groups for machine dishwashing detergent. Note that polymers grouped as 'Other' are treated separately, as individual polymers. Groups/polymers are listed in order of highest market penetration for this product type.

Group/polymer	No. products containing group/polymer	Total no. products	F_{prod}
Alcohol alkoxylates	56	59	0.95
Polycarboxylates	56	59	0.95
Polyethers and copolymers	49	59	0.83
Cellulose and derivatives	43	59	0.73
Polyvinyl alcohol	41	59	0.69
Starch and derivatives	30	59	0.51
Silicones	26	59	0.44
Polyquaterniums	11	59	0.19
Plant gums	2	59	0.034
Alginic acid*	2	59	0.034

*Individual polymer, group 'Other'

5c. Hand dishwashing detergent

Supplemental Data 5c: Estimated market penetration (F_{prod}) of polymer groups for hand dishwashing detergent. Groups are listed in order of highest market penetration for this product type.

Group	No. products containing group/polymer	Total no. products	F_{prod}
Alcohol ethoxylate salts	25	39	0.64
Polyethers and copolymers	22	39	0.56
Polyethylenimine ethoxylates and polyether copolymers	15	39	0.38
Alcohol alkoxylates	8	39	0.21
Polyquaterniums	2	39	0.051

5d. Toilet cleaner and bleach

Supplemental Data 5d: Estimated market penetration (F_{prod}) of polymer groups for toilet cleaner and bleach. Groups are listed in order of highest market penetration for this product type.

Group/polymer	No. products containing group/polymer	Total no. products	F_{prod}
Alcohol alkoxylates	18	38	0.47
Silicones	7	38	0.18
Cellulose and derivatives	6	38	0.16
Alcohol ethoxylate salts	6	38	0.16
Plant gums	4	38	0.11
Polymeric colourants	2	38	0.053

5e. Bodywash

Supplemental Data 5e: Estimated market penetration (F_{prod}) of polymer groups for bodywash. Note that polymers grouped as 'Other' are treated separately, as individual polymers. Groups/polymers are listed in order of highest market penetration for this product type.

Group/polymer	No. products containing group/polymer	Total no. products	F_{prod}
Alcohol ethoxylate salts	239	302	0.79
Polyquaterniums	137	302	0.45
Polycarboxylates	135	302	0.45
Polyol ethoxylate esters	134	302	0.44
Alcohol alkoxylates	73	302	0.24
Fatty acid ethoxylates	57	302	0.19
Polyethers and copolymers	37	302	0.12
Starch and derivatives	14	302	0.046
Plant gums	12	302	0.040
Polyglyceryl esters and polyglycerin	10	302	0.033
Hydrolysed proteins and derivatives	8	302	0.026
Proteins/polypeptides	6	302	0.020
Disodium Laureth Sulfosuccinate*	5	302	0.017
Polyurethane Crosspolymer-2*	5	302	0.017
Cellulose and derivatives	4	302	0.013
Polyolefins	4	302	0.013
Polymerised aromatic sulfonate salts	1	302	0.0033
Butyl Acrylate/Ethyltrimonium Chloride Methacrylate/Styrene Copolymer*	1	302	0.0033
PEG-4 Rapeseedamide*	1	302	0.0033

*Individual polymer, group 'Other'

5f. Handwash

Supplemental Data 5f: Estimated market penetration (F_{prod}) of polymer groups for handwash. Note that polymers grouped as 'Other' are treated separately, as individual polymers. Groups/polymers are listed in order of highest market penetration for this product type.

Group/polymer	No. products containing group/polymer	Total no. products	F_{prod}
Alcohol ethoxylate salts	71	97	0.73
Polyquaterniums	33	97	0.34
Polycarboxylates	26	97	0.27
Alcohol alkoxylates	25	97	0.26
Polyol ethoxylate esters	20	97	0.21
Fatty acid ethoxylates	15	97	0.15
Polyethers and copolymers	3	97	0.031
Hydrolysed proteins and derivatives	2	97	0.021
Plant gums	2	97	0.021
Proteins/polypeptides	2	97	0.021
Starch and derivatives	2	97	0.021
PEG-4 Rapeseedamide*	2	97	0.021
Polyglyceryl esters and polyglycerin	1	97	0.010

*Individual polymer, group 'Other'

5g. Soap bars

Supplemental Data 5g: Estimated market penetration (F_{prod}) of polymer groups for soap bars. Note that polymers grouped as 'Other' are treated separately, as individual polymers. Groups/polymers are listed in order of highest market penetration for this product type.

Group/polymer	No. products containing group/polymer	Total no. products	F_{prod}
Alcohol ethoxylate salts	2	56	0.036
Polyethers and copolymers	2	56	0.036
Alcohol alkoxylates	1	56	0.018
Cationic silicones	1	56	0.018
Silicones	1	56	0.018
Starch and derivatives	1	56	0.018
Hydroxypropyl Cyclodextrin*	1	56	0.018

*Individual polymer, group 'Other'

5h. Bath liquid

Supplemental Data 5h: Estimated market penetration (F_{prod}) of polymer groups for bath liquid. Note that polymers grouped as 'Other' are treated separately, as individual polymers. Groups/polymers are listed in order of highest market penetration for this product type.

Group/polymer	No. products containing group/polymer	Total no. products	F_{prod}
Alcohol ethoxylate salts	41	62	0.66
Polyquaterniums	25	62	0.40
Alcohol alkoxylates	21	62	0.34
Polyol ethoxylate esters	19	62	0.31
Fatty acid ethoxylates	12	62	0.19
Polycarboxylates	12	62	0.19
Polyethers and copolymers	3	62	0.048
Plant gums	1	62	0.016
Proteins/polypeptides	1	62	0.016
Disodium Laureth Sulfosuccinate*	1	62	0.016
PEG-4 Rapeseedamide*	1	62	0.016

*Individual polymer, group 'Other'

5i. Shampoo

Supplemental Data 5i: Estimated market penetration (F_{prod}) of polymer groups for shampoo. Note that polymers grouped as 'Other' are treated separately, as individual polymers. Groups/polymers are listed in order of highest market penetration for this product type.

Group/polymer	No. products containing group/polymer	Total no. products	F_{prod}
Polyquaterniums	243	266	0.91
Alcohol ethoxylate salts	173	266	0.65
Alcohol alkoxylates	92	266	0.35
Silicones	80	266	0.30
Polyethers and copolymers	70	266	0.26
Polyol ethoxylate esters	57	266	0.21
Fatty acid ethoxylates	52	266	0.20
Polycarboxylates	46	266	0.17
Hydrolysed protein and derivatives	40	266	0.15
Cationic silicones	29	266	0.11
Cellulose and derivatives	23	266	0.086
Starch and derivatives	21	266	0.079
Methyl Gluceth-10*	14	266	0.053
Plant gums	12	266	0.045
Silicone alkoxylates	11	266	0.041
Disodium Laureth Sulfosuccinate*	10	266	0.038
Polyesters	4	266	0.015
Sodium Hyaluronate*	3	266	0.011
Polyglyceryl esters and polyglycerin	2	266	0.0075
Proteins/polypeptides	2	266	0.0075
Poly(Linseed Oil)*	2	266	0.0075
Polymerised aromatic sulfonate salts	1	266	0.0038
Vinylimidazole/vinylpyrrolidone homo- and co-polymers	1	266	0.0038
Laureth-5 Carboxylic Acid*	1	266	0.0038

*Individual polymer, group 'Other'

5j. Conditioner

Supplemental Data 5j: Estimated market penetration (F_{prod}) of polymer groups for conditioner. Note that polymers grouped as ‘Other’ are treated separately, as individual polymers. Groups/polymers are listed in order of highest market penetration for this product type.

Group/polymer	No. products containing group/polymer	Total no. products	F_{prod}
Cationic silicones	117	228	0.51
Silicones	95	228	0.42
Alcohol alkoxylates	86	228	0.38
Polyquaterniums	57	228	0.25
Hydrolysed protein and derivatives	39	228	0.17
Polyol ethoxylate esters	24	228	0.11
Starch and derivatives	24	228	0.11
Fatty acid ethoxylates	20	228	0.088
Cellulose and derivatives	15	228	0.066
Plant gums	15	228	0.066
Polyethers and copolymers	15	228	0.066
Polyesters	10	228	0.044
Polycarboxylates	9	228	0.039
Silicone alkoxylates	8	228	0.035
PPG-3 Benzyl Ether Myristate*	5	228	0.022
Polymerised aromatic sulfonate salts	3	228	0.013
Alcohol ethoxylate salts	2	228	0.0088
Polyglyceryl esters and polyglycerin	2	228	0.0088
Vinylimidazole/vinylpyrrolidone homo- and co-polymers	2	228	0.0088
Sodium Hyaluronate*	2	228	0.0088
Proteins/polypeptides	1	228	0.0044
Poly(Linseed Oil)*	1	228	0.0044

*Individual polymer, group ‘Other’

6. Product usage

Supplemental Data 6: Values for product usage (U_{prod}) used for each product type, collated from literature data.

**Product type**	**U_{prod} (g capita^{-1} day^{-1})**	**References**	**Notes**
Laundry detergent	11.3	Eriksson *et al.* 2002; A.I.S.E. 2019	Value range of 10.1-20.5 g pc^{-1} day^{-1} for USA, Sweden, Denmark, Finland and Norway in 2002 (Eriksson *et al.* 2002). Laundry detergent tonnage in Europe decreased by 45% since 1997 in 2017 (A.I.S.E. 2019). Assuming a similar decrease from 2002-2021 and taking upper limit (20.5 g pc^{-1} day^{-1}) gives 65% of 20.5 = 11.3 g capita^{-1} day^{-1} calculated in the present study.
Machine dishwashing detergent	2.4	DeLeo *et al.* 2020	California sales of machine dishwashing detergent in 2015 = 206,180 lb day^{-1}, and population of California (in 2018) = 39,557,045 people (DeLeo *et al.* 2020). Usage therefore calculated in present study as 2.4 g capita^{-1} day^{-1} (1dp).
Hand dishwashing detergent	5.0	Schneider *et al.* 2019	Arithmetic mean of use frequency per individual for frequent users = 0.9 day^{-1}, and amount used per event = 5.5 g (Schneider *et al.* 2019). Therefore usage calculated in present study = 5.0 g capita^{-1} day^{-1}.
Toilet cleaners and bleach	4.3	HERA 2005	Values given for toilet cleaner liquid = 30 ml per task and typically 1 task per week (HERA 2005). Assuming 1 ml = 1 g of toilet cleaner and values correspond to per person, usage calculated in the present study as 4.3 g^{-1} capita^{-1} day^{-1}. Note limited data were available, and the obtained value may be higher than actual usage, based on values for other products from the same source.
Bodywash	8.3	Garcia-Hidalgo *et al.* 2017	Mean amount used per day by adults (Garcia-Hidalgo *et al.* 2017).
Handwash	10.3	Garcia-Hidalgo *et al.* 2017	Mean amount used per day by adults (Garcia-Hidalgo *et al.* 2017).
Soap bars	3.2	Gomez-Berrada *et al.* 2017	Mean amount used per day by adults (Gomez-Berrada *et al.* 2017).
Bath liquid	0.8	Garcia-Hidalgo *et al.* 2017	Mean amount used per day by adults (Garcia-Hidalgo *et al.* 2017).
Shampoo	2.9	Garcia-Hidalgo *et al.* 2017	Mean amount used per day by adults (Garcia-Hidalgo *et al.* 2017).
Conditioner	2.9	Garcia-Hidalgo *et al.* 2017	Mean amount used per day by adults (Garcia-Hidalgo *et al.* 2017).

7. Down-the-drain emissions of polymer groups

Supplemental Data 7: Down-the-drain emissions (M_{DTT}; g capita^{-1} day^{-1}) for polymer groups in household products. Polymer groups are listed in order of highest maximum M_{DTT}.

**Polymer groups**	**Min. M_{DTT}**	**Max. M_{DTT}**
Alcohol ethoxylate salts	1.1	2.4
Alcohol alkoxylates	0.8	1.8
Polycarboxylates	0.1	1.0
Polyol ethoxylate esters	0.07	0.3
Polyethers and copolymers	0.04	0.3
Starch and derivatives	0.06	0.2
Silicones	0.05	0.2
Polyquaterniums	0.007	0.2
Polyvinyl alcohol	0.02	0.1
Cellulose and derivatives	0.03	0.1
Fatty acid ethoxylates	0.02	0.1
Polyethylenimine ethoxylates and polyether copolymers	0.009	0.08
Other	0.01	0.06
Polyesters	0.008	0.05
Proteins/polypeptides	0.001	0.04
Hydrolysed protein and derivatives	0.0004	0.03
Cationic silicones	0.005	0.03
Poly(oxy)alkylene terephthalates	0.005	0.03
Plant gums	0.002	0.03
Polyolefins	0.003	0.01
Polyglyceryl esters and polyglycerin	0.0007	0.01
Polymerised aromatic sulfonate salts	0.003	0.01
Vinylimidazole/vinylpyrrolidone homo- and co-polymers	0.002	0.007
Silicone alkoxylates	0.0004	0.003
Polymeric colourants	0.00006	0.0007
Amine/formaldehyde polymers	0.00002	0.0002

8. Polymer grouping and individual emissions

Supplemental Data 8: Polymers identified in UK household products released down-the-drain at point-of-use, along with their assigned groupings based on monomer type, polymer structure, functional groups, and use in products.

Polymer groups are listed in order of highest maximum down-the-drain emissions (M_{DTT}) for the entire group (see Supplemental Data 7). Individual polymers in each group are also listed in order of highest M_{DTT}. Example polymer structures are shown for the top polymer (i.e. highest emissions) in each of the top ten groups, with structural features ubiquitous to all group members (i.e. key chemical functionalities) highlighted in red. Note that in some cases, other members of the polymer groups contain other additional chemical functionalities, which may differ in structure to the examples shown.

Polymer group	Individual polymers	Min. M_{DTT} (g capita^{-1} day^{-1})	Max. M_{DTT} (g capita^{-1} day^{-1})
Alcohol ethoxylate salts	Sodium Laureth Sulfate	1.60E+00	3.38E+00
e.g. sodium laureth sulfate	MEA-Laureth Sulfate	3.57E-01	7.57E-01
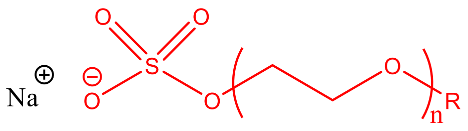	Sodium C12-15 Pareth Sulfate	1.11E-01	2.35E-01
R = fatty hydrocarbon chain, C12 for sodium laureth sulfate	Sodium C12-14 Pareth-3 Sulfate	5.21E-02	1.11E-01
	Ammonium Laureth Sulfate	5.14E-02	1.09E-01
	Sodium Coceth-30 Sulfate	3.38E-02	7.16E-02
	Sodium C12-13 Pareth Sulfate (A) / Sodium Laureth Sulfate (B)	3.27E-02	6.94E-02
	Sodium C12-15 Pareth-3 Sulfate	1.45E-02	3.07E-02
	Zinc Coceth Sulfate	4.94E-03	1.05E-02
	Alcohols, C12-14, ethoxylated, sulfates, sodium salts	4.83E-03	1.02E-02
	Sodium Myreth Sulfate	3.67E-03	7.78E-03
	Magnesium Laureth Sulfate	2.38E-03	5.04E-03
	Magnesium Laureth-8 Sulfate	2.38E-03	5.04E-03
	Magnesium Oleth Sulfate	2.38E-03	5.04E-03
	Sodium Laureth-8 Sulfate	2.38E-03	5.04E-03
	Sodium Oleth Sulfate	2.38E-03	5.04E-03
	MIPA Laureth Sulfate	1.29E-03	2.74E-03
	Sodium Trideceth Sulfate	1.29E-03	2.74E-03
	MIPA C12-15 Pareth Sulfate	1.19E-03	2.52E-03
Alcohol alkoxylates	Laureth-4	2.14E-01	5.10E-01
e.g. laureth-4	PEG/PPG-10/2 Propylheptyl Ether	1.38E-01	3.29E-01
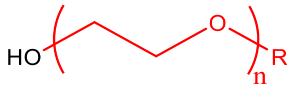	C11-15 Pareth-7	1.08E-01	2.57E-01
R = fatty hydrocarbon chain, C12 for laureth-4	C11-15 Pareth-40	1.06E-01	2.53E-01
	C12-14 Pareth-7	1.00E-01	2.38E-01
	C12-14 Pareth-n	9.24E-02	2.20E-01
	Trideceth-n	6.48E-02	1.54E-01
	C14-15 Pareth-7	5.85E-02	1.39E-01
	C14-15 Pareth-n	5.85E-02	1.39E-01
	C12-15 Pareth-7	5.39E-02	1.28E-01
	Trideceth-9	4.95E-02	1.18E-01
	PEG 6 - Methyl Ether	3.85E-02	9.16E-02
	C9-11 Pareth-n	3.83E-02	9.12E-02

Steareth-20	2.90E-02	6.91E-02
2-Propylheptanol ethoxylated	2.87E-02	6.84E-02
Trideceth-7	2.39E-02	5.70E-02
Fatty alcohol alkoxylate	2.39E-02	5.69E-02
Laureth-7	2.30E-02	5.47E-02
C15 Pareth-n	2.15E-02	5.13E-02
Laureth-3	2.00E-02	4.76E-02
C12-16 Pareth-n	1.69E-02	4.03E-02
Ceteareth-25	1.42E-02	3.38E-02
PPG-5-Ceteth-20	1.34E-02	3.19E-02
Alcohols, C12-16, ethoxylated, 7-16 EO	1.25E-02	2.97E-02
C9-11 Pareth-6	1.25E-02	2.97E-02
Laureth-10	1.11E-02	2.65E-02
Steareth-4	1.03E-02	2.44E-02
Primary alcohol ethoxylate	9.72E-03	2.31E-02
Deceth-8	9.58E-03	2.28E-02
PEG-8 Propylheptyl Ether	9.58E-03	2.28E-02
Ceteareth-80	7.70E-03	1.83E-02
C9-11 Pareth-8	7.49E-03	1.78E-02
PEG-7 Propylheptyl Ether	6.85E-03	1.63E-02
Trideceth-10	6.74E-03	1.60E-02
PPG-1 Trideceth-6	5.08E-03	1.21E-02
Alkylethoxylate C9-11, 5.5EO	4.99E-03	1.19E-02
Ethoxylated Alcohol	4.99E-03	1.19E-02
Laureth-23	4.70E-03	1.12E-02
Trideceth-6	4.30E-03	1.02E-02
Trideceth-12	3.94E-03	9.37E-03
C12-13 Pareth-n	3.74E-03	8.90E-03
Alcohols C12-14, ethoxylated (7EO)	3.08E-03	7.33E-03
C12-16 pareth-7	3.08E-03	7.33E-03
Pareth-7	3.08E-03	7.33E-03
Alkylethoxylate, C10-16, 10EO	2.50E-03	5.94E-03
C9-11 Pareth-9	2.50E-03	5.94E-03
Undeceth-40	2.50E-03	5.94E-03
PEG-4 Distearyl Ether	2.29E-03	5.45E-03
Steareth-21	2.24E-03	5.34E-03
Ceteareth-20	2.21E-03	5.26E-03
C12-13 Pareth-3	1.73E-03	4.12E-03
C13-15 Pareth-7	1.54E-03	3.66E-03
Ceteareth-15	1.54E-03	3.66E-03
Alcohol alkoxylate	1.50E-03	3.56E-03
Modified Fatty alcohol polyglycolether	1.50E-03	3.56E-03
Polyoxyethylene trimethyldecyl alcohol	1.50E-03	3.56E-03
PPG-5-Laureth-5	1.50E-03	3.56E-03
C12-13 Pareth-6	7.48E-04	1.78E-03
Laureth-5	5.98E-04	1.42E-03
C12-13 Pareth-23	5.91E-04	1.41E-03
PPG-3 myristyl ether	5.91E-04	1.41E-03

	Laureth-16	4.48E-04	1.07E-03
	Laureth-9	3.70E-04	8.82E-04
	Coceth-7	1.49E-04	3.56E-04
	Macrogol Lauryl Ether (4)	1.49E-04	3.56E-04
	Oleth-20	1.49E-04	3.56E-04
	PPG-1-PEG-9 Lauryl Glycol Ether	1.49E-04	3.56E-04
Polycarboxylates	Styrene/Acrylates Copolymer	7.76E-02	7.68E-01
e.g. styrene/acrylates copolymer	Sodium Polyacrylate	3.54E-02	3.51E-01
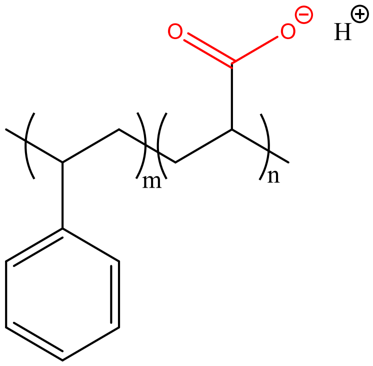	Sodium Acrylic Acid/MA Copolymer	3.24E-02	3.21E-01
	Acrylates Copolymer	2.19E-02	2.17E-01
	2- propenoic acid, homopolymer, sodium salt, sulfonated	6.04E-03	5.97E-02
	Acrylic acid sodium salt polymer, sodium sulfonate terminated	4.07E-03	4.03E-02
	Copolymer of acrylic and sulphonic acids	3.58E-03	3.54E-02
	Modified Polycarboxylate	2.88E-03	2.85E-02
	Maleic- acrylic acid copolymer sodiumsalt	2.68E-03	2.65E-02
	Carbomer	2.34E-03	2.32E-02
	Acrylic acid maleic acid polymer	1.79E-03	1.77E-02
	Polycarboxylate, sodium salt	1.34E-03	1.33E-02
	Acrylates/Steareth-20 Methacrylate Copolymer	1.12E-03	1.11E-02
	Acrylates/C10-30 Alkyl Acrylate Crosspolymer	1.07E-03	1.06E-02
	Sodium polyaspartate	8.94E-04	8.85E-03
	2-Propenoic acid, homopolymer, sodium salt	8.94E-04	8.85E-03
	Acrylates/Steareth-20 Methacrylate Crosspolymer	8.69E-04	8.60E-03
	Sodium Acrylates Copolymer	8.69E-04	8.60E-03
	Ethylene/MA Copolymer	7.21E-04	7.13E-03
	Methacrylic acid and acrylic acid ester copolymer	7.21E-04	7.13E-03
	No EU INCI name - Acrylic Copolymer	7.21E-04	7.13E-03
	Acrylates/Beheneth-25 Methacrylate Copolymer	5.10E-04	5.05E-03
	Acrylates/PEG-10 Maleate/Styrene Copolymer	4.81E-04	4.76E-03
	Sodium polyitaconate	4.47E-04	4.42E-03
	Acrylates Crosspolymer-4	3.38E-04	3.34E-03
	Polyacrylate-33	2.86E-04	2.83E-03
	2-Propenoic Acid, Telomer with Sodium Hydrogen Sulfite, Sodium Salt	2.24E-04	2.21E-03
	Polyacrylic Acid	2.24E-04	2.21E-03
	Acrylates/Palmeth-25 Acrylate Copolymer	1.50E-04	1.49E-03
	Sodium Styrene/Acrylates Copolymer	1.24E-04	1.23E-03
	Acrylates/Ammonium Methacrylate Copolymer	1.24E-04	1.23E-03
	PVM/MA Copolymer	8.90E-05	8.80E-04
	Acrylic Acid/Acrylamidomethyl Propane Sulfonic Acid Copolymer	7.51E-05	7.43E-04
	Polyacrylate-1 Crosspolymer	7.51E-05	7.43E-04

Polyol ethoxylate esters

e.g. PEG-7 glyceryl cocoate

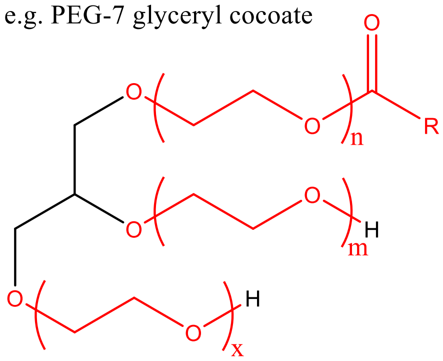

R = fatty hydrocarbon chain, mixture of chain lengths for PEG-7 glyceryl cocoate

PEG-7 Glyceryl Cocoate	3.92E-02	1.98E-01
PEG-200 Hydrogenated Glyceryl Palmate	2.94E-02	1.48E-01
Polysorbate 20	2.30E-02	1.16E-01
PEG-120 Methyl Glucose Dioleate	1.49E-02	7.51E-02
PEG-6 Caprylic/Capric Glycerides	6.88E-03	3.47E-02
PEG-150 Pentaerythrityl Tetrastearate	6.63E-03	3.34E-02
Shea Butter Glycereth-8 Esters	5.81E-03	2.93E-02
PEG-80 Sorbitan Laurate	3.87E-03	1.95E-02
Polysorbate 60	1.33E-03	6.70E-03
PEG-9 Cocoglycerides	7.96E-04	4.01E-03
PEG-90 Glyceryl Isostearate	6.22E-04	3.13E-03
PEG-60 Almond Glycerides	3.63E-04	1.83E-03
PEG-10 Olive Glycerides	3.11E-04	1.57E-03
PEG-200 Hydrogenated Glyceryl Cocoate	3.11E-04	1.57E-03
PEG-120 Methyl Glucose Trioleate	1.81E-04	9.14E-04

Polyethers and copolymers

e.g. PPG-26

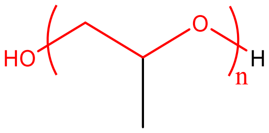

PPG-26	1.92E-02	1.38E-01
Co-polymer of PEG / Vinyl Acetate	9.54E-03	6.88E-02
Polyethylene Glycol	8.32E-03	6.00E-02
PPG-12	6.33E-03	4.56E-02
PPG-9	6.00E-03	4.32E-02
PPG-34	4.85E-03	3.50E-02
PPG-6	3.96E-03	2.85E-02
Polyethylene Glycol MW >4100	3.32E-03	2.39E-02
Polyethylene Glycol MW <4100	2.97E-03	2.14E-02
PEG Copolymer	2.21E-03	1.59E-02
PEG-45M	2.18E-03	1.57E-02
PEG-75	1.63E-03	1.17E-02
PEG-33	1.57E-03	1.13E-02
PEG-4	1.27E-03	9.13E-03
PEG-80	1.16E-03	8.39E-03
Ethylene/propylene oxide copolymer	1.05E-03	7.55E-03
PEG-130 - PEG-150	1.05E-03	7.55E-03
PEG-135	6.99E-04	5.04E-03
Poloxamer 407	6.67E-04	4.81E-03
PEG-14M	6.16E-04	4.44E-03
Poloxamer 124	5.82E-04	4.20E-03
Peg-30 - Peg-40	5.24E-04	3.78E-03
PEG-23M	4.46E-04	3.22E-03
PEG-10	3.49E-04	2.52E-03
PEG-90	3.49E-04	2.52E-03
Poloxalene	3.49E-04	2.52E-03
PEG-2M	2.94E-04	2.12E-03
PEG-180M	2.22E-04	1.60E-03
PEG-180	2.21E-04	1.59E-03
PEG- 30 - PEG-150	1.75E-04	1.26E-03
PEG-150	1.75E-04	1.26E-03
PEG-32	1.75E-04	1.26E-03

	PEG-8	1.75E-04	1.26E-03
	PEG-9	1.49E-04	1.07E-03
	PPG-n	1.16E-04	8.39E-04
	PEG-90M	1.01E-04	7.26E-04
Starch and derivatives<br>e.g. dextrin<br>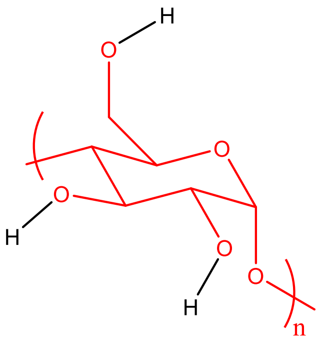	Dextrin	6.49E-02	2.54E-01
	Oryza sativa (rice) starch	2.35E-02	9.19E-02
	Hydrogenated Starch Hydrolysate	1.02E-02	3.97E-02
	Corn Starch Modified	9.89E-03	3.87E-02
	Sodium Starch Octenylsuccinate	4.69E-03	1.83E-02
	Maltodextrin	3.18E-03	1.24E-02
	Tapioca Starch	2.15E-03	8.42E-03
	Sodium Hydroxypropyl Starch Phosphate	1.41E-03	5.50E-03
	Sodium Hydrolyzed Potato Starch Dodecenylsuccinate	1.35E-03	5.29E-03
	Carboxymethylinulin	1.24E-03	4.83E-03
	Hydroxypropyl starch phosphate	9.01E-04	3.52E-03
	Triticum vulgare (wheat) starch	7.31E-04	2.86E-03
	Starch	6.18E-04	2.42E-03
	Potato Starch Modified	3.07E-04	1.20E-03
	Zea mays (corn) starch	3.07E-04	1.20E-03
	Saccharide Isomerate	2.81E-04	1.10E-03
	Potato Starch	1.27E-04	4.98E-04
Silicones<br>e.g. dimethicone<br>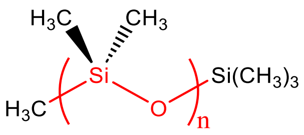	Dimethicone	4.75E-02	2.00E-01
	Dimethiconol	1.38E-02	5.83E-02
	Trimethylsiloxysilicate	7.21E-03	3.03E-02
	Phenylpropyl Ethyl Methicone	6.47E-03	2.72E-02
	Simethicone	6.28E-03	2.64E-02
	Silicone Compound	5.54E-03	2.33E-02
	Siloxanes and Silicones, di-Me, Me octyl, Me 2-phenylpropyl	4.80E-03	2.02E-02
	Phenylpropyl Dimethicone	2.40E-03	1.01E-02
	Methicone	6.11E-04	2.57E-03
	Phenyl Trimethicone	4.59E-04	1.93E-03
	C30-45 Alkyl Dimethicone	3.45E-04	1.45E-03
	Divinyldimethicone/dimethicone copolymer	3.27E-04	1.37E-03
	Dimethiconol/Silsesquioxane Copolymer	2.91E-04	1.22E-03
	Trimethylsiloxysilicate/Dimethicone Crosspolymer	1.85E-04	7.78E-04
	Caprylyl Methicone	1.15E-04	4.83E-04
Polyquaterniums	Polyquaternium-7	8.63E-03	1.83E-01
	Guar Hydroxypropyltrimonium Chloride	2.54E-03	5.41E-02
	Polyquaternium-10	1.16E-03	2.48E-02
	Polyethylenimine	6.12E-04	1.30E-02
	Hydroxypropyl Guar Hydroxypropyltrimonium Chloride	2.99E-04	6.37E-03
	Polyquaternium-37	2.92E-04	6.21E-03
	Polyquaternium-2	1.91E-04	4.06E-03
	Acrylamidopropyltrimonium Chloride/Acrylamide Copolymer	1.88E-04	4.01E-03

e.g. polyquaternium-7<br>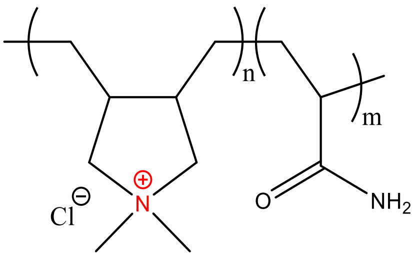	Polyquaternium-6	1.30E-04	2.76E-03
	Aziridine, homopolymer	7.65E-05	1.63E-03
	Polyquaternium-39	6.03E-05	1.28E-03
	Polyquaternium-47	5.75E-05	1.22E-03
	Polyquaternium-68	5.66E-05	1.20E-03
	Polyquaternium-70	4.04E-05	8.60E-04
	Polyquaternium-22	3.87E-05	8.23E-04
	Starch Hydroxypropyltrimonium Chloride	3.77E-05	8.01E-04
	PEI-2500	2.58E-05	5.49E-04
	Polyquaternium-55	2.25E-05	4.78E-04
	PG-Hydroxyethylcellulose cocodimonium chloride	1.71E-05	3.63E-04
	Polyquaternium-28	1.71E-05	3.63E-04
	Polyquaternium-52	1.62E-05	3.45E-04
	Polyquaternium-16	1.17E-05	2.48E-04
	Polyquaternium-4	1.17E-05	2.48E-04
	Polyquaternium-76	1.08E-05	2.30E-04
	Modified Guar Hydroxypropyltrimonium Chloride	5.40E-06	1.15E-04
	Polyacrylamidopropyltrimonium chloride	5.40E-06	1.15E-04
	Polyquaternium-30	5.40E-06	1.15E-04
Polyvinyl alcohol<br>polyvinyl alcohol<br>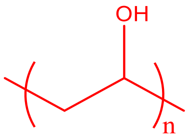	Polyvinyl Alcohol	3.92E-02	2.35E-01
	Polyvinyl alcohol film	7.94E-04	4.77E-03
	Thermal shrinkable PVOH film	7.94E-04	4.77E-03
Cellulose and derivatives<br>e.g. hydroxyethylcellulose<br>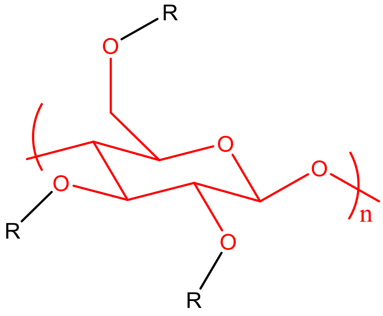<br>R = -H or -CH_2CH_2OH for hydroxyethylcellulose	Hydroxyethylcellulose	2.47E-02	9.41E-02
	Cellulose Gum	1.91E-02	7.28E-02
	Microcrystalline Cellulose	1.04E-02	3.98E-02
	Cellulose	5.72E-03	2.18E-02
	Hydroxypropyl Methylcellulose	2.52E-03	9.59E-03
	Cetyl Hydroxyethylcellulose	1.14E-04	4.36E-04
Fatty acid ethoxylates	PEG-40 Hydrogenated Castor Oil	2.47E-02	1.06E-01
	PEG-150 distearate	1.02E-02	4.33E-02
	PEG-3 Distearate	4.13E-03	1.76E-02
	PEG-20 Stearate	3.85E-03	1.65E-02
	PEG-35 Castor Oil	1.40E-03	5.98E-03
	PEG-4 Dilaurate	1.18E-03	5.02E-03
	PEG-4 Laurate	1.18E-03	5.02E-03
	PEG Distearate	6.42E-04	2.74E-03

	PEG-55 Propylene Glycol Oleate	4.27E-04	1.82E-03
	PEG-40 Castor Oil	2.35E-04	1.00E-03
	PEG-60 Hydrogenated Castor Oil	1.78E-04	7.59E-04
	PEG-20 Castor Oil	1.65E-04	7.05E-04
Polyethylenimine ethoxylates and polyether copolymers	PEI Ethoxylate	8.36E-03	7.90E-02
	Aziridine homopolymer ethoxylated	6.09E-03	5.76E-02
	PEI/PEG/PPG Copolymer	3.05E-03	2.88E-02
Other	Ethoxylated m-toluidine	4.64E-03	2.60E-02
	Disodium Laureth Sulfosuccinate	4.17E-03	2.34E-02
	Methyl Gluceth-10	3.12E-03	1.75E-02
	Polyurethane Crosspolymer-2	1.83E-03	1.02E-02
	PEG-4 Rapeseedamide	1.62E-03	9.07E-03
	Alginic acid	1.45E-03	8.14E-03
	Hydroxypropyl Cyclodextrin	1.02E-03	5.71E-03
	Sodium Hyaluronate	8.05E-04	4.51E-03
	Polyvinylpyridine-N-Oxide	7.32E-04	4.10E-03
	Poly (Linseed Oil)	5.14E-04	2.88E-03
	Butyl Acrylate/Ethyltrimonium Chloride Methacrylate/Styrene Copolymer	3.66E-04	2.05E-03
	PPG-3 Benzyl Ether Myristate	3.40E-04	1.91E-03
	Hemicellulose	2.44E-04	1.37E-03
	Lignin	2.44E-04	1.37E-03
	Laureth-5 Carboxylic Acid	2.23E-04	1.25E-03
Polyesters	Anionic modified polyester	1.51E-02	8.23E-02
	Hydrogenated Castor Oil/Sebacic Acid Copolymer	8.65E-04	4.72E-03
	Capryloyl Glycerin/Sebacic Acid Copolymer	6.99E-04	3.82E-03
Proteins/polypeptides	Whey Protein	1.92E-03	7.80E-02
	Gelatin	2.70E-05	1.10E-03
	Keratin	5.82E-06	2.36E-04
	Triticum Vulgare Gluten	2.68E-06	1.09E-04
Hydrolysed protein and derivatives	Hydrolyzed keratin	2.84E-04	1.89E-02
	Hydrolysed Milk Protein	7.11E-05	4.72E-03
	Silk Amino Acids	6.94E-05	4.60E-03
	Hydrolyzed Silk	6.65E-05	4.41E-03
	Hydrolyzed Rice Protein	6.05E-05	4.01E-03
	Hydrolyzed collagen	5.55E-05	3.68E-03
	Hydrolysed Wheat Protein	5.21E-05	3.46E-03
	Hydroxypropyltrimonium Hydrolyzed Wheat Protein	4.33E-05	2.87E-03
	Hydrolyzed vegetable protein PG-propyl silanetriol	4.16E-05	2.76E-03
	Hydrolyzed corn protein	3.21E-05	2.13E-03
	Hydrolyzed soy protein	3.21E-05	2.13E-03
	AMP-Isostearoyl Hydrolyzed Wheat Protein	2.78E-05	1.84E-03
	Cocoyl hydrolyzed keratin	1.39E-05	9.21E-04
	Ethyltrimonium Chloride Methacrylate/ Hydrolysed Wheat Protein Copolymer	1.39E-05	9.21E-04

	Hydrolyzed pea protein	1.39E-05	9.21E-04
	Hydrolyzed Wheat Gluten	1.24E-05	8.25E-04
	Laurdimonium Hydroxypropyl Hydrolyzed Keratin	7.79E-06	5.17E-04
Cationic silicones	Amodimethicone	4.11E-03	2.50E-02
	Bis-Aminopropyl Dimethicone	2.73E-03	1.66E-02
	PEG-7 Amodimethicone	1.01E-03	6.15E-03
	Bis-Cetearyl Amodimethicone	3.73E-04	2.27E-03
	Silicone Quaternium-26	3.11E-04	1.89E-03
	Bis-Hydroxy/Methoxy Amodimethicone	1.86E-04	1.14E-03
	Quaternium-80	1.86E-04	1.14E-03
	Silicone Quaternium-18	1.79E-04	1.09E-03
	Silicone quaternium-22	1.60E-04	9.75E-04
	Bis(C13-15 Alkoxy)PG-Amodimethicone	6.21E-05	3.78E-04
Polyoxyalkylene terephthalate/polyalkylene terephthalates	Polypropylene Terephthalate/ Polyoxyethylene terephthalate	8.78E-03	4.39E-02
	Polyethylene Terephthalate	1.65E-03	8.23E-03
Plant gums	Xanthan Gum	4.24E-03	4.34E-02
	Hydroxypropyl Guar	6.21E-04	6.36E-03
	Tamarindus Indica Seed Gum	1.07E-04	1.10E-03
Polyolefins	Synthetic Wax	3.30E-03	1.65E-02
	Hydrogenated Polydecene	1.10E-03	5.50E-03
	Polyethylene	1.10E-03	5.50E-03
Polyglyceryl esters and polyglycerin	Argan Oil Polyglyceryl-6 Esters	6.66E-04	1.21E-02
	Polyglyceryl-3 laurate	2.52E-04	4.58E-03
	Polyglyceryl-10 Stearate	1.68E-04	3.05E-03
	Polyglyceryl-3 caprate/caprylate/succinate	1.05E-04	1.91E-03
	Polyglycerin-10	6.29E-05	1.15E-03
	Polyglyceryl-10 Myristate	6.29E-05	1.15E-03
Polymerised aromatic sulfonate salts	Sodium Polynaphthalenesulfonate	5.16E-03	1.94E-02
	Calcium Divinylbenzene Styrene Copolymer Sulfonate	5.84E-04	2.19E-03
Vinylimidazole/vinylpyrrolidone homo- and co-polymers	PVP	2.01E-03	8.80E-03
	Copolymer of 1-vinylimidazole and 1-vinyl-2-pyrrolidone	5.02E-04	2.19E-03
	Polyvinylpirrolydone/Vinylimidazole copolymer	5.02E-04	2.19E-03
	VP/Methacrylamide/Vinyl Imidazole Copolymer	1.59E-04	6.97E-04
Silicone alkoxylates	PEG-12 Dimethicone	3.58E-04	2.78E-03
	Lauryl PEG/PPG-18/18 Methicone	1.47E-04	1.14E-03
	Dimethicone PEG-8 Meadowfoamate	4.91E-05	3.82E-04
	PEG/PPG-14/4 Dimethicone	4.91E-05	3.82E-04
	PEG/PPG-17/18 Dimethicone	4.91E-05	3.82E-04
	PEG/PPG-18/18 Dimethicone	4.91E-05	3.82E-04
Polymeric colourants	Polymeric Blue Colourant	2.41E-05	2.88E-04
	Polymeric Pink Colourant	2.41E-05	2.88E-04
	Polymeric Red Colourant	2.41E-05	2.88E-04

	Polymeric Yellow Colourant	2.41E-05	2.88E-04
	Liquitint® Orange 157	9.48E-06	1.13E-04
	Liquitint® Violet	9.48E-06	1.13E-04
Amine/formaldehyde polymers	Methoxypolyoxymethylene Melamine	3.04E-05	2.43E-04
	Polyoxymethylene Melamine	1.14E-05	9.11E-05
	Formamide, N-ethenyl-, homopolymer, hydrolyzed, sulfate	3.80E-06	3.04E-05
	Polyoxymethylene Melamine Urea	3.80E-06	3.04E-05

9. Predicted environmental concentration

9a. Polymer groups in surface water

Supplemental Data 9a: Predicted environmental concentration in surface water (PEC_{SW}) estimates (µg L^{-1}) for prioritised polymer groups (by down-the-drain emissions) in household products.

Note that literature data for WWT fate could not be obtained for polyol ethoxylate esters, and thus estimates assume no WWT removal to give a conservative worst-case scenario. Polymer groups are listed in order of highest max. PEC_{SW}. Values > 1 are listed to 0dp, values <1 are listed to 1sf.

Polymer groups	Min. PEC_{SW}	Max. PEC_{SW}
Polycarboxylates	2	915
Alcohol ethoxylate salts	1	731
Alcohol alkoxylates	0.8	370
Polyol ethoxylate esters	67	337
Polyquaterniums	5	142
Starch and derivatives	31	123
Polyethers and copolymers	2	90
Cellulose and derivatives	16	60
Polyvinyl alcohol	3	19
Silicones	1	12

9b. Polymer groups in soil

Supplemental Data 9b: Predicted environmental concentration in soil (PEC_{SOIL}) estimates (mg kg^{-1}) for prioritised polymer groups (by down-the-drain emissions) in household products.

Note that literature data for WWT fate could not be obtained for polyol ethoxylate esters, and thus estimates assume all polymers in this group are released in sludge to give a conservative worst-case scenario. Polymer groups are listed in order of highest max. PEC_{SOIL}. Values > 1 are listed to 0dp, values <1 are listed to 1sf.

Polymer groups	Min. PEC_{SOIL}	Max. PEC_{SOIL}
Polycarboxylates	0.4	39
Polyol ethoxylate esters	3	13
Silicones	2	8
Starch and derivatives	1	5
Polyethers and copolymers	0.7	5
Polyvinyl alcohol	0.5	3
Alcohol ethoxylate salts	1	3
Cellulose and derivatives	0.6	2
Polyquaterniums	0.02	2
Alcohol alkoxylates	0.2	0.7

9c. Individual polymers in surface water and soil

Supplemental Data 9c: Values of PEC_{SW} and PEC_{SOIL} for individual polymers from the top ten groups with the highest down-the-drain emissions (M_{DTT}). Polymers are listed in order of highest maximum M_{DTT} (reported in Supplemental Data 8).

	PEC_{SW} (µg L^{-1})		PEC_{SOIL} (mg kg^{-1})	
**Polymer**	**Min.**	**Max.**	**Min.**	**Max.**
Sodium Laureth Sulfate	7.98E-01	5.12E+02	6.66E-01	2.02E+00
Styrene/Acrylates Copolymer	7.76E-01	3.49E+02	1.39E-01	1.49E+01
MEA-Laureth Sulfate	1.79E-01	1.15E+02	1.49E-01	4.51E-01
Laureth-4	1.07E-01	5.25E+01	3.41E-02	1.01E-01
Sodium Polyacrylate	3.54E-01	1.60E+02	6.34E-02	6.83E+00
PEG/PPG-10/2 Propylheptyl Ether	6.91E-02	3.39E+01	2.20E-02	6.53E-02
Sodium Acrylic Acid/MA Copolymer	3.24E-01	1.46E+02	5.80E-02	6.25E+00
C11-15 Pareth-7	5.39E-02	2.64E+01	1.71E-02	5.10E-02
Dextrin	1.62E+01	6.34E+01	6.45E-01	2.52E+00
C11-15 Pareth-40	5.31E-02	2.60E+01	1.69E-02	5.02E-02
C12-14 Pareth-7	5.00E-02	2.45E+01	1.59E-02	4.73E-02
Polyvinyl Alcohol	3.09E+00	1.85E+01	4.77E-01	2.86E+00
Sodium C12-15 Pareth Sulfate	5.55E-02	3.57E+01	4.63E-02	1.40E-01
C12-14 Pareth-n	4.62E-02	2.26E+01	1.47E-02	4.37E-02
Acrylates Copolymer	2.19E-01	9.88E+01	3.93E-02	4.23E+00
Dimethicone	7.12E-01	5.99E+00	8.87E-01	3.85E+00
PEG-7 Glyceryl Cocoate	1.96E+01	9.88E+01	7.79E-01	3.93E+00
Polyquaternium-7	2.68E+00	8.43E+01	1.39E-02	1.39E+00
Trideceth-n	3.24E-02	1.59E+01	1.03E-02	3.07E-02
PEG-200 Hydrogenated Glyceryl Palmate	1.47E+01	7.40E+01	5.84E-01	2.94E+00
C14-15 Pareth-7	2.92E-02	1.43E+01	9.30E-03	2.77E-02
C14-15 Pareth-n	2.92E-02	1.43E+01	9.30E-03	2.77E-02
PPG-26	3.84E-01	2.08E+01	1.57E-01	1.13E+00
C12-15 Pareth-7	2.69E-02	1.32E+01	8.57E-03	2.55E-02
Trideceth-9	2.48E-02	1.21E+01	7.87E-03	2.34E-02
Polysorbate 20	1.15E+01	5.80E+01	4.57E-01	2.31E+00
Sodium C12-14 Pareth-3 Sulfate	2.61E-02	1.67E+01	2.18E-02	6.59E-02
Ammonium Laureth Sulfate	2.57E-02	1.65E+01	2.14E-02	6.49E-02
Hydroxyethylcellulose	6.18E+00	2.35E+01	2.46E-01	9.35E-01
Oryza sativa (rice) starch	5.88E+00	2.30E+01	2.34E-01	9.13E-01
PEG 6 - Methyl Ether	1.92E-02	9.43E+00	6.12E-03	1.82E-02
C9-11 Pareth-n	1.92E-02	9.39E+00	6.09E-03	1.81E-02
PEG-120 Methyl Glucose Dioleate	7.45E+00	3.76E+01	2.96E-01	1.49E+00
Cellulose Gum	4.78E+00	1.82E+01	1.90E-01	7.23E-01
Sodium Coceth-30 Sulfate	1.69E-02	1.09E+01	1.41E-02	4.27E-02
Sodium C12-13 Pareth Sulfate (A) / Sodium Laureth Sulfate (B)	1.64E-02	1.05E+01	1.37E-02	4.14E-02
Steareth-20	1.45E-02	7.12E+00	4.62E-03	1.37E-02
Co-polymer of PEG / Vinyl Acetate	1.91E-01	1.03E+01	7.78E-02	5.60E-01
2-Propylheptanol ethoxylated	1.44E-02	7.04E+00	4.57E-03	1.36E-02
Polyethylene Glycol	1.66E-01	9.00E+00	6.78E-02	4.89E-01

2- propenoic acid, homopolymer, sodium salt, sulfonated	6.04E-02	2.72E+01	1.08E-02	1.16E+00
Dimethiconol	2.08E-01	1.75E+00	2.59E-01	1.12E+00
Trideceth-7	1.20E-02	5.87E+00	3.81E-03	1.13E-02
Fatty alcohol alkoxylate	1.20E-02	5.87E+00	3.80E-03	1.13E-02
Laureth-7	1.15E-02	5.63E+00	3.65E-03	1.09E-02
Guar Hydroxypropyltrimonium Chloride	7.89E-01	2.48E+01	4.09E-03	4.08E-01
C15 Pareth-n	1.08E-02	5.28E+00	3.43E-03	1.02E-02
Laureth-3	1.00E-02	4.90E+00	3.18E-03	9.46E-03
PPG-12	1.27E-01	6.85E+00	5.16E-02	3.72E-01
PPG-9	1.20E-01	6.48E+00	4.88E-02	3.52E-01
Acrylic acid sodium salt polymer, sodium sulfonate terminated	4.07E-02	1.83E+01	7.29E-03	7.85E-01
C12-16 Pareth-n	8.47E-03	4.15E+00	2.69E-03	8.01E-03
Microcrystalline Cellulose	2.61E+00	9.95E+00	1.04E-01	3.95E-01
Hydrogenated Starch Hydrolysate	2.54E+00	9.92E+00	1.01E-01	3.94E-01
Corn Starch Modified	2.47E+00	9.67E+00	9.83E-02	3.84E-01
Copolymer of acrylic and sulphonic acids	3.58E-02	1.61E+01	6.40E-03	6.89E-01
PPG-34	9.70E-02	5.24E+00	3.95E-02	2.85E-01
PEG-6 Caprylic/Capric Glycerides	3.44E+00	1.73E+01	1.37E-01	6.90E-01
Ceteareth-25	7.10E-03	3.48E+00	2.26E-03	6.72E-03
PEG-150 Pentaerythrityl Tetrastearate	3.31E+00	1.67E+01	1.32E-01	6.64E-01
PPG-5-Ceteth-20	6.70E-03	3.28E+00	2.13E-03	6.34E-03
Sodium C12-15 Pareth-3 Sulfate	7.24E-03	4.65E+00	6.04E-03	1.83E-02
Trimethylsiloxysilicate	1.08E-01	9.10E-01	1.35E-01	5.85E-01
Alcohols, C12-16, ethoxylated, 7-16 EO	6.24E-03	3.06E+00	1.98E-03	5.90E-03
C9-11 Pareth-6	6.24E-03	3.06E+00	1.98E-03	5.90E-03
Shea Butter Glycereth-8 Esters	2.90E+00	1.46E+01	1.15E-01	5.82E-01
PPG-6	7.92E-02	4.28E+00	3.23E-02	2.32E-01
Modified Polycarboxylate	2.88E-02	1.30E+01	5.16E-03	5.56E-01
Phenylpropyl Ethyl Methicone	9.70E-02	8.16E-01	1.21E-01	5.25E-01
Maleic- acrylic acid copolymer sodiumsalt	2.68E-02	1.21E+01	4.80E-03	5.17E-01
Laureth-10	5.56E-03	2.73E+00	1.77E-03	5.26E-03
Simethicone	9.42E-02	7.93E-01	1.17E-01	5.10E-01
Polyquaternium-10	3.61E-01	1.14E+01	1.87E-03	1.87E-01
Steareth-4	5.13E-03	2.51E+00	1.63E-03	4.85E-03
Polyethylene Glycol MW >4100	6.64E-02	3.59E+00	2.70E-02	1.95E-01
Silicone Compound	8.32E-02	7.00E-01	1.04E-01	4.50E-01
Carbomer	2.34E-02	1.06E+01	4.19E-03	4.52E-01
Primary alcohol ethoxylate	4.86E-03	2.38E+00	1.55E-03	4.60E-03
Deceth-8	4.79E-03	2.35E+00	1.52E-03	4.53E-03
PEG-8 Propylheptyl Ether	4.79E-03	2.35E+00	1.52E-03	4.53E-03
Cellulose	1.43E+00	5.45E+00	5.68E-02	2.17E-01
Polyethylene Glycol MW <4100	5.94E-02	3.21E+00	2.42E-02	1.74E-01
Siloxanes and Silicones, di-Me, Me octyl, Me 2-phenylpropyl	7.21E-02	6.06E-01	8.97E-02	3.90E-01
PEG-80 Sorbitan Laurate	1.94E+00	9.76E+00	7.70E-02	3.88E-01
Sodium Starch Octenylsuccinate	1.17E+00	4.58E+00	4.66E-02	1.82E-01
Ceteareth-80	3.85E-03	1.89E+00	1.22E-03	3.64E-03
C9-11 Pareth-8	3.74E-03	1.84E+00	1.19E-03	3.54E-03
Acrylic acid maleic acid polymer	1.79E-02	8.05E+00	3.20E-03	3.45E-01

PEG-7 Propylheptyl Ether	3.43E-03	1.68E+00	1.09E-03	3.24E-03
Trideceth-10	3.37E-03	1.65E+00	1.07E-03	3.19E-03
PEG Copolymer	4.42E-02	2.39E+00	1.80E-02	1.30E-01
PEG-45M	4.37E-02	2.36E+00	1.78E-02	1.28E-01
Polycarboxylate, sodium salt	1.34E-02	6.04E+00	2.40E-03	2.58E-01
Polyethylenimine	1.90E-01	5.98E+00	9.86E-04	9.83E-02
Maltodextrin	7.94E-01	3.10E+00	3.16E-02	1.23E-01
PPG-1 Trideceth-6	2.54E-03	1.25E+00	8.08E-04	2.40E-03
Alkylethoxylate C9-11, 5.5EO	2.50E-03	1.22E+00	7.94E-04	2.36E-03
Ethoxylated Alcohol	2.50E-03	1.22E+00	7.94E-04	2.36E-03
PEG-75	3.26E-02	1.76E+00	1.33E-02	9.57E-02
PEG-33	3.14E-02	1.70E+00	1.28E-02	9.23E-02
Laureth-23	2.35E-03	1.15E+00	7.48E-04	2.22E-03
Acrylates/Steareth-20 Methacrylate Copolymer	1.12E-02	5.03E+00	2.00E-03	2.15E-01
Acrylates/C10-30 Alkyl Acrylate Crosspolymer	1.07E-02	4.81E+00	1.91E-03	2.06E-01
Zinc Coceth Sulfate	2.47E-03	1.59E+00	2.06E-03	6.24E-03
Alcohols, C12-14, ethoxylated, sulfates, sodium salts	2.41E-03	1.55E+00	2.01E-03	6.10E-03
Trideceth-6	2.15E-03	1.05E+00	6.83E-04	2.03E-03
Phenylpropyl Dimethicone	3.60E-02	3.03E-01	4.49E-02	1.95E-01
Hydroxypropyl Methylcellulose	6.29E-01	2.40E+00	2.50E-02	9.53E-02
Trideceth-12	1.97E-03	9.65E-01	6.26E-04	1.86E-03
PEG-4	2.54E-02	1.37E+00	1.03E-02	7.44E-02
C12-13 Pareth-n	1.87E-03	9.17E-01	5.94E-04	1.77E-03
Sodium polyaspartate	8.94E-03	4.03E+00	1.60E-03	1.72E-01
2-Propenoic acid, homopolymer, sodium salt	8.94E-03	4.03E+00	1.60E-03	1.72E-01
Acrylates/Steareth-20 Methacrylate Crosspolymer	8.69E-03	3.91E+00	1.55E-03	1.67E-01
Sodium Acrylates Copolymer	8.69E-03	3.91E+00	1.55E-03	1.67E-01
Tapioca Starch	5.38E-01	2.10E+00	2.14E-02	8.36E-02
PEG-80	2.33E-02	1.26E+00	9.49E-03	6.84E-02
Sodium Myreth Sulfate	1.83E-03	1.18E+00	1.53E-03	4.64E-03
Ethylene/propylene oxide copolymer	2.10E-02	1.13E+00	8.54E-03	6.15E-02
PEG-130 - PEG-150	2.10E-02	1.13E+00	8.54E-03	6.15E-02
Alcohols C12-14, ethoxylated (7EO)	1.54E-03	7.55E-01	4.89E-04	1.46E-03
C12-16 pareth-7	1.54E-03	7.55E-01	4.89E-04	1.46E-03
Pareth-7	1.54E-03	7.55E-01	4.89E-04	1.46E-03
Ethylene/MA Copolymer	7.21E-03	3.24E+00	1.29E-03	1.39E-01
Methacrylic acid and acrylic acid ester copolymer	7.21E-03	3.24E+00	1.29E-03	1.39E-01
No EU INCI name - Acrylic Copolymer	7.21E-03	3.24E+00	1.29E-03	1.39E-01
Polysorbate 60	6.64E-01	3.35E+00	2.64E-02	1.33E-01
Hydroxypropyl Guar Hydroxypropyltrimonium Chloride	9.28E-02	2.93E+00	4.82E-04	4.81E-02
Polyquaternium-37	9.05E-02	2.85E+00	4.70E-04	4.69E-02
Alkylethoxylate, C10-16, 10EO	1.25E-03	6.12E-01	3.97E-04	1.18E-03
C9-11 Pareth-9	1.25E-03	6.12E-01	3.97E-04	1.18E-03
Undeceth-40	1.25E-03	6.12E-01	3.97E-04	1.18E-03
Sodium Hydroxypropyl Starch Phosphate	3.51E-01	1.37E+00	1.40E-02	5.46E-02
PEG-4 Distearyl Ether	1.15E-03	5.62E-01	3.64E-04	1.08E-03
Steareth-21	1.12E-03	5.50E-01	3.57E-04	1.06E-03
Sodium Hydrolyzed Potato Starch Dodecenylsuccinate	3.38E-01	1.32E+00	1.34E-02	5.26E-02

Ceteareth-20	1.10E-03	5.42E-01	3.51E-04	1.05E-03
Acrylates/Beheneth-25 Methacrylate Copolymer	5.10E-03	2.30E+00	9.12E-04	9.83E-02
Magnesium Laureth Sulfate	1.19E-03	7.64E-01	9.93E-04	3.01E-03
Magnesium Laureth-8 Sulfate	1.19E-03	7.64E-01	9.93E-04	3.01E-03
Magnesium Oleth Sulfate	1.19E-03	7.64E-01	9.93E-04	3.01E-03
Sodium Laureth-8 Sulfate	1.19E-03	7.64E-01	9.93E-04	3.01E-03
Sodium Oleth Sulfate	1.19E-03	7.64E-01	9.93E-04	3.01E-03
PEG-135	1.40E-02	7.55E-01	5.69E-03	4.10E-02
Carboxymethylinulin	3.09E-01	1.21E+00	1.23E-02	4.80E-02
Poloxamer 407	1.33E-02	7.21E-01	5.43E-03	3.92E-02
Polyvinyl alcohol film	6.26E-02	3.75E-01	9.66E-03	5.80E-02
Thermal shrinkable PVOH film	6.26E-02	3.75E-01	9.66E-03	5.80E-02
Acrylates/PEG-10 Maleate/Styrene Copolymer	4.81E-03	2.17E+00	8.61E-04	9.27E-02
PEG-14M	1.23E-02	6.65E-01	5.02E-03	3.61E-02
Sodium polyitaconate	4.47E-03	2.01E+00	8.00E-04	8.61E-02
Poloxamer 124	1.16E-02	6.29E-01	4.74E-03	3.42E-02
C12-13 Pareth-3	8.65E-04	4.24E-01	2.75E-04	8.18E-04
Polyquaternium-2	5.91E-02	1.86E+00	3.07E-04	3.06E-02
PEG-9 Cocoglycerides	3.98E-01	2.00E+00	1.58E-02	7.97E-02
Acrylamidopropyltrimonium Chloride/Acrylamide Copolymer	5.84E-02	1.84E+00	3.03E-04	3.02E-02
Peg-30 - Peg-40	1.05E-02	5.66E-01	4.27E-03	3.08E-02
C13-15 Pareth-7	7.70E-04	3.77E-01	2.45E-04	7.28E-04
Ceteareth-15	7.70E-04	3.77E-01	2.45E-04	7.28E-04
Alcohol alkoxylate	7.48E-04	3.67E-01	2.38E-04	7.07E-04
Modified Fatty alcohol polyglycolether	7.48E-04	3.67E-01	2.38E-04	7.07E-04
Polyoxyethylene trimethyldecyl alcohol	7.48E-04	3.67E-01	2.38E-04	7.07E-04
PPG-5-Laureth-5	7.48E-04	3.67E-01	2.38E-04	7.07E-04
Hydroxypropyl starch phosphate	2.25E-01	8.81E-01	8.95E-03	3.50E-02
Acrylates Crosspolymer-4	3.38E-03	1.52E+00	6.05E-04	6.51E-02
PEG-23M	8.93E-03	4.82E-01	3.64E-03	2.62E-02
PEG-90 Glyceryl Isostearate	3.11E-01	1.57E+00	1.24E-02	6.23E-02
Triticum vulgare (wheat) starch	1.83E-01	7.14E-01	7.26E-03	2.84E-02
Polyacrylate-33	2.86E-03	1.29E+00	5.11E-04	5.51E-02
Polyquaternium-6	4.02E-02	1.27E+00	2.09E-04	2.08E-02
MIPA Laureth Sulfate	6.45E-04	4.15E-01	5.39E-04	1.63E-03
Sodium Trideceth Sulfate	6.45E-04	4.15E-01	5.39E-04	1.63E-03
Methicone	9.17E-03	7.71E-02	1.14E-02	4.96E-02
MIPA C12-15 Pareth Sulfate	5.95E-04	3.82E-01	4.96E-04	1.50E-03
PEG-10	6.99E-03	3.78E-01	2.85E-03	2.05E-02
PEG-90	6.99E-03	3.78E-01	2.85E-03	2.05E-02
Poloxalene	6.99E-03	3.78E-01	2.85E-03	2.05E-02
Starch	1.55E-01	6.04E-01	6.14E-03	2.40E-02
2-Propenoic Acid, Telomer with Sodium Hydrogen Sulfite, Sodium Salt	2.24E-03	1.01E+00	4.00E-04	4.31E-02
Polyacrylic Acid	2.24E-03	1.01E+00	4.00E-04	4.31E-02
PEG-2M	5.88E-03	3.18E-01	2.40E-03	1.73E-02
Phenyl Trimethicone	6.89E-03	5.80E-02	8.58E-03	3.73E-02
PEG-60 Almond Glycerides	1.81E-01	9.14E-01	7.21E-03	3.63E-02

C12-13 Pareth-6	3.74E-04	1.83E-01	1.19E-04	3.54E-04
Aziridine, homopolymer	2.37E-02	7.48E-01	1.23E-04	1.23E-02
PEG-180M	4.45E-03	2.40E-01	1.81E-03	1.31E-02
PEG-180	4.41E-03	2.38E-01	1.80E-03	1.30E-02
PEG-10 Olive Glycerides	1.55E-01	7.84E-01	6.18E-03	3.11E-02
PEG-200 Hydrogenated Glyceryl Cocoate	1.55E-01	7.84E-01	6.18E-03	3.11E-02
Acrylates/Palmeth-25 Acrylate Copolymer	1.50E-03	6.76E-01	2.69E-04	2.89E-02
C30-45 Alkyl Dimethicone	5.17E-03	4.35E-02	6.44E-03	2.80E-02
Laureth-5	2.99E-04	1.47E-01	9.50E-05	2.83E-04
C12-13 Pareth-23	2.96E-04	1.45E-01	9.40E-05	2.80E-04
PPG-3 myristyl ether	2.96E-04	1.45E-01	9.40E-05	2.80E-04
Divinyldimethicone/dimethicone copolymer	4.90E-03	4.12E-02	6.10E-03	2.65E-02
Polyquaternium-39	1.87E-02	5.89E-01	9.71E-05	9.68E-03
PEG- 30 - PEG-150	3.49E-03	1.89E-01	1.42E-03	1.03E-02
PEG-150	3.49E-03	1.89E-01	1.42E-03	1.03E-02
PEG-32	3.49E-03	1.89E-01	1.42E-03	1.03E-02
PEG-8	3.49E-03	1.89E-01	1.42E-03	1.03E-02
Sodium Styrene/Acrylates Copolymer	1.24E-03	5.59E-01	2.22E-04	2.39E-02
Acrylates/Ammonium Methacrylate Copolymer	1.24E-03	5.59E-01	2.22E-04	2.39E-02
Polyquaternium-47	1.78E-02	5.62E-01	9.26E-05	9.23E-03
Dimethiconol/Silsesquioxane Copolymer	4.36E-03	3.67E-02	5.43E-03	2.36E-02
Polyquaternium-68	1.76E-02	5.53E-01	9.12E-05	9.09E-03
Potato Starch Modified	7.69E-02	3.01E-01	3.06E-03	1.19E-02
Zea mays (corn) starch	7.69E-02	3.01E-01	3.06E-03	1.19E-02
Saccharide Isomerate	7.03E-02	2.75E-01	2.79E-03	1.09E-02
PEG-9	2.98E-03	1.61E-01	1.21E-03	8.73E-03
Laureth-16	2.24E-04	1.10E-01	7.13E-05	2.12E-04
PEG-120 Methyl Glucose Trioleate	9.07E-02	4.57E-01	3.60E-03	1.82E-02
Laureth-9	1.85E-04	9.08E-02	5.89E-05	1.75E-04
PVM/MA Copolymer	8.90E-04	4.01E-01	1.59E-04	1.71E-02
Polyquaternium-70	1.25E-02	3.95E-01	6.51E-05	6.49E-03
PPG-n	2.33E-03	1.26E-01	9.48E-04	6.83E-03
Polyquaternium-22	1.20E-02	3.78E-01	6.23E-05	6.21E-03
Starch Hydroxypropyltrimonium Chloride	1.17E-02	3.68E-01	6.07E-05	6.05E-03
Trimethylsiloxysilicate/Dimethicone Crosspolymer	2.77E-03	2.33E-02	3.45E-03	1.50E-02
Acrylic Acid/Acrylamidomethyl Propane Sulfonic Acid Copolymer	7.51E-04	3.38E-01	1.34E-04	1.45E-02
Polyacrylate-1 Crosspolymer	7.51E-04	3.38E-01	1.34E-04	1.45E-02
PEG-90M	2.02E-03	1.09E-01	8.21E-04	5.92E-03
PEI-2500	8.00E-03	2.52E-01	4.15E-05	4.14E-03
Potato Starch	3.18E-02	1.24E-01	1.26E-03	4.95E-03
Caprylyl Methicone	1.72E-03	1.45E-02	2.15E-03	9.32E-03
Polyquaternium-55	6.97E-03	2.20E-01	3.62E-05	3.61E-03
Cetyl Hydroxyethylcellulose	2.86E-02	1.09E-01	1.14E-03	4.33E-03
PG-Hydroxyethylcellulose cocodimonium chloride	5.30E-03	1.67E-01	2.75E-05	2.74E-03
Polyquaternium-28	5.30E-03	1.67E-01	2.75E-05	2.74E-03
Coceth-7	7.47E-05	3.66E-02	2.38E-05	7.07E-05
Macrogol Lauryl Ether (4)	7.47E-05	3.66E-02	2.38E-05	7.07E-05

Oleth-20	7.47E-05	3.66E-02	2.38E-05	7.07E-05
PPG-1-PEG-9 Lauryl Glycol Ether	7.47E-05	3.66E-02	2.38E-05	7.07E-05
Polyquaternium-52	5.02E-03	1.58E-01	2.61E-05	2.60E-03
Polyquaternium-16	3.62E-03	1.14E-01	1.88E-05	1.87E-03
Polyquaternium-4	3.62E-03	1.14E-01	1.88E-05	1.87E-03
Polyquaternium-76	3.35E-03	1.06E-01	1.74E-05	1.73E-03
Modified Guar Hydroxypropyltrimonium Chloride	1.67E-03	5.28E-02	8.70E-06	8.67E-04
Polyacrylamidopropyltrimonium chloride	1.67E-03	5.28E-02	8.70E-06	8.67E-04
Polyquaternium-30	1.67E-03	5.28E-02	8.70E-06	8.67E-04

10. Comparison to exposure data from the literature

10a. Surface water

Supplemental Data 10a: Predicted and measured environmental concentrations (PEC and MEC) of polymers in surface water from the literature and present study.
Note that where concentrations were measured in wastewater effluent (without direct surface water measurements), a dilution factor of 10 was applied in the present study to obtain comparable concentration estimates for surface water, but values are still recorded as MEC below.

Polymer(s)	Measured or predicted	Value/ µg L^{-1}	Region	Notes	Relevant PEC_{SW} values for individual polymers within groups from the present study/ µg L^{-1}	Reference
				**Polycarboxylates**		
Polycarboxylates	PEC	2-915	United Kingdom	PEC_{SW} for the entire group of polycarboxylate polymers found in UK household products in the present study.	n/a	This study
PAA	PEC	70-570	United States	PEC for river, 90th percentiles, for PAA from cleaning products in the US. 70 µg L^{-1} = mean flow, 570 µg L^{-1} = low flow.	Present study total polyacrylic acid (PAA): 0.5-222	DeLeo *et al.* 2020
PAA-MA	PEC	20-130	United States	PEC for river, 90th percentiles, for PAA-MA from cleaning products in the US. 20 µg L^{-1} = mean flow, 130 µg L^{-1} = low flow.	Present study total acrylic acid-maleic acid copolymer (PAA-MA): 0.4-166	DeLeo *et al.* 2020
PAA	PEC	43-110	Europe	PEC for water for PAA used in European household cleaning products. 43 µg L^{-1} = regional PEC, 110 µg L^{-1} = local PEC.	Present study total PAA: 0.5-222	HERA 2014a, 2014b

PAA-MA	PEC	35-49	Europe	PEC for water for PAA-MA used in European household cleaning products. 35 µg L^{-1} = regional PEC, 49 µg L^{-1} = local PEC.	Present study total PAA-MA: 0.4-166	HERA 2014a, 2014b
PAA-MA	PEC	30	Europe	PEC_{SW} for PAA-MA 70,000.	Present study total PAA-MA: 0.4-166. Note that molecular weight information was not obtainable in the present study and so a more specific comparison is not possible.	ECETOC 1993
			**Alcohol ethoxylate salts**			
Alcohol ethoxylate salts	PEC	1-731	United Kingdom	PEC_{SW} for the entire group of alcohol ethoxylate salt polymers found in UK household products in the present study.	All members of this group are ethoxy-sulfates and are comparable to literature data as a summed mixture, with literature values also typically giving a range of C_n and EO. EO is not reported for most members of the group in the present study. Note that further sub-division is not possible as identified polymers will contain a range of EO (and often C) chain lengths, and the distribution cannot be calculated from the available data.	This study
Alcohol ethoxylate sulfates	PEC	0.42-54.87	Canada	PEC range for 100th percentile to 10th percentile. C = 10-16, EO = unspecified.	Present study total group: 1-731	ECCC 2019

Alcohol ethoxylate sulfates	PEC	3.663-22.79	Europe	Simulation test PEC for surface water. 3.663 µg L^{-1} = regional PEC, 22.79 µg L^{-1} = local PEC. C = 12-18, EO = 0-8 or average 2.7.	Present study total group: 1-731	HERA 2004
Alcohol ethoxylate sulfates	MEC	5.7-10.3	United States	5.7 µg L^{-1} = concentration in river water from selected-ion recording (mass spectrometry) for C = 14,15 and EO = 0-8. 10.3 µg L^{-1} = concentration in river water from full scan (mass spectrometry) for C = 12-15 and EO = 0-8.	Present study total group: 1-731	Popenoe *et al.* 1994
Alcohol ethoxylate sulfates	MEC	0.4-5.8	United States	x10 dilution factor applied to value for alkyl ethoxylate sulfates present in WWTP effluent. Includes activated sludge and trickling filter. Sum of values for C = 10-18, EO = 1-10.	Present study total group: 1-731	McAvoy *et al.* 1998
Alcohol ethoxylate sulfates	PEC	0.93-5.3	United States	PEC values for 90% river miles < concentration. 0.93 µg L^{-1} = mean flow, 5.3 µg L^{-1} = low flow. C = 12-18, EO = 0-8.	Present study total group: 1-731	Cowan-Ellsberry *et al.* 2014
Alcohol ethoxylate sulfates	MEC	3.2	Spain	x10 dilution factor applied to value for alcohol ethoxylate sulfates measured in WWTP effluent in Spain (before electron beam irradiation treatment). Sum of values for C = 10-18, EO = 1-10.	Present study total group: 1-731	Petrovic *et al.* 2007

Alcohol ethoxylate sulfates	MEC	2.5-2.9	Spain	Includes concentrations for seawater and estuary in Spain. Sum of values for C = 12,14 and EO = 1-11, and C = 16 and EO = 1-10.	Present study total group: 1-731	Lara-Martín *et al.* 2006
Alcohol ethoxylate sulfates	MEC	0.3-1.2	Netherlands	x10 dilution factor applied to value for alcohol ethoxysulfates present in WWTP effluent from the Netherlands. Sum of values for C = 12-15 (both linear and branched), EO = 0-8.	Present study total group: 1-731	Matthijs *et al.* 1999
Alcohol ethoxylate sulfates and alkyl sulfates	MEC	0.01-0.226	United States	Includes river water concentrations upstream, downstream, far downstream, and at outfall of WWTP, for both alcohol ethoxylate sulfates and alkyl sulfates (NB it is likely that the total alcohol ethoxylate salt PEC from the present study also includes non-ethoxylated alcohol).	Present study total group: 1-731	Sanderson *et al.* 2006
Alcohol ethoxylate sulfates	MEC	< 0.222-0.19	Germany	x10 dilution factor applied to value for concentration of alcohol ethoxylate sulfates in WWTP effluent. C = 12,14 and EO = 0-9.	Present study total group: 1-731	Freeling *et al.* 2019
Alcohol ethoxylate sulfates and alkyl sulfates	MEC	< 0.1	Germany	x10 dilution factor applied to value for WWTP effluent concentration of alcohol ethoxylate sulfates. C = 11-18, EO = unspecified.	Present study total group: 1-731	Schröder *et al.* 1999

			**Alcohol alkoxylates**			
Alcohol alkoxylates	PEC	0.8-370	United Kingdom	PEC_{SW} for the entire group of alcohol alkoxylate polymers found in UK household products in the present study.	Sum of PEC_{SW} for ethoxy-ethers with C = 9-18 and EO ≤ 21 is used for all comparisons below (deemed generally representative of EO ranges given in literature). Note that further subdivision and direct comparisons are not possible as identified polymers will contain a range of EO (and often C) chain lengths, and the distribution cannot be calculated from the available data. Values are also reported for chain length ranges in literature.	This study
Alcohol ethoxylates	MEC	0.8-50.9	United States	x10 dilution factor applied to value for alkyl ethoxylates present in WWTP effluent. Includes activated sludge and trickling filter. Sum of values for C = 12-15, average EO assumed to be 9.	Present study sum alcohol ethoxylates with C = 9-18 and EO ≤ 21: 0.4-198	McAvoy *et al.* 1998
Alcohol ethoxylates	MEC	2-37	United States	Includes concentration both upriver and downriver of WWTP in the US (includes both activated sludge and trickling filter). C = 12-15, average EO assumed to be 9.	Present study sum alcohol ethoxylates with C = 9-18 and EO ≤ 21: 0.4-198	Fendinger *et al.* 1995

Alcohol ethoxylates	MEC	0.028-31.6	United States	x10 dilution factor applied to value for alkyl ethoxylates present in WWTP effluent. Includes activated sludge and trickling filter. Includes novel data and data from previous studies with corrections applied. Sum of values for C = 12-15, EO = 2-18 or average EO = 3.8.	Present study sum alcohol ethoxylates with C = 9-18 and EO ≤ 21: 0.4-198	McAvoy *et al.* 2006
Alcohol ethoxylates	MEC	<0.02-31	Japan	Measured concentration in surface water, Japan. C = 12-15, EO = 2-20.	Present study sum alcohol ethoxylates with C = 9-18 and EO ≤ 21: 0.4-198	Miura *et al.* 2008
Alcohol ethoxylates	MEC	7.8-27.9	Spain	Measured concentration in river water, Spain. Sum of values for C = 12,14,16 and EO = 1-20.	Present study sum alcohol ethoxylates with C = 9-18 and EO ≤ 21: 0.4-198	Cantero *et al.* 2005
Alcohol ethoxylates	PEC	0.06-16.76	Canada	PEC range for 100th percentile to 10th percentile. C = 6-16, EO = unspecified (but stated as typically 3 to 10-12).	Present study sum alcohol ethoxylates with C = 9-18 and EO ≤ 21: 0.4-198	ECCC 2019
Alcohol ethoxylates	PEC	0.761-9.93	United States	PEC at WWTP outfall for alcohol ethoxylates. 0.761 µg L^{-1} = mean flow, 9.93 µg L^{-1} = low flow.	Present study sum alcohol ethoxylates with C = 9-18 and EO ≤ 21: 0.4-198	Sanderson *et al.* 2013
Alcohol ethoxylates	PEC	0.89-5.3	United States	PEC values for 90% river miles < concentration. 0.89 µg L^{-1} = mean flow, 5.3 µg L^{-1} = low flow. C = 8-18, EO = 3-12.	Present study sum alcohol ethoxylates with C = 9-18 and EO ≤ 21: 0.4-198	Cowan-Ellsberry *et al.* 2014

Alcohol ethoxylates	MEC	0.18-1.3	Netherlands	x10 dilution factor applied to value for alcohol ethoxylates present in WWTP effluent from the Netherlands. Sum of values for C = 12-15, EO = 2-18.	Present study sum alcohol ethoxylates with C = 9-18 and EO ≤ 21: 0.4-198	Matthijs *et al.* 1999
Alcohol ethoxylates	MEC	0.1-1.2	Spain	Includes concentrations for seawater and estuary in Spain. Sum of values for C = 12,14 and EO = 1-17, and C = 16 and EO = 1-16.	Present study sum alcohol ethoxylates with C = 9-18 and EO ≤ 21: 0.4-198	Lara-Martín *et al.* 2006
Alcohol ethoxylates	MEC	0.24-1.579	United States	Includes river water concentrations upstream, downstream, far downstream, and at outfall of WWTP. Values include alcohol ethoxylate and non-ethoxylated fatty alcohol. C = 12-18, EO = 0-15.	Present study sum alcohol ethoxylates with C = 9-18 and EO ≤ 21: 0.4-198	Sanderson *et al.* 2013
Alcohol ethoxylates	MEC	0.096-2.271	Europe, Canada	x10 dilution factor applied to value for alcohol ethoxylates present in WWTP effluent in Europe and Canada (mostly activated sludge, but includes values for a trickling filter and a rotating biological contactor). Sum of values for C = 12-18, EO = 0-18.	Present study sum alcohol ethoxylates with C = 9-18 and EO ≤ 21: 0.4-198	Eadsforth *et al.* 2006
Alcohol ethoxylates	PEC	1.01	Europe	Total local dissolved PEC for C = 8-18 and EO = 0-22, derived from monitoring data for C = 12-18 and EO = 0-18.	Present study sum alcohol ethoxylates with C = 9-18 and EO ≤ 21: 0.4-198	HERA 2009

Alcohol ethoxylates	MEC	0.1051-0.9354	Germany	x10 dilution factor applied to value for alcohol ethoxylates present in WWTP effluent. Sum of values for C = 8-18, EO = 1-20.	Present study sum alcohol ethoxylates with C = 9-18 and EO ≤ 21: 0.4-198	Freeling *et al.* 2019
Alcohol ethoxylates	MEC	0.364	United States	x10 dilution factor applied to value for alcohol ethoxylates present in WWTP effluent from the United States. Sum of values for C = 12-18, EO = 0-18.	Present study sum alcohol ethoxylates with C = 9-18 and EO ≤ 21: 0.4-198	Morrall *et al.* 2006
Alcohol ethoxylates	MEC	0.25	Spain	x10 dilution factor applied to value for alcohol ethoxylates present in WWTP effluent (before electron beam irradiation treatment). Sum of values for C = 10-18, EO = 1-10.	Present study sum alcohol ethoxylates with C = 9-18 and EO ≤ 21: 0.4-198	Petrovic *et al.* 2007
Alcohol ethoxylates	MEC	0.0167	United States	x10 dilution factor applied to value for alcohol ethoxylates present in New York WWTP effluent. C = 12-18, EO = 1-3.	Present study sum alcohol ethoxylates with C = 9-18 and EO ≤ 21: 0.4-198	Lara-Martin *et al.* 2014
Alcohol ethoxylates	MEC	<0.011	United States	Concentration in New York seawater. Sum of values for C = 12-18 and EO = 1-8.	Present study sum alcohol ethoxylates with C = 9-18 and EO ≤ 21: 0.4-198	Lara-Martin *et al.* 2011
			**Polyol ethoxylate esters**			
Polyol ethoxylate esters	PEC	67-337	United Kingdom	PEC_{SW} for the entire group of polyol ethoxylate ester polymers found in UK household products in the present study.	Literature data not available for comparison.	This study

**Polyquaterniums**						
Polyquaterniums	PEC	5-142	United Kingdom	PEC_{SW} for the entire group of polyquaternium polymers found in UK household products in the present study.	n/a	This study
Polyquaterniums	PEC	0.039-0.46	Australia	Predicted environmental concentration for polyquaterniums in Australia across a range of import/manufacture volumes (20-80 tonnes). 0.46 µg L^{-1} = 21% WWTP removal, 80 tonnes, for all polyquaternium use in Australia; 0.039 µg L^{-1} = 73% WWTP removal (considered likely by the author of the study to be the upper limit for the K_0 values of polyquaterniums), 20 tonnes, of polyquaterniums.	The authors noted that there was no way to estimate the mixture of polyquaterniums present and that the data reflect a total estimate, and thus data from this study should be compared to the total group PEC_{SW} of the present study (5-142).	Cumming 2008
Polyquaternium-68	PEC	0.72	Australia	Predicted river concentration of polyquaternium-68 in Australia assuming WWTP removal of 90% and dilution factor of x1.	Present study polyquaternium-68: 0.02-0.6	NICNAS 2009
**Starch and derivatives**						
Starch and derivatives	PEC	31-123	United Kingdom	PEC_{SW} for the entire group of starch and starch derivative polymers found in UK household products in the present study.	Literature data not available for comparison.	This study

**Polyethers and copolymers**						
Polyethers and copolymers	PEC	2-90	United Kingdom	PEC_{SW} for the entire group of polyether polymers and copolymers found in UK household products in the present study.	n/a	This study
Polypropylene glycols	MEC	212	Poland	Total concentration of polypropylene glycol (PPG) of chain lengths from 10-17 monomer units in water from the River Warta.	Present study sum PPG-6 to PPG-26: 0.7-38.<br>Note this comparison is for reference only; direct comparison is not possible as each PPG identified in the present study will contain a distribution of chain lengths, with the actual composition not possible to derive from available data.	Rychłowska *et al.* 2003
Polyethylene oxides	MEC	< LoD-11	Germany	Concentration range across 18 surface water samples from Germany. Concentrations in WWTP effluent reached up to 20 µg L^{-1}.	Present study total PEG: 0.6-30. The literature study in question quantifies PEG/PEO independently of molecular weight and thus the sum of all PEG identified in the present study is most suitable for comparison.	Pauelsen *et al.* 2023

Polypropylene glycols	MEC	0.4-1.8	Denmark	x10 dilution factor applied to total measured concentration of polypropylene glycol homologues (3-12 monomer units) in the effluent of two WWTPs in Denmark. Note that ethylene oxide/propylene oxide copolymers were also detected, but not quantified.	Present study sum PPG-6 to PPG-12: 0.3-18.<br>Note this comparison is for reference only; direct comparison is not possible as each PPG identified in the present study will contain a distribution of chain lengths, with the actual composition not possible to derive from available data. For example, PPG-12 (average length) in the present study is also likely to contain a range of chain lengths >12.	Tisler *et al.* 2021
Polyethylene glycols	MEC	0.15-0.74	Germany	x10 dilution factor applied to rough estimate of total polyethylene glycol from 4 to 44 monomer units detected in WWTP effluent. Values are “rough estimates” – semi-quantified based on polyethylene glycol standard of 4 monomer units.	Present study sum PEG-4 to PEG-40: 0.08-5.<br>Note this comparison is for reference only; direct comparison is not possible as each PEG identified in the present study will contain a distribution of chain lengths, with the actual composition not possible to derive from available data. For example, PEG-40 (average length) in the present study is also likely to contain a range of chain lengths >40.	Freeling *et al.* 2019

Polyethylene glycols	MEC	0.63	United States	Concentration in New York seawater for PEG-4 to PEG-13.	Present study sum PEG-4 to PEG-10: 0.04-2. Note this comparison is for reference only; direct comparison is not possible as each PEG identified in the present study will contain a distribution of chain lengths, with the actual composition not possible to derive from available data. For example, PEG-10 (average length) in the present study is also likely to contain a range of chain lengths >10.	Lara-Martin *et al.* 2011
Polyethylene glycols	MEC	0.123-0.1606	United States	x10 dilution factor applied to value for polyethylene glycols (assumed PEG-4 to PEG-13) present in New York WWTP effluent.	Present study sum PEG-4 to PEG-10: 0.04-2. Note this comparison is for reference only; direct comparison is not possible as each PEG identified in the present study will contain a distribution of chain lengths, with the actual composition not possible to derive from available data. For example, PEG-10 (average length) in the present study is also likely to contain a range of chain lengths >10.	Lara-Martin *et al.* 2011; Lara-Martin *et al.* 2014
**Cellulose and derivatives**						
Cellulose and derivatives	PEC	16-60	United Kingdom	PEC_{SW} for the entire group of cellulose and cellulose derivative polymers found in UK household products in the present study.	Literature data not available for comparison.	This study

**Polyvinyl alcohol**						
Polyvinyl alcohol	PEC	3-19	United Kingdom	PEC_{SW} for the polyvinyl alcohol polymers found in UK household products in the present study.	Literature data not available for comparison.	This study
**Silicones**						
Silicones	PEC	1-12	United Kingdom	PEC_{SW} for the entire group of silicone polymers found in UK household products in the present study.	n/a	This study
Polydimethylsiloxane	MEC	<5-7	United States	Concentration of polydimethylsiloxane in receiving water. All but one sample were below the limit of detection (5 µg L^{-1}).	Present study sum dimethicone (=polydimethylsiloxane) and simethicone (=polydimethylsiloxane + SiO_2): 0.8-7.	Fendinger *et al.* 1997
Linear siloxanes (L3-L14)	MEC	0.01687-0.2683	Greece	x10 dilution factor applied to values for linear siloxanes (sum of minimum and maximum values for L3-L14) present in WWTP effluent in Greece. Cyclic siloxanes were not included due to the fact that these were excluded from the silicones group of the present study.	Present study sum dimethicone and simethicone: 0.8-7. Note this comparison is for reference only, as molecular weight information were not obtainable in the present study but polymers are expected to be significantly larger than those assessed in this literature study.	Bletsou *et al.* 2013

Linear siloxanes (L5-L14)	MEC	0.013243-0.12258	China	x10 dilution factor applied to values for linear siloxanes (sum of minimum and maximum values for L5-L14) present in WWTP effluent in China. Cyclic siloxanes were not included due to the fact that these were excluded from the silicones group of the present study.	Present study sum dimethicone and simethicone: 0.8-7. Note this comparison is for reference only, as molecular weight information were not obtainable in the present study but polymers are expected to be significantly larger than those assessed in this literature study.	Li *et al.* 2016

10b. Soil

Supplemental Data 10b: Predicted and measured environmental concentrations (PEC and MEC) of polymers in soil from the literature and present study.

Polymer	Measured or predicted	Value/ mg kg^{-1}	Region	Notes	Relevant PEC_{SOIL} values for individual polymers within groups from the present study/ mg kg^{-1}	Reference
				**Polycarboxylates**		
Polycarboxylates	PEC	0.4-39	United Kingdom	PEC_{SOIL} for the entire group of polycarboxylate polymers found in UK household products in the present study.	n/a	This study
PAA	PEC	0.47-4.37	Europe	PEC for soil for PAA used in detergents. 4.37 mg kg^{-1} wwt = local PEC, 0.47 mg kg^{-1} wwt = regional PEC.	Present study total PAA: 0.09-9	HERA 2014a, 2014b
PAA-MA	PEC	26.8-35.2	Europe	PEC for soil for PAA-MA used in detergents. 35.2 mg kg^{-1} wwt = regional PEC, 26.8 mg kg^{-1} wwt = local PEC.	Present study total PAA-MA: 0.07-7	HERA 2014a, 2014b
PAA-MA	PEC	6	Europe	PEC_{SOIL} for PAA-MA 70,000, in mg kg^{-1} y^{-1} (comparable to values given in the present study which were calculated for 1 year of sludge application).	Present study total PAA-MA: 0.07-7. Note that molecular weight information was not obtainable in the present study and so a more specific comparison is not possible.	ECETOC 1993

**Alcohol ethoxylate salts**						
Alcohol ethoxylate salts	PEC	1-3	United Kingdom	PEC_{SOIL} for the entire group of alcohol ethoxylate salt polymers found in UK household products in the present study.	All members of this group are ethoxy-sulfates and are comparable to literature data as a summed mixture, with literature values also giving a range of C_n and EO. EO is not reported for most members of the group in the present study. Note that further sub-division is not possible as identified polymers will contain a range of EO (and often C) chain lengths, and the distribution cannot be calculated from the available data.	This study
Alcohol ethoxysulfates	PEC	0.00166-0.0128	Europe	Simulation test PEC for agricultural soil. 0.0128 mg kg^{-1} wwt = local PEC, 0.00166 mg kg^{-1} wwt = local PEC with 87% anaerobic degradation. Sum of values for C = 12-18 and EO 0-8 or average 2.7.	Present study total group: 1-3	HERA 2004
**Alcohol alkoxylates**						
Alcohol alkoxylates	PEC	0.2-0.7	United Kingdom	PEC_{SOIL} for the entire group of alcohol alkoxylate polymers found in UK household products in the present study.	n/a	This study

Alcohol ethoxylates	PEC	0.24	Europe	PEC for soil in mg kg^{-1} wwt. Sum of values for C = 8-18 and EO = 0-22.	Present study sum alcohol ethoxylates with C = 9-18 and EO < 21: 0.2-0.6. Note that further subdivision is not possible as identified polymers will contain a range of EO and C chain lengths, and the distribution cannot be calculated from the available data.	HERA 2009
			**Polyol ethoxylate esters**			
Polyol ethoxylate esters	PEC	3-13	United Kingdom	PEC_{SOIL} for the entire group of polyol ethoxylate ester polymers found in UK household products in the present study.	Literature data not available for comparison.	This study
			**Polyquaterniums**			
Polyquaterniums	PEC	0.02-2	United Kingdom	PEC_{SOIL} for the entire group of polyquaternium polymers found in UK household products in the present study.	n/a	This study
Polyquaternium-68	PEC	0.0055-0.055	Australia	Predicted soil concentration of polyquaternium-68 in Australia from irrigation using WWTP effluent. 0.0055 mg kg^{-1} = 1 year of irrigation, 0.055 mg kg^{-1} = 10 years under repeated irrigation.	Present study polyquaternium-68: 0.00009-0.009	NICNAS 2009

**Starch and derivatives**						
Starch and derivatives	PEC	1-5	United Kingdom	PEC_{SOIL} for the entire group of starch and starch derivative polymers found in UK household products in the present study.	Literature data not available for comparison.	This study
**Polyethers and copolymers**						
Polyethers and copolymers	PEC	0.7-5	United Kingdom	PEC_{SOIL} for the entire group of polyether polymers and copolymers found in UK household products in the present study.	Literature data not available for comparison.	This study
**Cellulose and derivatives**						
Cellulose and derivatives	PEC	0.6-2	United Kingdom	PEC_{SOIL} for the entire group of cellulose and cellulose derivative polymers found in UK household products in the present study.	Literature data not available for comparison.	This study
**Polyvinyl alcohol**						
Polyvinyl alcohol	PEC	0.5-3	United Kingdom	PEC_{SOIL} for the polyvinyl alcohol polymers found in UK household products in the present study.	Literature data not available for comparison.	This study
**Silicones**						
Silicones	PEC	2-8	United Kingdom	PEC_{SOIL} for the entire group of silicone polymers found in UK household products in the present study.	n/a	This study

Polydimethylsiloxane	MEC	<0.41-10.4	United States	Concentration of polydimethylsiloxane measured in sludge-amended soil.	Present study sum dimethicone and simethicone: 1-4	Fendinger *et al.* 1997

References

A.I.S.E. 2019. A.I.S.E. Fact sheet January 2019: Compaction of household laundry detergents has enabled significant environmental savings. Available from: https://www.aise.eu/library/publications.aspx. International Association for Soaps, Detergents and Maintenance Products, Brussels.

Alonso-López O, López-Ibáñez S, Beiras R. 2021. Assessment of Toxicity and Biodegradability of Poly(vinyl alcohol)-Based Materials in Marine Water. *Polymers* 13. DOI: https://doi.org/10.3390/polym13213742.

Antwerpen W, Schindler H, Reinhardt G, inventors. Clariant Produkte Deutschland GmbH, assignee. 1994. Use of polyvinyl alcohols as detergent additive. European Patent Office EP 0 584 736 A1. Available from: https://patents.google.com/patent/EP0584736A1

Arfsten DP, Burton DT, Fisher DJ, Callahan J, Wilson CL, Still KR, Spargo BJ. 2004. Assessment of the aquatic and terrestrial toxicity of five biodegradable polymers. *Environmental Research* 94:198-210. DOI: https://doi.org/10.1016/S0013-9351(03)00087-2.

Arisandy C, Schmidt K, Leyrer RJ, inventors. BASF SE (DE), assignee. 2014. Use of a copolymer as thickener in liquid detergents having lower graying tendency. United States. US 8,865,639 B2. Available from: https://patents.google.com/patent/US8865639

Astolfi R, Leopoldino SR, Oura EM, Shafer GL, Yarovoy YK, inventors. Unilever PLC, N.V., Conopco Inc., D/B/A Unilever, assignee. 2017. Fatty acid soap bars prepared from oil stock of low iv comprising potassium soap. World Intellectual Property Organization WO 2017/129472 A1. Available from: https://patents.google.com/patent/WO2017129472A1/

Baixas Veiga E, Rosas Girones A, Smith B, inventors. Henkel Iberica SA, assignee. 1994. Bleaching composition containing alkaline hypochlorite and process for its manufacture. European Patent Office EP 0 340 371 B1. Available from: https://patents.google.com/patent/EP0340371B1/

Barrios JJ, Burakov D, Castillo-Bucci C, Henao-Cano U, Quadir M, inventors. L'Oreal, assignee. 2008. Cosmetic hair compositions containing metal-oxide layered pigments and functionalized metal-oxide layered pigments and methods of use. European Patent Office EP 1 997 472 A1. Available from: https://patents.google.com/patent/EP1997472A1/

Batchelor SN, Bird JM, inventors. Conopco Inc., assignee. 2015. Liquid laundry composition. United States. US 8,946,139 B2. Available from: https://patents.google.com/patent/US8946139

Beagle CA, Scherr EM, Taha RA, Knorr JR, inventors. Colgate-Palmolive Company, assignee. 1999. Laundry detergent compositions containing lipase and soil release polymer. United States. US 5,866,525 A. Available from: https://patents.google.com/patent/US5866525

Bennett J, McKee A, Parry A, inventors. Unilever PLC, assignee. 2012. Aqueous concentrated laundry detergent compositions. European Patent Office EP 2 522 714 A1. Available from: https://patents.google.com/patent/EP2522714A1/

Bergmann W, inventor. Helene Curtis, Inc., assignee. 1994. Conditioning shampoo composition and method of preparing and using the same. United States. US 5,275,761 A. Available from: https://patents.google.com/patent/US5275761

Bletsou AA, Asimakopoulos AG, Stasinakis AS, Thomaidis NS, Kannan K. 2013. Mass Loading and Fate of Linear and Cyclic Siloxanes in a Wastewater Treatment Plant in Greece. *Environmental Science & Technology* 47:1824-1832. DOI: https://doi.org/10.1021/es304369b.

Bolich REJ, Williams TB, inventors. The Procter & Gamble Company, assignee. 1988. Shampoo compositions containing nonvolatile silicone and xanthan gum. United States. US 4,788,006 A. Available from: https://patents.google.com/patent/US4788006

Borne J, inventor. The Procter & Gamble Company, assignee. 2012. Liquid detergent composition for improved grease cleaning comprising an alkoxylated polyethyleneimine polymer. United States. US 8,168,005 B2. Available from: https://patents.google.com/patent/US8168005

Boutique J-P, Delplancke PFA, Wagner R, Butts MD, Genovese SE, Scialla S, inventors. The Procter & Gamble Company, assignee. 2008. Liquid laundry detergent comprising a cationic silicone polymer and a coacervate phase forming cationic polymer. United States. US 7,439,217 B2. Available from: https://patents.google.com/patent/US7439217

Cantero M, Rubio S, Pérez-Bendito D. 2005. Determination of non-ionic polyethoxylated surfactants in wastewater and river water by mixed hemimicelle extraction and liquid chromatography–ion trap mass spectrometry. *Journal of Chromatography A* 1067:161-170. DOI: https://doi.org/10.1016/j.chroma.2004.11.017.

Carew PS, Manley R, Wire SL, inventors. Unilever PLC, Unilever NV, Hindustan Lever Ltd., assignee. 2003. Hair conditioning compositions. World Intellectual Property Organization WO 2003/000205 A1. Available from: https://patents.google.com/patent/WO2003000205A1/

Casteel S, Hartan H-G, Philippsen-Neu E, Poeschmann R, inventors. Stockhausen GmbH & Co. KG, assignee. 2001. Compacted granulate, process for making same and use as disintegrating agent for pressed detergent tablets, cleaning agent tablets for dishwashers, water softening tablets or scouring salt tablets. United States. US 6,221,832 B1. Available from: https://patents.google.com/patent/US6221832

Cermenati L, Tomarchio V, inventors. The Procter & Gamble Company, assignee. 2006. Hard surface cleaning composition comprising a bleach, acid, and silicone glycol polymer. United States. US 6,992,053 B2. Available from: https://patents.google.com/patent/US6992053

Charles JD, inventor. Dunlop Detergents LLC, assignee. 2014. Detergent compositions comprising a polydimethylsiloxane on sodium acetate foam control agent and methods of making. United States. US 8,822,398 B2. Available from: https://patents.google.com/patent/US8822398

Cheung TW, Costa B, inventors. Reckitt Benckiser Inc., assignee. 2003. Thickened toilet bowl cleaner. Canada. World Intellectual Property Organization and Canadian Intellectual Property Office WO 03/020863 A1, CA 2 452 962 C. Available from: https://patents.google.com/patent/CA2452962C/

Chun KW, Theiler RF, Baumgarten MI, Gabriel R, inventors. Unilever PLC, Unilever NV, assignee. 1992. Machine dishwashing detergent tablets. European Patent Office EP 0 481 547 A1. Available from: https://patents.google.com/patent/EP0481547A1/

Coffindaffer TW, Schrader EM, inventors. The Procter & Gamble Company, assignee. 1998. Conditioning shampoo compositions containing emulsion polymerized polymers World Intellectual Property Organization WO 1998/018434 A1. Available from: https://patents.google.com/patent/WO1998018434A1/

Conklin JR, inventor. The Dow Chemical Company, assignee. 1991. Use of low-viscosity grades of cellulose ethers as lather-enhancing additives. World Intellectual Property

Organization WO 91/13138, WO 1991/013138 A1. Available from: https://patents.google.com/patent/WO1991013138A1/
Corominas F, Beelen L, Akalay M, inventors. The Procter & Gamble Company, assignee. 2013. Methods for producing liquid detergent products. World Intellectual Property Organization WO 2013/128431 A2. Available from: https://patents.google.com/patent/WO2013128431A2/
Cowan-Ellsberry C, Belanger S, Dorn P, Dyer S, McAvoy D, Sanderson H, Versteeg D, Ferrer D, Stanton K. 2014. Environmental Safety of the Use of Major Surfactant Classes in North America. *Critical Reviews in Environmental Science and Technology* 44:1893-1993. DOI: https://doi.org/10.1080/10739149.2013.803777.
Cumming J. 2008. Environmental Fate, Aquatic Toxicology and Risk Assessment of Polymeric Quaternary Ammonium Salts from Cosmetic uses (PhD Thesis). Griffith University, Queensland, Australia.
DeLeo PC, Summers H, Stanton K, Lam MW. 2020. Environmental risk assessment of polycarboxylate polymers used in cleaning products in the United States. *Chemosphere* 258:127242. DOI: https://doi.org/10.1016/j.chemosphere.2020.127242.
Della Noce G, inventor. Rohm and Haas Company, assignee. 2016. Laundry detergent containing amine additives. World Intellectual Property Organization WO 2016/064968 A1. Available from: https://patents.google.com/patent/WO2016064968A1/
Demitz M, Köhler M, Saladin S, Mahadeshwar A, Wendicke S, inventors. Beiersdorf AG, assignee. 2009. Silicone-free conditioners for keratin fibers. German Patent and Trademark Office DE 10 2008 030 131 A1. Available from: https://patents.google.com/patent/DE102008030131A1/
Demson R, Dalton J, inventors. The Dial Corporation, assignee. 2004. Translucent soap bar composition and method of making the same. Canadian Intellectual Property Office CA 2 436 822 A1. Available from: https://patents.google.com/patent/CA2436822A1/
Depoot KJM, De Buzzaccarini F, Billiauw JJM-L, inventors. The Procter & Gamble Company, assignee. 2003. A liquid laundry conditioning composition containing a fabric-softening silicone. World Intellectual Property Organization WO 03/097778 A1. Available from: https://patents.google.com/patent/WO2003097778A1
Deryon DD德里永, Nepra SS内普拉, Thomas BB托马, inventors. L'Oreal SA, assignee. 2013. Composition comprising a nonionic surfactant, a polycondensate of ethylene oxide and of propylene oxide and a monoalcohol. China. CN 103476388 A. Available from: https://patents.google.com/patent/CN103476388A/en
Desforges M, inventor. The Procter & Gamble Company, assignee. 1972. Detergent compositions containing stabilized alpha-amylase United States. US 3,661,786 A. Available from: https://patents.google.com/patent/US3661786
Detering J, Schade C, Trieselt W, Tropsch J, inventors. BASF SE (DE), assignee. 1997. Use of vinylpyrrolidone copolymers as detergent additives, novel polymers of vinylpyrrolidone, and preparation thereof. United States. US 5,627,151 A. Available from: https://patents.google.com/patent/US5627151
Dixon TJ, Schmidt RR, Kacher ML, Koczwara CS, Tolléns FR, Evans MW, Geary NW, inventors. The Procter & Gamble Company, assignee. 2000. Shelf stable skin cleansing liquid with gel forming polymer and lipid. United States. US 6,033,680 A. Available from: https://patents.google.com/patent/US6033680
Dos Santos CR, inventor. Sweet Distribuidora , Importaco E Exportacao De Cosmeticos Ltda, assignee. 2017. Cosmetic haircare product for the straightening of hair in shampoo format. United States. US 2017/0281524 A1. Available from: https://patents.google.com/patent/US20170281524

Eadsforth CV, Sherren AJ, Selby MA, Toy R, Eckhoff WS, McAvoy DC, Matthijs E. 2006. Monitoring of environmental fingerprints of alcohol ethoxylates in Europe and Canada. *Ecotoxicology and Environmental Safety* 64:14-29. DOI: https://doi.org/10.1016/j.ecoenv.2005.06.009.

ECCC. 2019. Draft screening assessment - Poly(alkoxylates/ethers) Group. Environment and Climate Change Canada, Canada.

ECETOC. 1993. JACC report No 23: Polycarboxylate Polymers as Used in Detergents European Centre for Ecotoxicology and Toxicology of Chemicals, Brussels.

Eiting T, Kropf C, Job M, Umbreit C, Mussmann N, Benda K, Bastigkeit T, inventors. Henkel AG and Co KGaA, assignee. 2016. Machine-type dishwasher detergent containing n-based complexing agents. European Patent Office EP 3 080 237 A1. Available from: https://patents.google.com/patent/EP3080237A1/

Eriksson E, Auffarth K, Henze M, Ledin A. 2002. Characteristics of grey wastewater. *Urban Water* 4:85–104. DOI: https://doi.org/10.1016/S1462-0758(01)00064-4.

Evers MFT, Maddox TP, inventors. The Procter & Gamble Company, assignee. 2014. Liquid hand dishwashing detergent composition. United States. US 8,901,059 B2. Available from: https://patents.google.com/patent/US8901059

Fan A, Mastrull J, Simpson E, inventors. Colgate-Palmolive Company, assignee. 2014. Liquid cleaning compositions containing long-chain fatty alcohols. United States. US 8,802,607 B2. Available from: https://patents.google.com/patent/US8802607

Fan S, Kim E, Kruse TM, Vasudevan TV, inventors. Unilever PLC, N.V., assignee. 2008. Mild, moisturizing cleansing compositions. European Patent Office EP 1 771 538 B1. Available from: https://patents.google.com/patent/EP1771538B1/

Fendinger NJ, Begley WM, McAvoy DC, Eckhoff WS. 1995. Measurement of Alkyl Ethoxylate Surfactants in Natural Waters. *Environmental Science & Technology* 29:856-863. DOI: https://doi.org/10.1021/es00004a004.

Fendinger NJ, McAvoy DC, Eckhoff WS, Price BB. 1997. Environmental occurrence of polydimethylsiloxane. *Environmental Science & Technology* 31:1555-1563. DOI: https://doi.org/10.1021/es9608712.

Fernandes GE, Valenti DJ, Stenger PC, Miracle GS, Moon AP, McDonnell M, inventors. Milliken & Company, assignee. 2013. A laundry detergent composition comprising a particle having hueing agent and clay. World Intellectual Property Organization WO 2013/169536 A1. Available from: https://patents.google.com/patent/WO2013169536A1/

Fevola MJ, inventor. Johnson & Johnson Consumer Companies, Inc., assignee. 2012. Compositions comprising a polyglyceryl nonionic surfactant and a zwitterionic surfactant. United States. US 8,227,393 B2. Available from: https://patents.google.com/patent/US8227393

Fischer S, Tropsch J, Weber H, Ettl R, inventors. BASF SE, assignee. 2012. Use of tallow alcohol ethoxylates in machine dish washing. World Intellectual Property Organization WO 2012/098177 A1. Available from: https://patents.google.com/patent/WO2012098177A1/

Fossum RD, Bodet J-F, Dihora JO, Jordan GTI, Kirksey STJ, Waits LD, inventors. The Procter and Gamble Company, assignee. 2007. Fabric care compositions comprising formaldehyde scavengers. United States. US 2007/0191256 A1. Available from: https://patents.google.com/patent/US20070191256

Fox DJ, Van Blarcom D, Rubin FK, inventors. Lever Brothers Company, assignee. 1981. Aqueous high viscosity liquid dishwasher compositions. United States. US 4,260,528 A. Available from: https://patents.google.com/patent/US4260528

Freeling F, Alygizakis NA, von der Ohe PC, Slobodnik J, Oswald P, Aalizadeh R, Cirka L, Thomaidis NS, Scheurer M. 2019. Occurrence and potential environmental risk of surfactants and their transformation products discharged by wastewater treatment plants. *Science of the Total Environment* 681:475-487. DOI: https://doi.org/10.1016/j.scitotenv.2019.04.445.

Garcia-Hidalgo E, von Goetz N, Siegrist M, Hungerbuhler K. 2017. Use-patterns of personal care and household cleaning products in Switzerland. *Food and Chemical Toxicology* 99:24-39. DOI: https://doi.org/10.1016/j.fct.2016.10.030

Garcia RF, Vasuvedan TV, Post AJ, Hsu F-LG, inventors. Lever Brothers Company, Division of Conopco, Inc., assignee. 1998. Liquid detergent compostions containing structuring polymers for enhanced suspending power and good pourability. United States. US 5,750,489 A. Available from: https://patents.google.com/patent/US5750489

Gerardi AR, Fagg BS, Jackson TJ, inventors. R.J. Reynolds Tobacco Company, assignee. 2014. Cosmetic compositions comprising tobacco seed-derived component. World Intellectual Property Organization WO 2014/197427 A2. Available from: https://patents.google.com/patent/WO2014197427A2/

Gevgilili H, Liang J, inventors. L'Oreal, assignee. 2017. Hair care compositions comprising cationic compounds, starch, and silane compounds. United States. US 2017/0281522 A1. Available from: https://patents.google.com/patent/US20170281522

Giddey C, Bunter G, Tzanos D, inventors. Rhone-Electra S.A., assignee. 1991. Cosmetic products containing milk constituents. United States. US 5,053,219 A. Available from: https://patents.google.com/patent/US5053219

Gittleman D, Kornblau DS, Czarnecki RJ, inventors. Micro Powders Inc., assignee. 2015. Compositions Comprising Synthetic Waxes. United States. US 2015/0320674 A1. Available from: https://patents.google.com/patent/US20150320674

Glenn RWJ, Dunbar JC, Kacher ML, Tolléns FR, Bolich REJ, Schmidt RR, Weisgerber DJ, Eccard WE, Clapp ML, Putman CD, Hartzler KL, Husk AR, Carethers ME, inventors. The Procter & Gamble Company, assignee. 2000. Crystalline hydroxy waxes as oil in water stabilizers for skin cleansing liquid composition. United States. US 6,080,708 A. Available from: https://patents.google.com/patent/US6080708

Gomez-Berrada MP, Ficheux AS, Dahmoul Z, Roudot AC, Ferret PJ. 2017. Exposure assessment of family cosmetic products dedicated to babies, children and adults. *Food and Chemical Toxicology* 103:56-65. DOI: https://doi.org/10.1016/j.fct.2017.02.024.

Gomez Ruiz MA, Zosimova P, Koyuncu B, inventors. The Procter & Gamble Company, assignee. 2013. Liquid detergent composition comprising a hydrophobically modified cellulosic polymer. United States. US 8,512,480 B2. Available from: https://patents.google.com/patent/US8512480

Gopalkrishnan S, Guiney KM, inventors. BASF Corp., assignee. 1999. Concentrated built liquid detergents containing a dye-transfer inhibiting additive. United States. US 5,880,081 A. Available from: https://patents.google.com/patent/US5880081

Gori K, Baltsen LET, inventors. Novozymes A/S, assignee. 2016. Laundry method, use of dnase and detergent composition. World Intellectual Property Organization WO 2016/162556 A1. Available from: https://patents.google.com/patent/WO2016162556A1/

Gorlin P, Sheth N, Kinscherf K, Fleckenstein M, inventors. Colgate-Palmolive Company, assignee. 2008. Liquid detergent composition. World Intellectual Property Organization WO 2008/033585 A1. Available from: https://patents.google.com/patent/WO2008033585A1

Grit M, Molenda M, Hoffmann M, inventors. Kao Germany GmbH, assignee. 2006. Cosmetic hair composition containing cetyl PEG / PPG-10/1 dimethicone and a ceramide.

German Patent and Trademark Office DE 603 01 960 T2. Available from: https://patents.google.com/patent/DE60301960T2/

Hammond RC, Geary NW, Jones SD, inventors. The Procter & Gamble Company, assignee. 2006. Hair conditioning compositions and their use in hair coloring compositions. United States. US 6,986,886 B2. Available from: https://patents.google.com/patent/US6986886

HERA. 2004. Alcohol Ethoxysulphates (AES) Environmental Risk Assessment. Human & Environmental Risk Assessment on ingredients of European household cleaning products Brussels.

HERA. 2005. Guidance Document Methodology. Human & Environmental Risk Assessment on Ingredients of Household Cleaning Products.

HERA. 2009. Alcohol ethoxylates. Version 2.0. Human & Environmental Risk Assessment on ingredients of European household cleaning products Brussels.

HERA. 2014a. Polycarboxylates used in detergents (Part I): Polyacrylic acid homopolymers and their sodium salts (CAS 9003-04-7). Version 3.0. Human & Environmental Risk Assessment on ingredients of European household cleaning products, Brussels.

HERA. 2014b. Polycarboxylates used in detergents (Part II): Polyacrylic/maleic acid copolymers and their sodium salts (CAS 52255-49-9). Version 3.0. Human & Environmental Risk Assessment on ingredients of European household cleaning products, Brussels.

Hilvert JE, Winstel DC, inventors. The Procter & Gamble Company, assignee. 2014. Shampoo composition with associative thickeners. United States. US 2014/0348884 A1. Available from: https://patents.google.com/patent/US20140348884

Hindley MC, inventor. Croda International PLC, assignee. 2016. Hair care formulation. World Intellectual Property Organization WO 2016/189276 A1. Available from: https://patents.google.com/patent/WO2016189276A1/

Hippe T, Battermann M, Fuhr D, inventors. Henkel AG & Co. KGaA, assignee. 2012. Hair treatment agents. German Patent and Trademark Office DE 10 2010 062 612 A1. Available from: https://patents.google.com/patent/DE102010062612A1/

Hirota H, Takaya S, inventors. Kao Corporation, assignee. 1986. Shampoo composition. European Patent Office EP 0 191 564 A2. Available from: https://patents.google.com/patent/EP0191564A2/

Hoffmann M, Ning J, inventors. Kao Germany GmbH, assignee. 2016. Hair conditioning composition. World Intellectual Property Organization WO 2016/206739 A1. Available from: https://patents.google.com/patent/WO2016206739A1/

Holt NC, Shaw NS, inventors. Unilever PLC, Unilever NV, Hindustan Lever Ltd., assignee. 2006. Shampoo compositions containing cationic polymer and an anionic surfactant mixture. World Intellectual Property Organization WO 2006/058755 A1. Available from: https://patents.google.com/patent/WO2006058755A1/

Hourigan R, Mattai J, Masters J, inventors. Colgate-Palmolive Company, assignee. 2015. Cleansing compositions with polyurethane-34. United States. US 9,006,163 B2. Available from: https://patents.google.com/patent/US9006163

Hsu F-LG, Zhu S-P, Zhu Y-P, inventors. Unilever Home & Personal Care USA, assignee. 2006a. Aqueous detergent composition containing ethoxylated fatty acid di-ester United States. US 7,098,175 B2. Available from: https://patents.google.com/patent/US7098175B2/

Hsu F-LG, Zhu Y-P, Ebert C, Boudou A, Vogel RF, Hines JD, inventors. Unilever Home and Personal Care USA, assignee. 2006b. Process of making fatty alcohol based gel detergent compositions. United States. US 7,018,970 B2. Available from: https://patents.google.com/patent/US7018970

Hüffer S, Garcia Marcos A, Detering J, inventors. BASF SE, assignee. 2016. Modified polysaccharide for use in laundry detergent and for use as anti-greying agent. European Patent Office EP 3 083 702 A1. Available from: https://patents.google.com/patent/EP3083702A1/

Janchitraponvej B, Brown W, inventors. Helene Curtis Inc., assignee. 1995. Stable conditioning shampoo having a high foam level containing a silicone conditioner, a cationic quaternary acrylate copolymer, an anionic surfactant and polyethyleneimine. United States. US 5,417,965 A. Available from: https://patents.google.com/patent/US5417965

Jones KA, inventor. The Procter & Gamble Company, assignee. 1984. Liquid detergent containing polyethylene glycol. European Patent Office EP 0 106 692 A1. Available from: https://patents.google.com/patent/EP0106692A1/

Jordan SL, inventor. Union Carbide Chemicals & Plastics Technology LLC, assignee. 2013. Cationic conditioner replacements. World Intellectual Property Organization WO 2013/048780 A1. Available from: https://patents.google.com/patent/WO2013048780A1/

Kelly RJ, Roddick-Lanzilotta AD, inventors. Keratec Ltd., assignee. 2004. Personal care formulations containing keratin. World Intellectual Property Organization CIPO. CA 2 506 847 A1. Available from: https://patents.google.com/patent/CA2506847A1/

Klinkhammer ME, Valpey RSI, Thalmann BR, Jones MA, Tsibouklis J, Stone MJ, Avery RW, inventors. SC Johnson and Son Inc, assignee. 2004. Hard surface cleaners which provide improved fragrance retention properties to hard surfaces. European Patent Office EP 1 440 139 A1. Available from: https://patents.google.com/patent/EP1440139A1/

Kud A, Schulz G, Trieselt W, Hartmann H, inventors. BASF SE (DE), assignee. 1987. Use of graft copolymers of polyalkylenoxides and vinyl acetate as anti-redeposition agents in the washing and post-treatment of textiles containing synthetic fibres. European Patent Office EP 0 219 048 A2. Available from: https://patents.google.com/patent/EP0219048A2/

Lara-Martín PA, Gómez-Parra A, González-Mazo E. 2006. Development of a method for the simultaneous analysis of anionic and non-ionic surfactants and their carboxylated metabolites in environmental samples by mixed-mode liquid chromatography–mass spectrometry. *Journal of Chromatography A* 1137:188-197. DOI: https://doi.org/10.1016/j.chroma.2006.10.009.

Lara-Martin PA, Gonzalez-Mazo E, Brownawell BJ. 2011. Multi-residue method for the analysis of synthetic surfactants and their degradation metabolites in aquatic systems by liquid chromatography-time-of-flight-mass spectrometry. *Journal of Chromatography A* 1218:4799-4807. DOI: https://doi.org/10.1016/j.chroma.2011.02.031.

Lara-Martin PA, Gonzalez-Mazo E, Petrovic M, Barcelo D, Brownawell BJ. 2014. Occurrence, distribution and partitioning of nonionic surfactants and pharmaceuticals in the urbanized Long Island Sound Estuary (NY). *Marine Pollution Bulletin* 85:710-719. DOI: https://doi.org/10.1016/j.marpolbul.2014.01.022.

Leupin JA, Gosselink EP, inventors. The Procter & Gamble Company, assignee. 2002. Laundry detergent compositions with cellulosic based polymers to provide appearance and integrity benefits to fabrics laundered therewith. United States. US 6,384,011 B1. Available from: https://patents.google.com/patent/US6384011

Li B, Li W-L, Sun S-J, Qi H, Ma W-L, Liu L-Y, Zhang Z-F, Zhu N-Z, Li Y-F. 2016. The occurrence and fate of siloxanes in wastewater treatment plant in Harbin, China. *Environmental Science and Pollution Research* 23:13200-13209. DOI: https://doi.org/10.1007/s11356-016-6481-z.

Librizzi JJ, inventor. Johnson & Johnson Consumer Inc., assignee. 2002. Viscous, mild, and effective cleansing compositions. United States. US 2002/0173435 A1. Available from: https://patents.google.com/patent/US20020173435

Mabille C, Leroy E, inventors. Rhodia Opérations, assignee. 2010. Aqueous composition suitable as shampoo. European Patent Office EP 2 216 010 A1. Available from: https://patents.google.com/patent/EP2216010A1/

Machin D, van de Pas JC, inventors. Lever Brothers Company, Division of Conopco, Inc., assignee. 1992. Liquid detergent compositions containing a PEG viscosity reducing polymer. United States. US 5,108,644 A. Available from: https://patents.google.com/patent/US5108644

Malik AH, McDaniel RSJ, Urfer AD, inventors. A. E. Staley Manufacturing Company, Henkel AG and Co KGaA, assignee. 1987. Liquid hand-soap or bubble bath composition. United States. US 4,668,422 A. Available from: https://patents.google.com/patent/US4668422

Manske SD, inventor. Clariant Finance (BVI) Limited, assignee. 2004. Liquid hand dishwashing detergent. United States. US 6,800,599 B2. Available from: https://patents.google.com/patent/US6800599

Margosiak ML, Rahn MA, Paredes RM, inventors. Unilever PLC, assignee. 2009. Transparent cleansing composition. Canadian Intellectual Property Office CA 2,384,648 C. Available from: https://patents.google.com/patent/CA2384648C

Marin BC, Bergstrom JM, inventors. The Dial Corporation, assignee. 2013. Acidic gel cleaner with improved rinsing from a dried state. World Intellectual Property Organization WO 2013/090268 A1. Available from: https://patents.google.com/patent/WO2013090268A1/

Matthijs E, Holt MS, Kiewiet A, Rijs GBJ. 1999. Environmental monitoring for linear alkylbenzene sulfonate, alcohol ethoxylate, alcohol ethoxy sulfate, alcohol sulfate, and soap. *Environmental Toxicology and Chemistry* 18:2634-2644. DOI: https://doi.org/10.1002/etc.5620181133.

McAvoy DC, Dyer SD, Fendinger NJ, Eckhoff WS, Lawrence DL, Begley WM. 1998. Removal of alcohol ethoxylates, alkyl ethoxylate sulfates, and linear alkylbenzene sulfonates in wastewater treatment. *Environmental Toxicology and Chemistry* 17:1705-1711. DOI: https://doi.org/10.1002/etc.5620170909.

McAvoy DC, Eckhoff WS, Begley WM, Pessler DG. 2006. A comparison of alcohol ethoxylate environmental monitoring data using different analytical procedures. *Environmental Toxicology and Chemistry* 25:1268-1274. DOI: https://doi.org/10.1897/05-206R.1.

McDonald L, inventor. Kelite Chemicals Corporation, assignee. 1966. Detergent composition. United States. US 3,278,444 A. Available from: https://patents.google.com/patent/US3278444

Meine G, Bessler C, inventors. Henkel AG and Co KGaA, assignee. 2015. Liquid detergent with dye-transfer inhibitor properties. European Patent Office EP 2 875 108 A1. Available from: https://patents.google.com/patent/EP2875108A1/

Meralli S, Fallou B, Lesch S, inventors. L'Oreal, assignee. 2014. Cosmetic composition comprising at least one particular amphoteric polymer and at least one particular conditioning agent. United States. US 2014/0219945 A1. Available from: https://patents.google.com/patent/US20140219945

Merces A, inventor. Burt's Bees Inc, assignee. 2013. Thickener Systems For Personal Care and Other Cleansing Compositions. United States. US 2013/0252875 A1. Available from: https://patents.google.com/patent/US20130252875

Miskiel FJ, Solanki Y, inventors. CP Kelco ApS, assignee. 1999. Acidic cleaning compositions containing xanthan gum. European Patent Office EP 0 915 951 A2. Available from: https://patents.google.com/patent/EP0915951A2/

Miura K, Nishiyama N, Yamamoto A. 2008. Aquatic Environmental Monitoring of Detergent Surfactants. *Journal of Oleo Science* 57:161-170. DOI: https://doi.org/10.5650/jos.57.161.

Moeller T, Soldanski H-D, Kuech S, Noglich J, inventors. Henkel AG and Co KGaA, assignee. 2002. Multiphase cleaning composition containing naphthalene sulfonic acid/formaldehyde condensate. United States. US 6,362,154 B1. Available from: https://patents.google.com/patent/US6362154

Moffatt MR, inventor. R & C Products Pty. Ltd., assignee. 1995. Liquid dishwashing compositions. European Patent Office EP 0 682 103 A2. Available from: https://patents.google.com/patent/EP0682103A2/

Molenda M, Tietjen I, inventors. Kao Germany GmbH, assignee. 2017. Conditioning composition for hair. United States. US 9,616,012 B2. Available from: https://patents.google.com/patent/US9616012

Morrall SW, Dunphy JC, Cano ML, Evans A, McAvoy DC, Price BP, Eckhoff WS. 2006. Removal and environmental exposure of alcohol ethoxylates in US sewage treatment. *Ecotoxicology and Environmental Safety* 64:3-13. DOI: https://doi.org/10.1016/j.ecoenv.2005.07.014.

NICNAS. 2009. Full Public Report - Polyquaternium-68. File No: LTD/1403. National Industrial Chemicals Notification and Assessment Scheme, Australia.

Noor M, Lemma S, inventors. Air Products and Chemicals Inc., assignee. 2007. Personal care compositions containing functionalized polymers. United States. US 2007/0264204 A1. Available from: https://patents.google.com/patent/US20070264204

Ohtani R, Azuma M, inventors. The Procter & Gamble Company, assignee. 2016. Laundry detergent composition. World Intellectual Property Organization WO 2016/003699 A1. Available from: https://patents.google.com/patent/WO2016003699A1/

Oldenhove L, Zocchi G, Van De Gaer D, Broze G, inventors. Colgate-Palmolive Company, assignee. 2011. Liquid detergent composition comprising an acrylic polymer/viscosity control agent mixture. United States. US 7,977,296 B2. Available from: https://patents.google.com/patent/US7977296

Pan L, Scala D, Wu D, Mattai J, Boyke C, Shi M, Curley D, inventors. Colgate-Palmolive Company, assignee. 2014. Bar soap composition and method of manufacture. World Intellectual Property Organization WO 2014/088587 A1. Available from: https://patents.google.com/patent/WO2014088587A1

Parran JJJ, inventor. The Procter & Gamble Company, assignee. 1970. Detergent compositions containing particle deposition enhancing agents. United States. US 3,489,686 A. Available from: https://patents.google.com/patent/US3489686

Patron AP, Ditschun T, inventors. Senomyx Inc., Firmenich Inc., assignee. 2017. Compositions for delivering a cooling sensation. United States. US 2017/0087199 A1. Available from: https://patents.google.com/patent/US20170087199

Pauelsen F, Huppertsberg S, Knepper TP, Zahn D. 2023. Narrowing the analytical gap for water-soluble polymers: A novel trace-analytical method and first quantitative occurrence data for polyethylene oxide in surface and wastewater. *Science of The Total Environment* 882:163563. DOI: https://doi.org/10.1016/j.scitotenv.2023.163563.

Payne RK, Chupa J, inventors. Colgate-Palmolive Company, assignee. 1999. Skin cleansing composition providing enhanced perfumed deposition. World Intellectual Property Organization WO 1999/62477 A1. Available from: https://patents.google.com/patent/WO1999062477A1/

Perez-Prat Vinuesa EM, Whitely NR, Asmanidou A, Chen Q, Keuleers RRF, Van Laere A, inventors. The Procter & Gamble Company, assignee. 2014. Dishwashing method utilizing a cationic polymer/surfactant-formed coacervate. United States. US 8,883,700 B2. Available from: https://patents.google.com/patent/US8883700

Petrovic M, Gehringer P, Eschweiler H, Barceló D. 2007. Radiolytic decomposition of multi-class surfactants and their biotransformation products in sewage treatment plant effluents. *Chemosphere* 66:114-122. DOI: https://doi.org/10.1016/j.chemosphere.2006.05.008.

Popenoe DD, Morris SJI, Horn PS, Norwood KT. 1994. Determination of Alkyl Sulfates and Alkyl Ethoxysulfates in Wastewater Treatment Plant Influents and Effluents and in River Water Using Liquid Chromatography/Ion Spray Mass Spectrometry. *Analytical Chemistry* 66:1620-1629. DOI: https://doi.org/10.1021/ac00082a005.

Potgeiter IH, Buck AE, Betts MJ, inventors. Sasol Technology (Proprietary) Limited, assignee. 1999. Detergent and cleaning compositions derived from new detergent alcohols. United States. US H1,818 H. Available from: https://patents.google.com/patent/USH1818

Preston JC, inventor. Helene Curtis Industries Inc., assignee. 1986. Pearlescent shampoo and method for preparation of same. World Intellectual Property Organization WO 1986/05390 A1. Available from: https://patents.google.com/patent/WO1986005390A1/

Quenzer A, inventor. Procter & Gamble Deutschland GmbH, assignee. 2000. Hair conditioner gel containing capsules containing perfume or active ingredients. Germany. DE 19933452 A1. Available from: https://patents.google.com/patent/DE19933452A1/

Read RM, Southey HM, inventors. Unilever PLC, Unilever N.V., Conopco Inc., D/B/A Unilever, assignee. 2015. Hair conditioning composition comprising a zwitterion or proteincaeous material. World Intellectual Property Organization WO 2015/110511 A1. Available from: https://patents.google.com/patent/WO2015110511A1/

Reyes A, inventor. Rohm and Haas Company, assignee. 2011. Laundry detergent bar composition. European Patent Office EP 2 360 233 A1. Available from: https://patents.google.com/patent/EP2360233A1/

Ribery D, Penverne I, inventors. L'Oreal SA, assignee. 2003. Cosmetic bubble bath composition. United States. US 2003/0083211 A1. Available from: https://patents.google.com/patent/US20030083211

Rosser DA, inventor. Chesebrough-Ponds' USA Co., assignee. 1990. Aqueous soap composition containing ethoxylated nonionic surfactants. United States. US 4,975,218 A. Available from: https://patents.google.com/patent/US4975218

Rychłowska J, Zgoła A, Grześkowiak T, Łukaszewski Z. 2003. Isolation of poly(propylene glycol)s from water for quantitative analysis by reversed-phase liquid chromatography. *Journal of Chromatography A* 1021:11-17. DOI: https://doi.org/10.1016/j.chroma.2003.09.003.

Sabatelli PM, Brungs CA, inventors. W.R. Grace & Co., assignee. 1971. Machine dishwashing compositions containing sodium polyacrylate. United States. US 3,579,455 A. Available from: https://patents.google.com/patent/US3579455

Saito S, Takada H, inventors. Nippon Soda Co., Ltd., assignee. 2016. Detergent tablet composition for dishwasher, detergent tablet for dishwasher, and method for producing detergent tablet for dishwasher. World Intellectual Property Organization WO 2016/132735 A1. Available from: https://patents.google.com/patent/WO2016132735A1/

Salvador CR, Jiang C, Wu L, Okano T, Zhang Y, Diocos PA, Perez DSS, inventors. The Procter & Gamble Company, assignee. 2011. Cleansing bar compositions comprising a high

level of water. United States. US 8,080,503 B2. Available from: https://patents.google.com/patent/US8080503

Sanderson H, Dyer SD, Price BB, Nielsen AM, van Compernolle R, Selby M, Stanton K, Evans A, Ciarlo M, Sedlak R. 2006. Occurrence and weight-of-evidence risk assessment of alkyl sulfates, alkyl ethoxysulfates, and linear alkylbenzene sulfonates (LAS) in river water and sediments. *Science of the Total Environment* 368:695-712. DOI: https://doi.org/10.1016/j.scitotenv.2006.04.030.

Sanderson H, van Compernolle R, Dyer SD, Price BB, Nielsen AM, Selby M, Ferrer D, Stanton K. 2013. Occurrence and risk screening of alcohol ethoxylate surfactants in three US river sediments associated with wastewater treatment plants. *Science of the Total Environment* 463:600-610. DOI: https://doi.org/10.1016/j.scitotenv.2013.05.047.

Schmucker-Castner JF, Ambuter H, Snyder M, Weaver AA, Kotian S, inventors. Noveon IP Holdings Corp., assignee. 2005. Stable aqueous surfactant compositions. United States. US 6,897,253 B2. Available from: https://patents.google.com/patent/US6897253

Schneider K, Recke S, Kaiser E, Gotte S, Berkefeld H, Lassig J, Rudiger T, Lindtner O, Oltmanns J. 2019. Consumer behaviour survey for assessing exposure from consumer products: a feasibility study. *Journal of Exposure Science and Environmental Epidemiology* 29:83-94. DOI: https://doi.org/10.1038/s41370-018-0040-2.

Schramm CJJ, McMichael RO, Babo CA, inventors. Colgate-Palmolive Company, assignee. 2005. Laundry detergent composition containing a violet colorant. United States. US 2005/0148486 A1. Available from: https://patents.google.com/patent/US20050148486

Schröder FR, Schmitt M, Reichensperger U. 1999. Effect of waste water treatment technology on the elimination of anionic surfactants. *Waste Management* 19:125-131. DOI: https://doi.org/10.1016/S0956-053X(99)00012-4.

Sebillotte-Arnaud L, Guillou V, inventors. L'Oreal, assignee. 2002. Cleansing cosmetic composition. United States. US 2002/0035047 A1. Available from: https://patents.google.com/patent/US20020035047

Seidling JR, Cunningham CT, inventors. Kimberly-Clark Worldwide Inc., assignee. 2014. Foamable Sanitizing Compositions. United States. US 2014/0332562 A1. Available from: https://patents.google.com/patent/US20140332562

Seitz EPJ, Waggoner AL, Fox PS, Taylor TJ, inventors. The Dial Corporation, assignee. 2005. Compositions having enhanced deposition of a topically active compound on a surface. United States. US 6,861,397 B2. Available from: https://patents.google.com/patent/US6861397

Shih JS, Chuang J-C, Smith TE, Bires CD, Helioff MW, Login RB, inventors. ISP Investments Inc., assignee. 1992. Swellable, crosslinked polyvinylpyrrolidone and cosmetic compositions therewith. World Intellectual Property Organization WO 92/07011, WO 1992/007011 A1. Available from: https://patents.google.com/patent/WO1992007011A1/

Souter PF, Burdis JA, Boeckh D, Casado-Dominguez AL, Bittner C, Misske AM, inventors. The Procter & Gamble Company, assignee. 2006. Liquid laundry detergent compositions with modified polyethyleneimine polymers and lipase enzyme. United States. US 2006/0234895 A1. Available from: https://patents.google.com/patent/US20060234895

Staples LC, inventor. Stauffer Chemical Company, Kroger Co., assignee. 1982. Whey protein containing cosmetic formulations, process of preparing the same and a method for setting hair by using a non-hydrolyzed whey product. European Patent Office EP 0 046 326 A2. Available from: https://patents.google.com/patent/EP0046326A2/

Sturla J-M, Jia H, Yang Z, Zhou X, inventors. L'Oreal, assignee. 2013. Pearlescent conditioning composition and method for preparing the same. World Intellectual

Property Organization WO 2013/189037 A1. Available from: https://patents.google.com/patent/WO2013189037A1/

Takebayashi Y, Ishiwatari T, inventors. Sumitomo Chemical Company Ltd., assignee. 1999. Shampoo composition. United States. US 5,866,152 A. Available from: https://patents.google.com/patent/US5866152

Taylor TJ, Seitz EPJ, Fox PS, inventors. The Dial Corporation, assignee. 2002. Compositions containing a high percent saturation concentration of antibacterial agent. United States. US 6,451,748 B1. Available from: https://patents.google.com/patent/US6451748

Temple RD, Bryan WT, Willig CJ, inventors. The Procter & Gamble Company, assignee. 1978. Detergent compositions containing starch. United States. US 4,116,854 A. Available from: https://patents.google.com/patent/US4116854

Thiel DM, Wilmott JM, Kaysen JR, inventors. Dowbrands L.P., assignee. 1994. Shampoo-conditioning composition and method of making. United States. US 5,344,643 A. Available from: https://patents.google.com/patent/US5344643

Thiessies C-P, Schelges H, Khedkar BP, Joshi R, inventors. Henkel AG & Co. KGAA, assignee. 2013. Shaped soap products with a reduced content of fatty acid soaps. World Intellectual Property Organization WO 2013/131708 A1. Available from: https://patents.google.com/patent/WO2013131708A1/

Tisler S, Liang C, Carvalho PN, Bester K. 2021. Identification of more than 100 new compounds in the wastewater: Fate of polyethylene/polypropylene oxide copolymers and their metabolites in the aquatic environment. *Science of The Total Environment* 761:143228. DOI: https://doi.org/10.1016/j.scitotenv.2020.143228.

Tsaur LS, inventor. Unilever N.V., Hindustan Unilever Limited, assignee. 2012. Liquid soap compositions. World Intellectual Property Organization WO 2012/041591 A1. Available from: https://patents.google.com/patent/WO2012041591A1

Tsaur LS, Aronson MP, inventors. Unilever Home & Personal Care USA, assignee. 2003. Mild moisturizing liquids with soap-like rinse feel comprising polymer/oil blend. United States. US 6,521,573 B2. Available from: https://patents.google.com/patent/US6521573

Uehara N, Yang J-Z, inventors. The Procter & Gamble Company, assignee. 2004. Hair conditioning composition comprising three kinds of silicones. United States. US 2004/0096412 A1. Available from: https://patents.google.com/patent/US20040096412

USEPA. 2000. The ECOTOXicology Knowledgebase (ECOTOX). [updated 2022 Mar 10, accessed 2021 Dec 16] ed. United States Environmental Protection Agency, https://cfpub.epa.gov/ecotox/.

Wang J, Washington NM, Hunter KB, Boyer SL, inventors. The Procter and Gamble Company, assignee. 2003. Laundry detergent compositions with cellulosic polymers to provide appearance and integrity benefits to fabrics laundered therewith. European Patent Office EP 0 948 591 B1. Available from: https://patents.google.com/patent/EP0948591B1/

Weber H, Ettl R, Tropsch J, inventors. BASF SE, assignee. 2012. Dishwasher detergent formulations comprising a mixture of hydrophobically modified polycarboxylates and hydrophilically modified polycarboxylates. United States. US 8,262,804 B2. Available from: https://patents.google.com/patent/US8262804

Wis-Surel G, Moaddel T, inventors. Conopco Inc., assignee. 2013. Iridescent soap bars containing ethoxylated alcohols. United States. US 8,563,494 B2. Available from: https://patents.google.com/patent/US8563494

Yang L, Tsaur LS, inventors. Conopco Inc., assignee. 2012. Fragranced soap compositions. United States. US 8,133,853 B1. Available from: https://patents.google.com/patent/US8133853

Yu M余美珍, inventor. One Tian (Guangzhou) living health products Co., Ltd. , assignee. 2015. Silicon-free liquid shampoo and preparation method thereof. China. CN 104856895 A. Available from: https://patents.google.com/patent/CN104856895A/en

Yu WH, Jordan SL, Li WK, Bhattacharjee D, inventors. Dow Global Technologies Inc., assignee. 2009. Personal care compositions including polyurethane dispersions. World Intellectual Property Organization WO 2009/042572 A1. Available from: https://patents.google.com/patent/WO2009042572A1/

Zhen Y, Strickland WC, inventors. The Procter & Gamble Company, assignee. 1998. Laundry detergent compositions containing silicone emulsions United States. US 5,759,208 A. Available from: https://patents.google.com/patent/US5759208

Zhou Y, Jia H, Xu C, inventors. L'Oreal, assignee. 2017. Composition for treating keratin fibers. World Intellectual Property Organization WO 2017/185227 A1. Available from: https://patents.google.com/patent/WO2017185227A1/

Zhu Y-P, Hsu F-LG, inventors. Unilever Home & Personal Care USA, assignee. 2004. Liquid laundry detergent with emulsion layer. United States. US 6,797,685 B2. Available from: https://patents.google.com/patent/US6797685

Zofchak A, Carson JC, inventors. Alzo International Inc., assignee. 2005. Hair conditioning formulation. European Patent Office EP 1 552 807 A1. Available from: https://patents.google.com/patent/EP1552807A1/

Zofchak A, Slavashevich P, Carson J, inventors. Alzo Inc, assignee. 2006. Novel cosmetic emulsions and emulsifiers exhibiting dilatant rheological properties. United States. US 2006/0127344 A1. Available from: https://patents.google.com/patent/US20060127344
